# Empagliflozin is associated with lower cardiovascular risk compared with dipeptidyl peptidase-4 inhibitors in adults with and without cardiovascular disease: EMPagliflozin compaRative effectIveness and SafEty (EMPRISE) study results from Europe and Asia

**DOI:** 10.1186/s12933-023-01963-9

**Published:** 2023-08-31

**Authors:** Dorte Vistisen, Bendix Carstensen, Patorno Elisabetta, Stefanie Lanzinger, Elise Chia-Hui Tan, Daisuke Yabe, Dae Jung Kim, Wayne H.-H. Sheu, Cheli Melzer-Cohen, Reinhard W. Holl, Júlio Núñez, Kyoung Hwa Ha, Sigrun Halvorsen, Gisle Langslet, Avraham Karasik, Thomas Nyström, Leo Niskanen, Sonia Guleria, Riho Klement, Marc Carrasco, Johannes Foersch, Christina Shay, Lisette Koeneman, Fabian Hoti, Soulmaz Fazeli Farsani, Kamlesh Khunti, Francesco Zaccardi, Anuradhaa Subramanian, Krishnarajah Nirantharakumar, EMPRISE EU, Kimberly Brodovicz, Kristina Zint, Johannes Försch, Doron Rosenzweig, Maria Lajer, Mikkel Overgaard, Emilie Toresson Grip, Emma Söreskog, Joel Gunnarsson, Kristina Karlsdotter, Lotta Stenman, Mikko Tuovinen, Neus Valveny, Paula Casajust, Ruth Farmer, Andrew Ternouth, Anne Pernille Ofstad, East Asia Study Group, Masaomi Nangaku, Koichi Node, Yusuke Taneda, Atsutaka Yasui, Yutaka Seino, Jingbo Yi, Weiyu Lei, Sunwoo Lee, Jinhee Lee

**Affiliations:** 1 Steno Diabetes Center Copenhagen, Copenhagen, Denmark; 2 Department of Public Health, University of Copenhagen, Copenhagen, Denmark; 3 Division of Pharmacoepidemiology and Pharmacoeconomics, Department of Medicine, Brigham and Women’s Hospital and Harvard Medical School, Boston, MA USA; 4 Institute for Epidemiology and Medical Biometry, ZIBMT, Ulm University, Ulm, Germany; 5 German Centre for Diabetes Research (DZD), Munich-Neuherberg, Germany; 6 Department of Health Service Administration, China Medical University, Taichung, Taiwan; 7 Yutaka Seino Distinguished Centre for Diabetes Research, Kansai Electric Power Medical Research Institute, Kobe, Japan; 8 Department of Diabetes, Metabolism and Endocrinology/Department of Rheumatology and Clinical Immunology, Gifu University Graduate School of Medicine, Gifu, Japan; 9 Center for Healthcare Information Technology, Tokai National Higher Education and Research System, Nagoya, Japan; 10 Preemptive Food Research Centre, Gifu University Institute for Advanced Study, Gifu, Japan; 11 Department of Endocrinology and Metabolism, Ajou University School of Medicine, Suwon, South Korea; 12 Insititute of Molecular and Genomic Medicine, National Health Research Institutes, Taipei City, Taiwan; 13 Maccabi Institute for Research and Innovation, Maccabi Healthcare Services, Tel-Aviv, Israel; 14 Department of Cardiology, Hospital Clínico Universitario de Valencia, INCLIVA, Universidad de Valencia, CIBER Cardiovascular, Valencia, Spain; 15 Department of Cardiology, Oslo University Hospital Ullevål, University of Oslo, Oslo, Norway; 16 Department of Endocrinology, Morbid Obesity and Preventive Medicine, Lipid Clinic, Oslo University Hospital, Oslo, Norway; 17 Department of Clinical Science and Education, Södersjukhuset, Karolinska Institutet, Stockholm, Sweden; 18 Päijät-Häme Joint Authority for Health and Wellbeing, Päijät-Häme Central Hospital, Lahti, Finland; 19 University of Eastern Finland, Kuopio, Finland; 20 IQVIA, Goteborg, Sweden; 21 IQVIA, Tartu, Estonia; 22 Boehringer Ingelheim Pharmaceuticals, Inc., Barcelona, Spain; 23 Boehringer Ingelheim International GmbH, Ingelheim, Germany; 24 Boehringer Ingelheim Pharmaceuticals USA, 00 Ridgebury Road, Ridgefield, CT 06877 USA; 25 Lilly Deutschland GmbH, Bad Homburg, Germany; 26 IQVIA, Espoo, Finland; 27 Leicester Real World Evidence Unit, Leicester Diabetes Centre, University of Leicester, Leicester, UK; 28 Institute of Applied Health Research, University of Birmingham, Birmingham, UK; 29 Midlands Health Data Research UK, Birmingham, UK; 30 DEMAND Hub, University of Birmingham, Birmingham, UK

**Keywords:** Empagliflozin, Dipeptidyl peptidase-4 inhibitors, Type 2 diabetes, Cardiovascular disease, Heart failure, Comparative effectiveness

## Abstract

**Background:**

Studies that have reported lower risk for cardiovascular outcomes in users of Sodium–Glucose Cotransporter-2 Inhibitors (SGLT-2i) are limited by residual cofounding and lack of information on prior cardiovascular disease (CVD). This study compared risk of cardiovascular events in patients within routine care settings in Europe and Asia with type 2 diabetes (T2D) initiating empagliflozin compared to dipeptidyl peptidase-4 inhibitors (DPP-4i) stratified by pre-existing CVD and history of heart failure (HF).

**Methods and results:**

Adults initiating empagliflozin and DPP-4i in 2014–2018/19 from 11 countries in Europe and Asia were compared using propensity score matching and Cox proportional hazards regression to assess differences in rates of primary outcomes: hospitalisation for heart failure (HHF), myocardial infarction (MI), stroke; and secondary outcomes: cardiovascular mortality (CVM), coronary revascularisation procedure, composite outcome including HHF or CVM, and 3-point major adverse cardiovascular events (MACE: MI, stroke and CVM). Country-specific results were meta-analysed and pooled hazard ratios (HR) with 95% confidence intervals (CI) from random-effects models are presented.

In total, 85,244 empagliflozin/DPP4i PS-matched patient pairs were included with overall mean follow-up of 0.7 years. Among those with pre-existing CVD, lower risk was observed for HHF (HR 0.74; 95% CI 0.64–0.86), CVM (HR 0.55; 95% CI 0.38–0.80), HHF or CVM (HR 0.57; 95% CI 0.48–0.67) and stroke (HR 0.79; 95% CI 0.67–0.94) in patients initiating empagliflozin vs DPP-4i. Similar patterns were observed among patients without pre-existing CVD and those with and without pre-existing HF.

**Conclusion:**

These results from diverse patient populations in routine care settings across Europe and Asia demonstrate that initiation of empagliflozin compared to DPP-4i results in favourable cardioprotective effects regardless of pre-existing CVD or HF status.

**Supplementary Information:**

The online version contains supplementary material available at 10.1186/s12933-023-01963-9.

## Introduction

Individuals with type 2 diabetes (T2D) are at increased risk for cardiovascular disease (CVD), including angina, myocardial infarction (MI), heart failure (HF), and stroke, which increases their health care costs and risk for death [1, 2]. In randomized clinical trials (RCT), patients with T2D treated with sodium-glucose cotransporter-2 inhibitors (SGLT-2i, i.e., empagliflozin, canagliflozin, or dapagliflozin) exhibited lower risks for cardiovascular events, such as hospitalisation for heart failure (HHF), nonfatal MI, nonfatal stroke, cardiovascular mortality (CVM) and 3-point major adverse cardiovascular events (MACE: including MI, stroke, or CVM) when compared with patients using a placebo [3–5]. Further, a recent meta-analysis of six RCTs reported that patients with T2D using SGLT-2i had lower risk of CVM, a composite outcome including HHF or CVM, 3-point MACE, and all-cause mortality, compared to patients using placebo [6]. These cardioprotective effects were observed in patients with T2D with and without atherosclerotic CVD [6]. Although similar efficacy for cardiovascular risk reduction has been observed in various clinical trials, the results of these trials may not be completely generalisable to patients in the real-world settings due to differences in the patient characteristics or treatment regimens (e.g., duration of treatment and variations in adherence).

Large multi-national observational studies have also reported findings similar to those from the RCTs. The CVD-REAL [Comparative Effectiveness of Cardiovascular Outcomes in New Users of Sodium-Glucose Cotransporter-2 Inhibitors] studies compared new users of SGLT-2i with the new users of other glucose lowering agents and later with new users of dipeptidyl peptidase-4 inhibitors (DPP-4i) and observed an overall lower risk for all-cause mortality, CVM, HHF, and MACE, among SGLT-2i users [7–11]. The authors concluded that SGLT-2i may exhibit a class effect resulting in a cardioprotective effect for all SGLT-2i which could be generalized to all T2D patient populations [7, 8]. Similarly, decreased risk for HHF, all-cause mortality, and composite outcome of MI, stroke or all-cause mortality was reported in the EMPagliflozin compaRative effectIveness and SafEty (EMPRISE) study conducted in the United States (US) where empagliflozin users were compared with DPP-4i users [12]. Further, lower risks for cardiovascular events, including HHF, stroke, CVM and MACE, were reported in cohort studies conducted using healthcare data from Canada and Korea [13, 14].

A limitation of some past studies was the use of all glucose lowering drug users as comparator group, which increases residual confounding (or confounding by indication) [15]. Also, reports from RCTs indicated differences in incidence of outcomes among subgroups of patients with and without prior CVD; however, in observational studies only 13–30% of the included patients had prior CVD, limiting the ability to examine differences in risk between patient subgroups, including those with and without CVD [7–10]. Therefore, it is essential to examine differences in risk for cardiovascular outcomes between patients initiating the specific SGLT-2i empagliflozin and other treatments in a similar place in the treatment pathway specifically in individuals with higher and lower levels of CV risk (i.e., in patients with and without a history of CVD or HF) to better understand the patient populations that can benefit from the cardioprotective effects of empagliflozin. The primary objective of this study was to compare the risks of cardiovascular and HF hospitalisation events in patients with T2D initiating empagliflozin and those initiating DPP-4i with and without pre-existing CVD or HF in routine care settings across broad geographical regions in the European Union (EU) and Asia.

## Methods

The methods used in this study including outcomes and analyses are described in detail in the full study protocol registered in the EU PAS Register (EUPAS27606) [16].

### Data sources

The data for this retrospective cohort study was obtained from electronically recorded longitudinal data sources in Denmark, Finland, Germany, Israel, Japan, Norway, South Korea, Spain, Sweden, Taiwan, and the United Kingdom (UK). The different types of data sources included nationwide healthcare registers, regional quality registers, regional high-quality medical health records, and other health claims data. The data sources used in the study are described in more detail in Additional file 1: “Data sources” section. The study included locally adapted versions of International Classification of Diseases 9 (ICD-9) and 10 revision (ICD-10), Anatomical Therapeutic Chemical (ATC) codes (Additional file 1: Tables S1a and S2a).

### Study population

In each country, adults (≥ 18 years) diagnosed with T2D that initiated empagliflozin or any DPP-4i during the study period were considered eligible for the study. The study period started from the date of market authorisation of empagliflozin in the respective countries (Additional file 1: Supplemental Materials - Table S3a) until the end of data availability (i.e., December 2018 for all countries except Germany [December 2019], Japan [April 2018], South Korea and Taiwan [December 2017]). Eligible patients who initiated (had date of prescription/dispensation) the study drugs within the study period were included. The date of drug initiation was the cohort entry date (index date). Patients who were < 18 years old, had secondary or gestational diabetes mellitus, or end-stage renal disease before study entry [≤ 12 months of data before index date (i.e., baseline period)], or incomplete data on age or sex were excluded from the study cohorts in each country.

### Exposure

Patients were defined as empagliflozin initiators if they had a record of prescription/dispensation of empagliflozin during the study period and no record of prescription/dispensation of any SGLT-2i or any DPP-4i during the preceding 12 months (6 months for Germany) before the cohort entry date, i.e., in the baseline period. A similar approach was used to define initiators of any DPP-4i.

A list of all study drugs prescribed/dispensed during the study period in the countries included in this study is provided in Additional file 1: Table S2a. The duration of drug exposure and date of treatment discontinuation were determined based on the information available in each country and therefore defined separately in each country. Drug initiation was assumed to begin on the date of a prescription/dispensation (index date). The duration of exposure for each drug was extracted directly from the days’ supply information (when data were available) or derived from the dispensed amount and the daily dose. In most countries, a grace period of 100% of the calculated duration of drug exposure was applied to address the uncertainty of the actual duration of exposure [17]. Further, drug exposures overlapping in time were handled by moving the subsequent exposure by a maximum of 14 days. Periods of overlapping supplies and grace periods were combined into exposure periods. The exposures were defined using an ‘as-treated ‘(AT) approach, therefore, the follow-up was censored at discontinuation (defined as the end date of the last grace period), switch to other study drug, or concomitant use.

Patients were followed from index date to occurrence of any of the study outcomes, death, discontinuation of the initial study drug (defined as end of grace period), switch to any other study drug, initiation of concomitant use of study drugs (either as free or fixed-dose combinations), end of data availability, or end of study (31 December 2018 for all countries except Germany [31 December 2019], Japan [April 2018], South Korea and Taiwan [December 2017]), whichever occurred first.

### Outcomes

The primary study outcomes were HHF (available in all countries except in UK THIN [The Health Improvement Network, also known as IMRD [IQVIA Medical Research Data]), MI (available in all countries) and stroke (available in all countries). Secondary cardiovascular effectiveness outcomes included CVM (available in Finland, Norway, Sweden, Taiwan and UK CPRD [Clinical Practice Research Datalink]) and coronary revascularisation procedures (available in all countries except Germany and Spain). Two composite outcomes were also assessed: (1) HHF or CVM (available in Finland, Norway, Sweden and the UK CPRD); (2) MI, stroke, or CVM (i.e., 3-point MACE; available in Finland, Norway, Sweden, Taiwan and UK CPRD).

Two approaches were used to identify HHF, including use of a *broad* HHF definition (any diagnosis of HF associated with hospitalisations, specialist outpatient and primary care records, and/or a dispensation/record of high-ceiling or loop diuretics [ATC: C03C]) applied to data from Israel, Japan, South Korea, Spain, Taiwan and UK CPRD, and a *specific* HHF definition (a diagnosis of HF during hospitalisation or diagnosis of HF that led to hospitalisation, required most healthcare resources, or was coded as the main disease in hospital claims) applied to data from Denmark, Finland, Germany, Israel, Japan, Norway, Sweden and Taiwan (Additional file 1: Table S4a). The composite outcome including HHF or CVM was based only on data using the *specific* HHF definition (except where the broad HHF definition, diagnosis of HF in any position of hospitalisation, was applied in the UK CPRD since this definition was similar to the specific HHF definition used in other countries). All other study outcomes were generally defined as having a primary diagnosis (or procedures) of the condition of interest during hospitalisations, specialist outpatient, or primary care visits.

### Statistical analysis

Using country-level data, patients were matched by creating propensity score (PS) models between the exposure group (empagliflozin) and the comparator group (DPP-4i) using logistic regression based on available variables in each country. Covariates included sociodemographic, lifestyle characteristics, diabetic complications, comorbidities, comedications, and healthcare resource utilization in the logistic models to indicate the predicted treatment probability. Sociodemographic characteristics and lifestyle variables were measured at index date, healthcare resource utilization variables during ≤ 12 months before the index date, whereas the other covariates were measured in all available data for Nordic countries and UK THIN and during ≤ 12 months before the index date for all other countries (except Germany ≤ 6 months). The matching was done without replacement using a ratio of 1:1 and caliper width of 0.2 of the standard deviation of the logit of the PS. In case of multiple potential matching comparators, the first comparator in an ascending order of absolute difference in the logit of the PS was chosen; in case of a tie, comparators were chosen randomly. The matching process was evaluated by observing the absolute standardised differences (ASD). Any covariate that remained unbalanced (ASD > 0.1) [18] during the matching process was included in the outcome models [19].

Each subgroup analysis was performed separately as follows: (a) Subcohorts (e.g., CVD/No CVD) were created from the main study cohorts, (b) PS-matching was performed separately for each pair of subcohorts (empagliflozin vs DPP-4i) using baseline patient characteristics, and (c) analyses were performed on the separately PS-matched cohort. Within each country, analyses were conducted using an ‘as-treated’ approach comparing empagliflozin initiators with an active comparator group of DPP-4i initiators. The risks were compared between treatment groups with stratification of patients based on presence/absence of pre-existing CVD and HF.

All outcomes were analysed using Cox proportional hazards regression models and Hazard Ratios (HR) with 95% Confidence Intervals (CI) were presented. Overall and country-specific results were pooled via random-effects meta-analysis methods. Across the estimates, heterogeneity was assessed using the estimated total heterogeneity, Chi-square test for heterogeneity (significance level: 0.1, null hypothesis: no heterogeneity) and the *I*^2^ statistic (0% to 40%: may not be important; 30% to 60%: moderate heterogeneity; 50% to 90%: substantial heterogeneity; 75% to 100%: considerable heterogeneity) [20]. If high levels of heterogeneity existed (*I*^2^ ≥ 50% or p < 0.1), the possible reasons for heterogeneity were investigated and discussed. Additionally, sensitivity analyses were conducted using alternative outcome definitions i.e., broad, or specific definitions of HHF. Sensitivity analyses were also conducted using fixed-effect meta-analysis models.

The analyses conducted in this study were done in accordance with local laws and regulations and approvals from respective scientific/ethics/data protection committees were obtained. The country-specific analyses were conducted by independent academic/statistical groups within each country using a predefined statistical analysis plan, while the meta-analyses were conducted by IQVIA using the R language [21].

## Results

The main study population consisted of 85,244 empagliflozin/DPP4i PS-matched patient pairs in total (9765 pairs in Denmark, 11,801 in Finland, 839 in Germany, 3913 in Israel, 5592 in Japan, 6344 in Norway, 9072 in South Korea, 5865 in Spain, 15,785 in Sweden, 14,048 in Taiwan, 922 in UK CPRD, and 1298 in UK THIN) after applying the eligibility criteria (data not shown). The selected patient characteristics from all study countries by presence and absence of CVD and HF are presented in Table 1. The full patient characteristics are available in Additional file 1: Table S5a–d. The overall mean follow-up was 0.7 years. Across countries and subgroups, the mean follow-up time was generally similar between empagliflozin and DPP-4i initiators and ranged from 0.44–1.01 years and 0.41–0.94 years, respectively. The median ages were generally similar between empagliflozin and DPP-4i initiators in each CVD and HF patient subgroups (age ranges in patients with CVD, 61–69 years; without CVD, 53–64 years; with HF, 62–75 years; without HF, 56–65 years). Approximately, 43–76% of empagliflozin and DPP-4i initiators were males. The other characteristics were also similar between empagliflozin and DPP-4i initiators in each country with available data (Table 1).

**Table 1:** Selected patient characteristics after propensity score matching among initiators of empagliflozin or dipeptidyl peptidase-4 inhibitors (DPP-4i)

Country^a^	Empagliflozin/DPP-4i (n/n)	Median age (years)	Males (%)	Mean time since T2D (years)	Mean number of prior glucose-lowering drug classes (n)	Having any hospitalization (%)	Mean follow-up time (years)^a^
With CVD							
Denmark	–	–	–	–	–	–	–
Finland	5819/5819	66.00/67.00	58.72/59.03	6.31/6.43	2.23/2.25	332.86/3.30	0.65/0.86
Germany	166/166	68.39/67.67	73.49/75.90	–	–	–	–
Israel	983/983	66.87/67.19	71.92/72.33	–	–	42.22/42.12	0.71/0.69
Japan	2152/2152	66.00/66.00	74.67/73.47	–	–	60.69/62.73	0.44/0.41
Norway	2559/2559	66.00/66.00	68.46/67.72	7.28/7.40	2.40/2.41	40.41/40.33	0.71/0.89
South Korea	3444/3444	61.00/61.00	57.20/58.54	–	–	29.59/31.39	0.55/0.54
Spain	1536/1536	69.00/69.00	59.57/59.31	–	–	17.84/17.32	0.90/0.86
Sweden	8429/8429	68.00/67.00	67.01/67.83	9.54/9.56	2.61/2.62	28.66/28.06	0.53/0.81
Taiwan	3889/3889	62.15/61.53	63.10/62.00	–	–	23.58/24.09	0.47/0.47
UK CPRD	229/229	62.65/61.35	64.19/60.70	–	–	34.93/37.55	1.01/0.94
UK THIN	127/127	67.00/67.00	74.02/74.80	–	–	–	0.65/0.72
Without CVD							
Denmark	–	–	–	–	–	–	–
Finland	5874/5874	60.00/60.00	57.97/57.90	5.10/5.21	2.15/2.16	11.92/12.16	0.67/0.90
Germany	671/671	62.32/62.77	56.63/56.33	–	–	–	–
Israel	2861/2861	60.18/60.15	58.76/58.37	–	–	10.77/11.46	0.72/0.68
Japan	3437/3437	56.00/56.00	62.15/63.81	–	–	36.98/38.84	0.52/0.47
Norway	3711/3711	58.00/59.00	60.20/59.42	5.81/5.86	2.29/2.31	12.15/11.61	0.72/0.90
South Korea	5629/5629	53.00/53.00	43.06/42.94	–	–	15.46/14.94	0.56/0.53
Spain	4305/4305	64.00/64.00	59.47/59.47	–	–	0.74/0.77	0.95/0.86
Sweden	7241/7241	61.00/60.00	61.55/61.76	8.45/8.41	2.59/2.59	8.11/8.15	0.55/0.83
Taiwan	10,154/10,154	55.79/55.75	56.20/57.01	–	–	10.62/11.25	0.50/0.49
UK CPRD	476/476	55.50/56.10	57.35/59.45	–	–	11.34/10.50	1.07/0.88
UK THIN	1139/1139	59.00/59.00	57.77/58.12	–	–	–	0.65/0.72
With HF							
Denmark	–	–	–	–	–	–	–
Finland	729/729	70.00/70.00	63.65/67.49	6.99/7.20	2.25/2.30	55.14/54.32	0.62/0.87
Germany	79/79	70.79/69.04	59.49/65.82	–	–	–	–
Israel	71/71	68.59/73.04	64.79/64.79	–	–	63.38/59.15	0.63/0.66
Japan	1525/1525	67.00/68.00	73.90/71.67	–	–	60.39/62.82	0.44/0.40
Norway	326/326	68.00/68.00	72.09/70.55	7.15/7.17	2.38/2.40	53.37/50.31	0.63/0.84
South Korea	561/561	63.00/64.00	59.18/57.58	–	–	47.06/47.42	0.50/0.44
Spain	130/130	74.00/75.00	60.77/55.38	–	–	52.31/53.08	0.76/0.73
Sweden	1127/1127	70.00/70.00	71.61/70.98	9.95/10.07	2.60/2.65	46.50/45.87	0.51/0.78
Taiwan	861/861	63.21/62.23	61.44/59.47	–	–	32.29/32.29	0.44/0.45
UK CPRD	–	–	–	–	–	–	–
UK THIN	–	–	–	–	–	–	–
Without HF							
Denmark	–	–	–	–	–	–	–
Finland	10,999/10,999	63.00/63.00	58.22/58.44	5.59/5.67	2.18/2.20	20.06/20.06	0.66/0.90
Germany	763/763	62.96/63.42	59.90/58.85	–	–	–	–
Israel	3782/3782	61.81/62.02	62.14/62.40	–	–	17.95/18.11	0.72/0.70
Japan	4065/4065	58.00/58.00	64.40/66.22	–	–	40.64/42.29	0.51/0.46
Norway	5977/5977	61.00/61.00	63.11/62.41	6.36/6.47	2.34/2.35	22.32/22.70	0.72/0.90
South Korea	8510/8510	56.00/56.00	56.86/56.43	–	–	19.06/19.48	0.56/0.54
Spain	5698/5698	65.00/65.00	59.60/59.20	–	–	3.95/3.62	0.94/0.85
Sweden	14,557/14,557	64.00/64.00	64.05/64.03	8.94/8.94	2.59/2.60	17.09/16.51	0.54/0.82
Taiwan	13,186/13,186	57.32/56.91	57.89/58.46	–	–	13.03/12.80	0.50/0.49
UK CPRD	762/762	57.39/57.42	60.10/60.10	–	–	19.55/20.87	1.07/0.91
UK THIN	1270/1270	59.00/59.00	59.29/58.66	–	–	–	0.65/0.75

*CVD* cardiovascular disease, *CPRD* Clinical Practice Research Datalink, *DPP-4i* dipeptidyl peptidase-4 inhibitors, *HF* heart failure, *T2D* type 2 diabetes, *THIN* The Health Improvement Network, *UK* United Kingdom

^a^The follow-up time varied with the outcomes under study. The follow-up presented here is using as-treated approach and all-cause mortality where follow-up time is the longest

Table 2 presents the risks for cardiovascular outcomes in empagliflozin initiators compared with DPP-4i initiators stratified by pre-existing CVD. Full study results per each country is available in Additional file 1: Figs. S1–S9. Among those with and without pre-existing CVD, lower risks were observed for HHF, CVM, composite outcome of HHF or CVM, and stroke in empagliflozin initiators when compared with DPP-4i initiators.

**Table 2:** Meta-analysis for major cardiovascular outcomes in empagliflozin and DPP-4i initiators stratified by pre-existing cardiovascular disease

Outcomes^a,b^	With history of cardiovascular disease (CVD)					Without history of cardiovascular disease (CVD)				
	Empagliflozin		DPP-4i		Meta-analysis	Empagliflozin		DPP-4i		Meta-analysis
	Events	PY	Events	PY	HR (95% CI)^c^	Events	PY	Events	PY	HR (95% CI)^c^
Hospitalization for heart failure (broad definition)^d^	430	6847.38	532	6594.72	0.81 (0.71–0.92)	80	16,140.31	126	15,243.11	0.61 (0.45–0.82)
Hospitalization for heart failure (specific definition)^e^	391	17,599.16	572	21,466.81	0.72 (0.58–0.89)^f^	47	15,244.29	91	18,579.66	0.61 (0.42–0.88)
Hospitalization for heart failure (broad + specific)^g^	663	21,025.10	920	24,749.03	0.74 (0.64–0.86)	97	30,723.15	167	33,288.62	0.62 (0.48–0.80)
Cardiovascular mortality^h^	79	12,174.75	206	16,212.16	0.55 (0.38–0.80)	23	15,705.27	41	19,634.39	0.72 (0.39–1.31)
Composite outcome of hospitalization for heart failure or cardiovascular mortality^i^	183	10,281.26	425	14,201.76	0.57 (0.48–0.67)	16	10,608.68	66	14,606.81	0.35 (0.20–0.60)
Myocardial infarction (MI)^j^	250	21,258.76	295	25,146.95	1.00 (0.84–1.19)	102	33,298.33	107	36,643.56	1.09 (0.77–1.54)
Stroke^k^	242	21,189.34	312	25,060.14	0.79 (0.67–0.94)	134	33,801.77	168	37,039.94	0.90 (0.71–1.14)
3-point MACE (MI, stroke, and cardiovascular mortality)^l^	258	12,042.29	327	16,026.79	1.04 (0.83–1.31)	130	16,159.66	153	19,981.63	1.03 (0.81–1.30)
Coronary revascularization procedures^m^	529	19,544.07	581	23,478.00	1.03 (0.83–1.27)^f^	153	28,937.27	173	32,497.05	0.94 (0.75–1.17)

*CI* confidence interval, *CPRD* Clinical Practice Research Datalink, *DPP-4i* dipeptidyl peptidase-4 inhibitors, *HR* hazard ratio, *PY* person-years, *THIN* The Health Improvement Network, *UK* United Kingdom

^a^An as-treated approach was used and 100% grace period with no risk window was applied

^b^Countries with insufficient number of outcome events were omitted from the individual outcome meta-analysis. The number of countries included in each analysis therefore may vary

^c^Based on random-effects model

^d^Defined as any diagnosis of heart failure associated with hospitalizations, specialist outpatient and primary care encounters, and/or a dispensation/record of high-ceiling or loop diuretics. Includes Israel, Japan, South Korea, Spain, Taiwan and UK CPRD (in with CVD)

^e^Defined as a diagnosis of heart failure during hospitalization. Includes Denmark, Finland, Germany, Israel, Japan, Norway (in with CVD), Sweden and Taiwan (in with CVD)

^f^These analyses showed high level of heterogeneity *I*^2^ ≥ 50% or p < 0.1

^g^Includes broad definition in Japan, South Korea, Spain, Taiwan and UK CPRD (in with CVD), and specific definition in Denmark, Finland, Germany, Israel, Norway (in with CVD), Sweden

^h^Includes Finland, Norway, Sweden, Taiwan and UK CPRD (in with CVD)

^i^Includes Finland, Norway, Sweden and UK CPRD (in with CVD)

^j^Includes Denmark, Finland, Germany (in with CVD), Israel, Japan, Norway, South Korea, Spain, Sweden, Taiwan, UK CPRD (in with CVD) and UK THIN

^k^Includes Denmark, Finland, Germany (in with CVD), Israel, Japan, Norway, South Korea, Spain, Sweden, Taiwan, UK CPRD and UK THIN (in without CVD)

^l^Includes Finland, Norway, Sweden, Taiwan and UK CPRD

^m^Includes Denmark, Finland, Israel, Japan, South Korea, Norway, Sweden, Taiwan, UK CPRD and UK THIN (in with CVD)

When using a broad definition of HHF the risk for HHF was lower in empagliflozin initiators as compared with DPP-4i initiators in patients with pre-existing CVD (HR 0.81; 95% CI 0.71–0.92; Table 2), and in patients without pre-existing CVD (HR 0.61; 95% CI 0.45–0.82). Similarly, when using a specific definition of HHF the risk for HHF was lower in empagliflozin initiators as compared with DPP-4i initiators in patients with pre-existing CVD (HR 0.72; 95% CI 0.58–0.89) and among patients without pre-existing CVD (HR 0.61; 95% CI 0.42–0.88).

Table 3 presents the risks for major cardiovascular outcomes in empagliflozin initiators compared with DPP-4i initiators stratified by pre-existing HF. Full study results per each country is available in Additional file 1: Figs. S10–S18. Among patients with pre-existing HF, empagliflozin initiators had a lower risk for HHF (HR 0.79; 95% CI 0.70–0.89), for CVM (HR 0.53; 95% CI 0.31–0.92), and for composite outcome of HHF or CVM (HR 0.63 95% CI 0.50–0.79) when compared with DPP-4i initiators. Further, empagliflozin initiators had numerically lower risks for stroke when compared with DPP-4i initiators, although the difference was not statistically significant (HR 0.86; 95% CI 0.55–1.34). For all other outcomes (i.e., MI, coronary revascularisation procedures and 3-point MACE) the risks were similar between patients initiating empagliflozin compared to DPP-4i. These patterns of differences in rates across treatment groups were similar among patients without pre-existing HF (Table 3).

**Table 3:** Meta-analysis for major cardiovascular outcomes in empagliflozin and DPP-4i initiators, stratified by pre-existing heart failure

Outcomes^a,b^	With pre-existing heart failure (HF)					Without pre-existing heart failure (HF)				
	Empagliflozin		DPP-4i		RE Meta-analysis	Empagliflozin		DPP-4i		RE Meta-analysis
	Events	PY	Events	PY	HR (95% CI)^c^	Events	PY	Events	PY	HR (95% CI)^c^
Hospitalization for heart failure (broad definition)^d^	303	1370.72	374	1252.09	0.84 (0.66–1.06)	177	21,389.20	266	20,353.68	0.63 (0.44–0.88)^e^
Hospitalization for heart failure (specific definition)^f^	288	2725.00	389	3153.48	0.81 (0.69–0.95)	134	41,098.91	288	48,555.36	0.52 (0.33–0.82)^e^
Hospitalization for heart failure (broad + specific)^g^	483	3038.36	646	3402.54	0.79 (0.70–0.89)	251	51,196.32	443	57,957.65	0.55 (0.40–0.76)^e^
Cardiovascular mortality^h^	34	1615.69	89	2176.87	0.53 (0.31–0.92)	71	26,862.74	148	34,375.92	0.63 (0.47–0.85)
Composite outcome of hospitalization for heart failure or cardiovascular mortality^i^	106	1191.61	223	1690.05	0.63 (0.50–0.79)	90	20,299.44	268	27,804.26	0.47 (0.33–0.68)
Myocardial infarction (MI)^j^	52	2999.34	53	3473.32	1.16 (0.63–2.15)^e^	302	52,779.97	327	59,614.27	1.05 (0.89–1.24)
Stroke^k^	35	3109.95	51	3571.70	0.86 (0.55–1.34)	341	52,779.86	456	59,569.77	0.85 (0.73–0.98)
3-point MACE (MI, stroke, and cardiovascular mortality)^l^	64	1597.45	58	2152.65	1.34 (0.94–1.93)	323	26,703.95	426	34,147.88	1.03 (0.88–1.21)
Coronary revascularization procedures^m^	177	2334.38	179	2572.69	1.17 (0.75–1.80)^e^	500	46,333.01	575	53,681.66	0.93 (0.78–1.10)

*CI* confidence interval, *CPRD* Clinical Practice Research Datalink, *DPP-4i* dipeptidyl peptidase-4 inhibitors, *HR* hazard ratio, *PY* person-years, *THIN* The Health Improvement Network, *UK* United Kingdom

^a^An as-treated approach was used and 100% grace period with no risk window was applied

^b^Countries with insufficient number of outcome events were omitted from the individual outcome meta-analysis. The number of countries included in each analysis therefore may vary

^c^Based on random-effects model

^d^Defined as any diagnosis of heart failure associated with hospitalizations, specialist outpatient and primary care encounters, and/or a dispensation/record of high-ceiling or loop diuretics. Includes Israel, Japan, South Korea, Spain and Taiwan

^e^These analyses showed high level of heterogeneity *I*^2^ ≥ 50% or p < 0.1

^f^Defined as a diagnosis of heart failure during hospitalization or heart failure diagnosis during hospitalization that led to hospitalization, required most healthcare resources, or was coded as the main disease on the hospital claim. Includes Denmark, Finland, Germany, Israel, Japan, Norway, Sweden and Taiwan

^g^Includes broad definition in Japan, South Korea, Spain and Taiwan and specific definition in Denmark, Finland, Germany, Israel, Sweden and Norway

^h^Includes Finland, Norway, Sweden, Taiwan and UK CPRD (in without HF)

^i^Includes Finland, Norway, Sweden and UK CPRD (broad definition in without HF)

^j^Includes Denmark, Finland, Germany (in without HF), Israel (in without HF), Japan, Norway, South Korea, Spain (in without HF), Sweden, Taiwan and UK CPRD and THIN (in without HF)

^k^Includes Denmark, Finland, Germany (in without HF), Israel (in without HF), Japan, South Korea, Norway, Spain, Sweden, Taiwan and UK CPRD and THIN (in without HF)

^l^Includes Finland, Norway, Sweden, Taiwan and UK CPRD (in without HF)

^m^Includes Denmark, Finland (in without HF), Israel, Japan, South Korea, Norway (in without HF), Sweden, Taiwan and UK CPRD and THIN (in without HF)

When results were limited to only countries that applied a broad definition of HHF, empagliflozin initiators had a lower risk for HHF as compared with DPP-4i initiators among patients without pre-existing HF (HR 0.63; 95% CI 0.44–0.88; Table 3) and although similar differences were observed among patients with pre-existing HF (HR 0.84; 95% CI 0.66–1.06; Table 3), the difference was not statistically significant. Furthermore, analysis limited to countries that used a specific HHF definition also yielded lower risk among patients initiating empagliflozin compared with DPP-4i in both subgroups with pre-existing HF (HR 0.81; 95% CI 0.69–0.95) and those without pre-existing HF (HR 0.52; 95% CI 0.33–0.82).

The risk estimates obtained from the fixed-effect models were similar to those from the random-effect models in all analyses.

## Discussion

In this large multi-country study based on nationally representative observational data from diverse regions of Europe and Asia, patients with T2D initiating empagliflozin exhibited lower risks for several cardiovascular outcomes, including HHF, CVM and stroke, when compared to DPP-4i initiation. The lower risk for cardiovascular outcomes associated with empagliflozin was observed not only in patients with pre-existing CVD and HF (as previously demonstrated), but also in patients without pre-existing CVD and HF. Lower risk for HHF was also observed regardless of whether specific or broad definitions of HHF were applied. This study expands upon previous explorations of the broad impact of SGLT-2i class by demonstrating across several different geographic regions that initiation of the specific SGLT-2i empagliflozin is associated with cardiovascular benefit of regardless of patient history of CVD or HF.

Building upon existing studies, the results of this EMPRISE study in populations with pre-existing CVD and HF are generally comparable to results from the EMPA-REG OUTCOME trial, which followed-up 7020 patients with T2D and established atherosclerotic CVD for an average of 3.1 years and found empagliflozin use was associated with lower risks for CVM (HR 0.62; 95% CI 0.49–0.77) and HHF (HR 0.65; 95% CI 0.50–0.85) when compared with placebo use [3, 22]. However, few clinical trials have examined the cardiovascular benefit of SGLT-2i in patients at lower risk for CVD (i.e., those with no prior history of CVD). A meta-analysis conducted in 2021 of six RCT that assessed cardiovascular outcomes in patients where a minority of patients examined (33.8%) had pre-existing atherosclerotic CVD [6]. Results from this meta-analysis indicated lower risks for HHF (HR 0.68; 95% CI 0.61–0.76), composite outcome of HHF or CVM (HR 0.78; 95% CI 0.73–0.84), and MACE (HR 0.90; 95% CI 0.85–0.95) in SGLT-2i users as compared to placebo users with no interactions were observed in study outcomes by CVD status [6].

The results from this EMPRISE Europe and Asia study are unique since few observational, real-world studies have examined the CV benefit of the specific SGLT-2i empagliflozin in patients with and without no prior history of CVD or HF. Several previous large observational studies have examined risks of cardiovascular events associated with the overall SGLT-2i class compared to other glucose lowering drug users (metformin, sulphonylurea, thiazolidinedione, glucagon-like peptide-1 [GLP-1] receptor agonist and insulin) and DPP-4i users [7–10, 13], however, effect modification by history of CVD or HF was not examined. The CVD-REAL Nordic study examined the CV benefit of the overall SGLT-2i vs. DPP-4i classes in patients with and without pre-existing CVD (25% of included study population had pre-existing CVD) [10]. Consistent with results from the current EMPRISE study, the CVD-REAL Nordic study also reported lower risk for CVM in patients initiating SGLT-2i in the presence or absence of pre-existing CVD. The Nordic study also found lower risk for 3-point MACE only among patients with pre-existing CVD with initiation of SGLT-2i [10]. However, specific SGLT-2i types were not examined and concerns were also raised regarding bias occurring in the CVD-REAL studies since the study design allowed initiators of SGLT-2i to use other glucose lowering drugs prior to the cohort entry creating an immortal time-period [22, 23]. Subsequently in the EMPRISE (the US, and Europe and Asia) studies, extensive efforts were made to avoid such biases and minimise confounding by applying PS-matching and using active comparators [24]. The US EMPRISE study further demonstrated that initiation of empagliflozin versus DPP-4i was associated with a lower risk of HHF (HR 0.48, 95% CI 0.35–0.67), ACM (HR 0.52; 95% CI 0.38–0.72) and composite outcome of MI, stroke or all-cause mortality (HR 0.83; 95% CI 0.70–0.98) but a similar risk of MI/stroke [12]. Therefore, these finding from EMPRISE Europe and Asia that demonstrate the CV benefit of empagliflozin in patients with and without CVD, combined with other clinical and observational studies (mainly examining patients with a history of CV disease) reporting the CV benefit of all SGLT-2i, contribute to a body of evidence indicating the beneficial cardiovascular effects of empagliflozin are applicable to all adult patients with T2D, even those without prior history of CVD or HF.

Also consistent with previous studies is the lower risk for stroke as observed in our study, which was also observed in the CVD-REAL study (HR 0.68; 95% CI 0.55–0.84) [8], the study by Kohsaka et al. (HR 0.85; 95% CI 0.77–0.93) [9] and a similar Korean study (HR 0.86; 95% CI 0.77–0.97) [14]. Further, lower risk for MI was observed in the CVD-REAL study when SGLT-2i users were compared with other glucose lowering drug users (HR 0.81; 95% CI 0.74–0.88) [8] and in the Kohsaka study when SGLT-2i users were compared with DPP-4i users (HR 0.88; 95% CI 0.80–0.98) [9]. On the other hand, the CVD-REAL Nordic study and the Korean study reported that risks for MI and stroke were similar between the SGLT-2i and other glucose lowering drug users or DPP-4i users, respectively. The lower risks for 3-point MACE (HR 0.78; 95% CI 0.69–0.87) and CVM (HR 0.53; 95% CI 0.40–0.71) when SGLT-2i users were compared with other glucose lowering drug users in the CVD-REAL Nordic study are comparable with the results of the current EMPRISE study [10].

The exact mechanisms by which SGLT-2i reduce cardiovascular risks are not clearly understood but many have been hypothesized. SGLT-2i are known to possess multiple properties that include beneficial metabolic effects (e.g., they are known to cause weight reduction and decrease arterial blood pressure) that lead to improved outcomes [23–25]. These drugs may also increase potassium and magnesium levels which may impact ventricular loading conditions, direct effects on cardiac structure and function, myocardial energetics, sodium/hydrogen exchange, and have anti-atherosclerotic, anti-inflammatory effects, and also modulate endothelial function [26]. These mechanisms may also explain the associations seen in this EMPRISE study, however, no there is a paucity of studies specifically examining the underlying mechanisms by which empagliflozin reduces CV risk.

## Strengths

The foremost strength of this study is that it included patients from different clinical settings in several diverse countries across Europe and Asia. The chances of selection bias were also minimised since secondary routine data sources were used in this study. Therefore, the results of this study may be generalisable to other similar populations of patients with T2D. The previous studies mainly focused on all SGLT-2i and were able to include few empagliflozin users (empagliflozin users contributed < 7% of total exposure time in the CVD-REAL studies) [7], therefore assessment of the associations between empagliflozin use and cardiovascular outcomes may be limited in routine care settings and is available primarily from clinical trials. The patients in routine care settings may differ to those included in the clinical trials due to underlying differences in treatment regimens and comorbidities. The result of our study complements the EMPRISE US study and adds valuable information on the associations between empagliflozin use and risk for cardiovascular events compared to DPP-4i which may be useful to healthcare professionals treating patients with T2D. Further, our study examined the associations in patients with and without pre-existing CVD and HF, which is less well-examined in previous studies due to lack of sufficient number of eligible patients with cardiovascular events. Limited information is available on the risks for MI and stroke among patients with T2D using empagliflozin compared to placebo or other glucose lowering treatments due to similar limitations in existing studies and therefore the results of this study provided additional insights on the associations. An active comparator group, wherein patients who initiated a different drug for the same disease indication, was used in this study to mitigate the chances of confounding by indication, disease severity and immortal time bias [4]. Further, PS-matching and covariate adjustment in the models helped to avoid immortal time bias and reduce measured and unmeasured confounding that was likely to arise while examining the associations.

## Study limitations

Stratification of patients in EMPRISE Europe and Asia by history of CVD or HF resulted in reduced sample size and number of events in each study group and, despite similar trends in relative risk reduction observed for most study outcomes regardless of history of CVD or HF, these analyses had limited power to detect significant differences in less frequent events in patient subgroups. Confounding by indication and residual confounding also cannot be completely excluded in this study despite the PS-matching methods used. There is evidence from the EMPRISE US study [12] that (prior to matching) patients initiating empagliflozin may be younger, more frequently male and white, have lower comorbidity and frailty scores, more frequently use other types of anti-diabetic drugs, are more frequently obese, and less frequently have a history of various CVD, ischemic heart disease, HF, ischemic stroke, hypertension, and chronic kidney disease compared to patients initiating DPP-4i. Despite accounting for these patient characteristics in the current study with PS methodology, the pre-matching patient characteristics in patients observed in EMPRISE US may indicate that patients initiating empagliflozin have less severe comorbidities compared to patients initiating DPP-4i which could lead to unaccounted for residual confounding influencing the observed results in the current EMPRISE Europe and Asia study. Additionally, secondary data sources were used for this study, thus the availability and coverage of the study outcomes varied across the study countries. Each analysis was therefore conducted using data that was available in the study countries. The lack of data however may not impact the associations examined in this study except leading to slightly smaller sample sizes in some analyses, based on the assumption that the data were recorded non-differentially across patient groups within each of the study countries and since covariate balance was achieved using PS-matching. The actual drug use patterns could not be determined including use of any over-the-counter medications; therefore, some exposure misclassification is likely to have occurred. The mean follow-up time of study participants across countries ranged from 0.41 to 1.01 years across study subgroups, resulting in some patients within each country that may not have been followed long enough to observe the potential benefits of the examined treatments on all examined outcomes. Evidence from the EMPA-REG trial [27] indicates the cardiovascular benefit of empagliflozin can emerge within weeks after treatment initiation in patients with T2D and established atherosclerotic CVD, however, it may not be biologically plausible to observe the impact risk on renal or safety outcomes in patients with shorter follow-up time (e.g., 3–6 months). Therefore, future comparative effectiveness studies with longer follow-up time are needed to further validate the estimated treatment effectiveness of empagliflozin compared to DPP-4i across the broad range of effectiveness and safety outcomes. Finally, some heterogeneity was likely to exist between the study countries with respect to treatment regimens and allocation, but these were not likely to impact the study results.

## Conclusions

Findings from this large real-world study support existing evidence indicating that patients with T2D that initiate empagliflozin experience a lower risk for subsequent cardiovascular outcomes, such as HHF, CVM and stroke, when compared with patients initiating DPP-4i. Importantly, this study indicates that the cardiovascular benefit of empagliflozin was consistent regardless of whether or not patients had pre-existing CVD or HF at time of treatment initiation.

### Supplementary Information

**Additional file 1.** Additional information; Description of data sources; Diagnosis, procedure, and clinical classification codes used in the study; Study treatment marketing authorization dates; Study definitions; Patient baseline characteristics; Additional Tables 1a-5d; Additional Figures 1-18.

## Data Availability

The data that support the findings of this study are available from third party data vendors. Data sharing by the study authors is not allowed under the current data use agreements.
