# Supplementary material for: Empagliflozin is associated with lower cardiovascular risk compared with dipeptidyl peptidase-4 inhibitors in adults with and without cardiovascular disease: EMPagliflozin compaRative effectIveness and SafEty (EMPRISE) study results from Europe and Asia
Source: Cardiovasc Diabetol. 2023 Aug 31;22:233. doi: 10.1186/s12933-023-01963-9 (PMC10472675; doi:10.1186/s12933-023-01963-9)
Supplement: Supplementary file 1 — Additional file 1. Additional information; Description of data sources; Diagnosis, procedure, and clinical classification codes used in the study; Study treatment marketing authorization dates; Study definitions; Patient baseline characteristics; Additional Tables 1a-5d; Additional Figures 1-18. [file 12933_2023_1963_MOESM1_ESM.docx]

# ADDITIONAL FILE 1

**Empagliflozin is associated with lower cardiovascular risk compared with dipeptidyl peptidase-4 inhibitors in adults with and without cardiovascular disease: EMPagliflozin compaRative effectIveness and SafEty (EMPRISE) study results from Europe and Asia**

Author names: Dorte Vistisen, Bendix Carstensen, Patorno Elisabetta, Stefanie Lanzinger, Elise Chia-Hui Tan, Daisuke Yabe, Dae Jung Kim, Wayne H-H Sheu, Cheli Melzer-Cohen, Reinhard W. Holl, Júlio Núñez, Kyoung Hwa Ha, Sigrun Halvorsen, Gisle Langslet, Avraham Karasik, Thomas Nyström, Leo Niskanen, Sonia Guleria, Riho Klement, Marc Carrasco, Johannes Foersch, Christina Shay, Lisette Koeneman, Fabian Hoti, Soulmaz Fazeli Farsani, Kamlesh Khunti, Francesco Zaccardi, Anuradhaa Subramanian, Krishnarajah Nirantharakumar, EMPRISE EU and East Asia Study Group

## Data sources

### Denmark

1. Danish National Patient Register (NPR): contains information on hospital visits (secondary care) but not on primary care with general physicians or specialist care at private clinics (1). The register contains information such as date and type of admissions and outpatient visits, discharge date and diagnoses, treatments, examinations, surgeries and procedures, including Diagnosis Related Group (DRG) costs. Data is available from 1977 onwards for inpatient somatic admissions; from 1995 onwards for outpatient visits, emergency room visits and encounters at psychiatric wards; from 2002 onwards for DRG costs. International Classification of Diseases 10^th^ revision (ICD-10) codes are used since 1994 and ICD-8 codes before 1994. The register is updated at the end of December every year and has a lag of approx. 10 months.

2. Danish Register of Medicinal Products Statistics (RMPS): contains data on all prescription drugs filled at community pharmacies, but not on medications administered at hospitals (2). The register contains information from 1995 onwards on the date of purchase, item number, product name, Anatomical Therapeutic Chemical (ATC) code, strength per unit, quantity of World Health Organisation’s (WHO’s) defined daily doses (DDD) per package and number of packages filled. The register is updated twice a year at the end of June and December with a lag of approx. 5-6 months.

3. Danish Register of Causes of Death: contains information on date and cause of death since 1970 (3). ICD-10 codes are used since 1994 and ICD-8 codes before 1994. The register is updated once a year at the end of December with a lag of approx. 13 months.

4. Danish Adult Diabetes Database (Dansk Voksen Diabetes Database, DVDD): is a national clinical quality database that contains information such as diabetes diagnoses, treatment, smoking habits, body mass index (BMI), hypertension, HbA1c measurements (4). The database was established in 2006 and contains information on patients with diabetes and treated in secondary care only. Access to DVVD is subject to approval by the steering committee at the data holder.

### Finland

1. Care Register for Health Care (HILMO): The register is managed by the Finnish National Institute for Health and Welfare (THL) and has data since 1994. This national register covers virtually 100% of the Finnish population (5.5 million) (5). It contains data on secondary care (inpatient and outpatient care) and uses ICD-10 codes. The quality of the data is considered very high, but some variation in reporting and accuracy may exist e.g., especially for secondary diagnoses. The register is updated annually in September.

2. The Population Information System (Digital and Population Data Services Agency): provides information on civil status, employment status and educational level and immigration/emigration data since 1969 (from 1971 in electronic format) on Finnish and foreign citizens who are residing permanently in Finland and has virtually 100% coverage.

3. Prescription Registry of reimbursed drugs: There are two different prescription registers; the traditional Prescription register and the new e-prescription register. The traditional Prescription register is managed by the Social Insurance Institute and has data since 1994 (6). The e-prescription register is held by Social Insurance Institute and managed by THL. Use of e-prescriptions has been compulsory in public and private health care since January 2017. The data is updated quarterly and the full data for previous year is available in March. The Prescription register covers only reimbursed purchased medication but data on all purchased, also non-reimbursed, prescribed medication is recorded in the e-Prescription register. The data contents are similar in both registers comprising information on e.g., date of purchase and trade name, ATC code, strength, and package size of the drug product.

4. Cause of Death Registry: has data since 1971 and is managed by Statistics Finland (7). Key variables include hometown, sex, date of death, cause of death (ICD-10 codes have been used since 1996), and intent (in cases of injury or poisoning). The register is updated every year in December for the cause of death variable, however dates of death are available in the register earlier, with a 5-months lag time.

### Germany

Diabetes Patient Follow-up (Patienten Verlaufsdokumentation, DPV), a nationwide initiative for standardized Diabetes Documentation and quality improvement was launched in 1995 on a nationwide basis across Germany and covers more than 500 centres and more than 600,000 diabetes patients. The data on demographic and anthropometric characteristics, diabetes therapy and diabetes-related comorbidities are collected during a routine clinical examination using electronic documentation software. Every 6 months anonymised data are sent to Ulm University for data validation and analyses. A list of all previous publications using this registry is available at <https://www.d-p-v.eu>.

### Israel

Maccabi Healthcare Services (MHS) database: is the second largest state-mandated health provider in Israel with over 2 million insured patients. It was estimated that over 180,000 patients with type 2 diabetes mellitus are registered and their management is recorded in a central diabetic registry. This fully computerised database captures all information on patient interaction, including demographics, diagnoses, inpatient and outpatient visits, procedures, imaging, medications prescriptions, and actual dispenses as well as laboratory data. The Maccabi database is ideally suited for studying "real-world" use and effectiveness of empagliflozin, as the database is very detailed and complete and can allow broad and in‑depth description on the use and effect of empagliflozin.

### Japan

Medical Data Vision (MDV): is an electronic health records-based database comprised of anonymised hospital data from 374 hospitals, which cover approximately 22% of acute phase hospitals and data for 25.6 million people in Japan. The MDV data has an age distribution of the following: 14.3% of patients 0‑14 years of age, 51.0% of patients 15-64 years of age, and 34.7% of patients over 65 years old.

### Norway

1. Norwegian Patient Register: The register was established in 2008 and is managed by the Norwegian Directorate of Health [31]. It covers virtually 100% of the Norwegian population (5.3 million) and contains information regarding treatment, hospitalisation, diagnoses (ICD-10 codes) and procedures. The data is updated on an annual basis with a lag time of approximately 3-6 months. For data access a principal investigator from Norway is required and the application is processed through the Norwegian Directorate of Health.

2. Norwegian Prescription Database (NorPD): includes data since 2004 and is managed by the Norwegian Institute of Public Health [30]. It is a national database and includes information on all prescribed drugs dispensed at pharmacies such as date of purchase and trade name, ATC code, strength, defined daily dose and package size of the drug product. Information in the register is updated monthly and the complete data for the previous year is available in the month of April of the following year with a lag time of 4 months. The data can be linked to other registries and is applied for from the National Institute of Public Health.

3. Norway Control and Payment of Health Reimbursement Database Dataset Records (KUHR): is a national database that contains primary care data and includes information on dates of visits, primary and secondary diagnoses registered using International Classification of Primary Care, 2^nd^ edition (ICPC-2) codes.

4. Norwegian Causes of Death Registry: since 1951, it captures information from death certificates [33]. The registry is managed by the Norwegian Institute of Public Health and is updated annually. Key variables include age, sex, place of death, place of residence, and cause of death (ICD-10).

### South Korea

National Health Information Database (NHID): is a public database utilising information on health care utilisation, health screening, socio-demographic variables, and mortality for the whole population of South Korea. The NHID was built by the National Health Insurance System (NHIS) in 2012 using information from medical treatment and health screening records. The database includes over 50 million patients and covers data from 2002 onwards. Data collected includes demographic variables and date of death. Furthermore, part of the NHID is national health screening database and includes information on health behaviours and some bio-clinical variables. Health care resource utilisation (HCRU) information includes records on inpatient and outpatient usage (diagnosis, length of stay, treatment costs, services received) and prescription records (ATC code, days prescribed and daily dosage). NHID database is de-identified and join keys are used to replace the personal identification numbers of patients which are then used to interlink these databases.

### Spain

Valenciana Reginal Health Register: was established in 2003 and includes information on the Valencian Community. The register covers 95% of the Valencian population (approx. 5 million people). Information on all-cause mortality, hospitalisations and clinical factors drug dispensation, prescription from the primary care was obtained from the electronic health records (EHR) system of Valencia, called “ABUCASIS”. ABUCASIS is formed by GAIA and SIA. Every patient has a unique personal identification number for the health system, so there is one unique electronic centralised clinical record per patient. Total population data for the study period was extracted using the health information exchange function of ABUCASIS EHR. All information is registered in a unique electronic health recording system (ABUCASIS) which is the usual way to register clinical practice in the Valencian Health System (primary, ambulatory care, secondary care and hospital care [MBDS]). The system is linked with database of mortality register, hospital withdrawals, prescriptions and assurance databases in which each individual have a unique identification number. All diagnoses are documented in the register using ICD-9 or ICD-10 codes.

### Sweden

1. National Patient Register: is managed by the National Board of Health and Welfare and covers virtually 100% of the Swedish population (10.4 million in 2020) (8). This register includes all inpatient care since 1987 (ICD-10 from 1998) and specialised outpatient care from 2001 onwards, but it does not cover primary care and data on patients treated by health care professionals other than doctors. The register contains information on, e.g., details of hospitalisation, all disease areas, physician speciality, visit date, diagnosis, diagnosis date, comorbidities at diagnosis, (ICD-9 or -10 codes), procedures, medical treatment, and patient demographics. The register is updated annually with approx. 7-9-month lag time to data access.

2. National Dispensed Drug Register: covers virtually 100% of the Swedish population and is managed by the National Board of Health and Welfare (9). The register has data since July 2005 and is updated monthly. It contains data on drugs prescribed in primary and secondary care by community pharmacies (date of prescribing and dispensing, trade name, ATC code, strength and package size of the drug product and speciality of the prescriber). The prescribed dose and/or the indication, however, are only available as free text. The data is updated annually in April.

3. Cause of Death Register: contains data from 1961 and is updated annually (10). Key variables include age, sex, ethnicity (country of birth and residency), date of death, cause of death (ICD-9/10 codes have been used since 1988), and intent (in cases of injury or poisoning), medical procedures (if death occurred within 4 months after surgery) and autopsy. Stillbirths are not included in the register. The register is updated every year in August for the cause of death variables, however dates of death are available in the register earlier than that.

4. National Diabetes Register (NDR): The register includes adult persons (≥ 18 years) diagnosed with either type 1 diabetes mellitus (T1DM), type 2 diabetes mellitus (T2DM) or gestational diabetes (11). The register was established in 1996 and is a national quality register maintained by a regional health authority, the Region of Västra Götaland. Trained physicians and nurses report the data collected during visits to hospital outpatient clinics and primary health care centres via the internet or via clinical record databases.

The coverage of the register has increased steadily over time. For adults, the coverage is full (100%) for patient encounters in outpatient clinics, but lower (85%) in primary health care centres. The National Diabetes Register includes clinically relevant information on diabetes, including age at onset of diabetes, BMI, smoking status, HbA1c, blood pressure, cholesterol, albuminuria, and treatment of diabetes as well as common comorbidities. Access to the register data for scientific research is decided by the regional health authority after a dialogue with the authority. Applications are reviewed by a steering committee of the register.

### Taiwan:

1. National Health Insurance (NHI) database: contains data on inpatient and specialised outpatient care such as dates of visits, primary and secondary diagnoses (ICD-9, ICD-10), procedure codes, etc. It covers the whole country and is updated annually.

2. Death Registry: records information on death and covers the whole country and is updated twice per year.

3. Registry for Catastrophic Illness Patients: is a national database which includes information on illness such as T1DM, end-stage renal disease (ESRD) etc. The registry is updated twice per year.

### The United Kingdom (UK-CPRD):

The Clinical Practice Research Datalink (CPRD) contains anonymised records from 1987 onwards on approx. 13.7 million patients and 5.4 million active patients from 654 primary care UK practices, which covers an estimated 8.5% of the UK population (12). Primary care data in this study were based on the CPRD GOLD database that includes patients’ demography, clinical events, test results, prescriptions, referral to specialists and medical history. A series of additional data that were entered containing detailed information for specific predefined event types such as lifestyle indicators. The CPRD data is organised and categorised into 10 separate files which include: patient, practice, staff, consultation, clinical, referral, test, therapy, immunisation, and additional file. Each of the files contains a unique numerical patient identification code (except in the practice file which, contains numerical practice codes), which can be used to link information contained in the different files. All patients in the database have their information recorded in each of the ten files. This makes it feasible to study a broad range of patients’ medical information. All practices are required to record 95% of prescribing and patient-related events. Moreover, all data received by the CPRD goes through internal checks to ensure validation, continuity, and completeness of the data. Practice level quality assessment is expressed as “up to standard”, and at patient level by a patient acceptability flag. The patient is indicated as either acceptable or unacceptable to be used for research purposes once the entries have been checked. Regular checks that are carried out by the CPRD include weekly numbers of consultations and prescriptions, completeness of prescribing, demographic, registration details and referrals. Any data that does not meet the minimum standards are removed from the database.

Data obtained from CPRD was linked to the Office for National Statistics (ONS) Death Registration Data and the Hospital Episode Statistics (HES) Admitted Patient care data for patients from England. HES dataset contains demographic information (such as age, sex, ethnicity), details on diagnosis, interventions and administrative information (such as admission date and discharge date) of all patients that have hospital care. The diagnoses in HES are coded using the ICD-10 and the Office of Population Censuses and Surveys (OPCS) Classification of Interventions and Procedures (OPCS-4) codes. Data on inpatient hospitalisation have been available in HES since 1989, and the data are recorded for each fiscal year, with over 12 million new admissions added each year. Episodes may have up to 20 ICD-10 diagnoses, of which one is the primary code or the primary cause of admission(s), investigation(s) and treatment(s), and the remaining codes are secondary codes of diagnoses. For mortality outcome, all deaths (with date and cause) recorded on registration by the Office of National Statistics were linked with CPRD database.

### The United Kingdom (UK-THIN – also known as the IQVIA Medical Research Data [IMRD]):

Is a primary care database involving over 700 general practices (17 million patient records). Data from practices that use VISION Electronic Medical Record (EMR) are gathered, anonymised and released for research purpose. The database contains data on demographic characteristics, clinical diagnosis, physical measurement, laboratory results and prescriptions, and broadly represents the UK population. Overlapping patients between THIN and CPRD were excluded from this study.

## Table S1a. International Classification of Diseases diagnosis and procedure codes used in the study

| **Conditions** | **ICD-9 codes** | **ICD-10 codes** |
| --- | --- | --- |
| Type 2 diabetes mellitus | 250.x0, 250.x2 | E11 |
| Type 1 diabetes mellitus | 250.x1 or 250.x3 | E10 |
| Gestational diabetes | 648.8x | O24.4, O24.1 |
| Congestive heart failure | 428.x, 398.91, 402.01, 402.11, 402.91, 404.01, 404.11, 404.91, 404.03, 404.13, 404.93 | I09.81, I11.0, I13.0, I13.2, I50.1x, I50.2x, I50.3x, I50.4x, 150.9 |
| Myocardial infarction | 410.X, excluding 410.x2 | I21.0x, I21.1x, I21.2x, I21.3x, I21.4x, I22.0x, I22.0x, I22.1x, I22.2x, I22.8x, I22.9x, I25.2x |
| Stroke | 430.xx, 431.xx, 433.x1, 434.xx (excluding 434.x0), 436.x | **Subarachnoid haemorrhage:** I60xx  **Intracerebral haemorrhage:**  I610, I611, I612, I613, I614, I615, I616, I618, I619  **Occlusion and stenosis of precerebral arteries with cerebral infarction:** I6300, I63011, I63012, I63013, I63019, I6302, I63031, I63032, I63033, I63039, I6309, I6310, I63111, I63112, I63113, I63119, I6312, I63131, I63132, I63133, I63139, I6319, I6320, I63211, I63212, I63213, I63219, I6322, I63231, I63232, I63233, I63239, I6329, I6330, I6331x, I6332x, I6333x, I6334x, I6339, I6340, I6341x, I6342x, I6343x, I6344x, I6349, I6350, I6351x, I6352x, I6353x, I6354x, I6359, I636, I638, I639, I6789  **Acute, but ill-defined cerebrovascular events**: I6789 |
| Cardiovascular mortality | 390-459 | I00 to I99 |
| Coronary revascularisation procedure | **Percutaneous transluminal coronary angioplasty**, **inpatient procedure**: 00.66, 36.01, 36.02, 36.03, 36.05, 36.09  **Coronary bypass:** (no ICD-9)  **Stenting, inpatient procedure**: 36.06, 36.07  **Coronary artery bypass graft**, **inpatient procedure**: 36.1x, 36.2x  **Transmyocardial revascularisation:** (no ICD-9) | **Percutaneous transluminal coronary angioplasty:** (no ICD-10 available)  **Coronary bypass:** 02.10083, 02.1008x, 02.1009x, 02.100Ax, 02.100Jx, 02.100Kx, 02.100Zx, 02.10483, 02.10488, 02.10489, 02.1048x, 02.1049x, 02.1049x, 02.104Ax, 02.104Jx, 02.104Kx, 02.104Zx, 02.1108x, 02.1109x, 02.110Ax, 02.110Jx, 02.110Kx, 02.110Zx, 02.1148x, 02.1149x, 02.114Ax, 02.114Jx, 02.114Kx, 02.114Zx, 02.1208x, 02.1209x, 02.120Ax, 02.120Jx, 02.120Kx, 02.120Zx, 02.1248x, 02.1249x, 02.124Ax, 02.124Jx, 02.124Kx, 02.124Zx, 02.1308x, 02.1309x, 02.130Ax, 02.130Jx, 02.130Kx, 02.130Zx, 02.1348x, 02.1349x, 02.134Ax, 02.134x, 02.134Kx, 02.134Zx, 02.1K0Zx, 02.1K4Zx, 02.1L08X, 02.1L09x, 02.IL0Ax, 02.1L0Jx, 02.IL0Kx, 02.1l0Zx, 02.1L0Zx, 02.1L48X, 02.1L49x, 02.1L4xx, 02.7004x, 02.7006x, 02.7007x, 02.700Dx, 02.700Ex, 02.700Fx, 02.700Gx, 02.700Tx, 02.700Zx, 02.703xx, 02.704xx, 02.7104x, 02.7105x, 02.7106x, 02.7107x, 02.710Dx, 02.710Ex, 02.710Fx, 02.710Gx, 02.710Tx, 02.710Zx, 02.7134x, 02.7135x, 02.7136x, 02.7137x, 02.713Dx, 02.713Ex, 02.713Fx, 02.713Gx, 02.713Tx, 02.713Zx, 02.7144x, 02.7145x, 02.7146x, 02.7147x, 02.714Dx, 02.714Ex, 02.714Fx, 02.714Gx, 02.714Tx, 02.714Zx, 02.7204x, 02.72056, 02.72066, 02.7206Z, 02.72076, 02.7207Z, 02.720Dx, 02.720Ex, 02.720Fx, 02.720Tx, 02.720Zx, 02.7234x, 02.7235x, 02.7236x, 02.7237x, 02.7237x, 02.723Dx, 02.723Ex, 02.723Fx, 02.723Gx, 02.723Tx, 02.723Tx, 02.723Zx, 02.723Zx, 02.7244x, 02.7245x, 02.7246x, 02.7247x, 02.724Dx, 02.724Ex, 02.724Fx, 02.724Gx, 02.724Tx, 02.7304x, 02.7304x, 02.7305x, 02.7306x, 02.7307x, 02.730Dx, 02.730Ex, 02.730Fx, 02.730Gx, 02.730Tx, 02.730Zx, 02.7334x, 02.7335x, 02.7336x, 02.7337x, 02.733Dx, 02.733Ex, 02.733Fx, 02.733Gx, 02.733Tx, 02.733Zx, 02.7344x, 02.7345x, 02.7346x, 02.7347x, 02.734Dx, 02.734Ex, 02.734Fx, 02.734Gx, 02.734Tx, 02.734Zx, 02.C00Zx, 02.C03Zx, 02.C04Zx, 02.C10Zx, 02.C13Zx, 02.C14Zx, 02.C20Zx, 02.C23Zx, 02.C24Zx, 02.C30Zx, 02.C33Zx, 02.C34Zx  **Stenting:**  02.7004x, 02.7005x, 02.7006x, 02.7007x, 02.700Dx, 02.700Ex, 02.700Fx, 02.700Gx, 02.700Tx, 02.700Zx, 02.7034x, 02.7035x, 02.7036x, 02.7037x, 02.703Dx, 02.703Ex, 02.703Fx, 02.703Gx, 02.703Tx, 02.703Zx, 02.7044x, 02.7045x, 02.7046x, 02.7047x, 02.704Dx, 02.704Ex, 02.704Fx, 02.704Gx, 02.704Tx, 02.704Zx, 02.7104x, 02.7105x, 02.7106x, 02.7107x, 02.710Dx, 02.710Ex, 02.710Fx, 02.710Gx, 02.710Tx, 02.710Zx, 02.71346, 02.7134Z, 02.71356, 02.7135Z, 02.71366, 02.7136Z, 02.71376, 02.7137Z, 02.713D6, 02.713DZ, 02.713E6, 02.713EZ, 02.713F6, 02.713FZ, 02.713G6, 02.713GZ, 02.713T6, 02.713TZ, 02.713Z6, 02.713ZZ, 02.71446, 02.7144Z, 02.71456, 02.7145Z, 02.71466, 02.7146Z, 02.71476, 02.7147Z, 02.714D6, 02.714DZ, 02.714E6, 02.714EZ, 02.714F6, 02.714FZ, 02.714G6, 02.714GZ, 02.714T6, 02.714TZ, 02.714ZZ, 02.72046, 02.7204Z, 02.72056, 02.7205Z, 02.72066, 02.7206Z, 02.72076, 02.7207Z, 02.720D6, 02.720DZ, 02.720E6, 02.720EZ, 02.720F6, 02.720FZ, 02.720G6, 02.720GZ, 02.720T6, 02.720TZ, 02.720Z6, 02.720ZZ, 02.72346, 02.7234Z, 02.72356, 02.7235Z, 02.72366, 02.7236Z, 02.72376, 02.7237Z, 02.723D6, 02.723DZ, 02.723E6, 02.723EZ, 02.723F6, 02.723FZ, 02.723G6, 02.723GZ, 02.723T6, 02.723TZ, 02.723Z6, 02.723ZZ, 02.72446, 02.7244Z, 02.72456, 02.7245Z, 02.72466, 02.7246Z, 02.72476, 02.7247Z, 02.724D6, 02.724DZ, 02.724E6, 02.724EZ, 02.724F6, 02.724FZ, 02.724G6, 02.724T6, 02.724TZ, 02.724Z6, 02.724ZZ, 02.73046, 02.7304Z, 02.73056, 02.7305Z, 02.73066, 02.7306Z, 02.73076, 02.7307Z, 02.730D6, 02.730DZ, 02.730E6, 02.730EZ, 02.730F6, 02.730FZ, 02.730G6, 02.730GZ, 02.730T6, 02.730TZ, 02.730TZ, 02.730Z6, 02.730ZZ, 02.73346, 02.7334Z, 02.73356, 02.7335Z, 02.73366, 02.7336Z, 02.73376, 02.73376, 02.7337Z, 02.733D6, 02.733DZ, 02.733E6, 02.733EZ, 02.733F6, 02.733FZ, 02.733G6, 02.733GZ, 02.733T6, 02.733TZ, 02.733Z6, 02.733ZZ, 02.733ZZ, 02.73446, 02.7344Z, 02.73456, 02.7345Z, 02.73466, 02.73476, 02.7347Z, 02.34D6, 02.734DZ, 02.734E6, 02.734EZ, 02.734F6, 02.734FZ, 02.734G6, 02.734GZ, 02.734T6, 02.734TZ, 02.734Z6, 02.734ZZ, 02.C00Z6, 02.C03Z6, 02.C03ZZ, 02.C04Z6, 02.C10Z6, 02.C13Z6, 02.C13ZZ, 02.C14Z6, 02.C20Z6, 02.C23Z6, 02.C23ZZ, 02.C24Z6, 02.C30Z6, 02.C33ZZ, 02.C34Z6,  **Coronary artery bypass graft:** (No code available)  **Transmyocardial revascularisation:**  021K0Z5, 021K4Z5, 021L0Z5, 021L4Z5, 02QA3ZZ, 02QA4ZZ, 02QB3ZZ, 02QB4ZZ, 02QC3ZZ, 02QC4ZZ |
| Diabetic ketoacidosis | Inpatient diagnosis: 250.1x | E08.1, E09.1, E10.1, E13.1 |
| Severe hypoglycaemia | **Any-position ED or primary inpatient ICD-9 diagnosis:**  251.0, 251.1x, 251.2x, or 250.8x.  **Outcomes identified by 250.8x are not included if they co-occur with one of the following diagnoses:**  259.8, 272.7, 681.xx, 682.xx, 686.9, 707.1x, 707.2x, 707.8, 707.9, 709.3, 730.0x, 730.1x, 730.2x, 731.8. | E160, E161, E162 |

Abbreviations: ED, emergency department; ICD, International Classification of Diseases

## Table S2a. Anatomical Therapeutic Chemical classification codes used in the study

| **Sub-cohorts and study drugs** | **ATC code** |
| --- | --- |
| **Empagliflozin (including fixed-dose combinations with any other drug than DPP-4i^1^)** | |
| Empagliflozin | A10BK03 (A10BX12^2^) |
| Empagliflozin and metformin | A10BD20 |
| **Any DPP-4 inhibitor (including fixed-dose combinations with any other drug than SGLT-2i^1^)** | |
| Sitagliptin | A10BH01 |
| Sitagliptin and metformin | A10BD07 |
| Sitagliptin and pioglitazone | A10BD12 |
| Sitagliptin and simvastatin | A10BH51 |
| Vildagliptin | A10BH02 |
| Vildagliptin and metformin | A10BD08 |
| Saxagliptin | A10BH03 |
| Saxagliptin and metformin | A10BD10 |
| Alogliptin | A10BH04 |
| Alogliptin and pioglitazone | A10BD09 |
| Alogliptin and metformin | A10BD13 |
| Linagliptin | A10BH05 |
| Linagliptin and metformin | A10BD11 |
| Gemigliptin | A10BH06 |
| Gemigliptin and rosuvastatin | A10BH52 |
| Gemigliptin and metformin | A10BD18 |
| Evogliptin | A10BH07 |
| Evogliptin and metformin | A10BD22 |

Abbreviation: ATC, Anatomical Therapeutic Chemical classification; DPP-4i, dipeptidyl peptidase-4 inhibitors; SGLT-2i, sodium glucose cotransporter 2 inhibitors

1Includes combinations with other classes of antidiabetic drugs

2A previous version of the ATC code

## Table S3a. Marketing authorisations of empagliflozin, and first available DPP-4 inhibitor in each study country

| **Country** | **Date of empagliflozin MA** | **Date of first DPP-4 inhibitor MA** |
| --- | --- | --- |
| Denmark | May 2014 | March 2007 |
| Finland | May 2014 | March 2007 |
| Germany | May 2014 | March 2007 |
| Israel | November 2015 | June 2008 |
| Japan | December 2014 | October 2009 |
| Norway | May 2014 | March 2007 |
| South Korea | May2016 | September 2008 |
| Spain | February 2015 | March 2007 |
| Sweden | May 2014 | March 2007 |
| Taiwan | May 2016 | March 2009 |
| The United Kingdom | August 2014 | April 2007 |

Abbreviations: DPP-4, dipeptidyl peptidase-4; MA, marketing authorisation

## Table S4a. Definitions used in the study countries for hospitalisation for heart failure (HHF)

| **Country** | **HHF definition** | **Description** |
| --- | --- | --- |
| Denmark | Specific | HF as primary diagnosis associated with hospital admission |
|  | Broad | NA |
| Finland | Specific | HF as primary diagnosis associated with hospital admission |
|  | Broad | NA |
| Germany | Specific | HF as primary diagnosis associated with hospital admission |
|  | Broad | NA |
| Israel | Specific | Diagnosis in any position of hospitalisation |
|  | Broad | Any diagnosis associated with healthcare encounters, including hospitalisations and specialist outpatient and primary care encounters, initiating treatment with high-ceiling diuretics (loop diuretics) |
| Japan | Specific | Inpatient HF diagnosis that either required the most healthcare resources, triggered hospitalisation, or was coded as the main disease on the hospital claim |
|  | Broad | Any inpatient visit with an associated HF diagnosis code |
| Norway | Specific | HF as primary diagnosis associated with hospital admission |
|  | Broad | NA |
| South Korea | Specific | Hospitalisation with HF as primary diagnosis^1^ |
|  | Broad | HF diagnosis code in any position of hospitalisation |
| Spain | Specific | NA |
|  | Broad | Any diagnosis associated with healthcare encounters, including hospitalisations and specialist outpatient and primary care encounters |
| Sweden | Specific | HF as primary diagnosis associated with hospital admission |
|  | Broad | NA |
| Taiwan | Specific | Hospitalisation when HF was the primary diagnosis at hospitalisation |
|  | Broad | Any hospitalisation with diagnosis of HF |
| UK-CPRD | Specific | NA |
|  | Broad | Diagnosis of HF in any position of hospitalisation |
| UK THIN | Specific | NA |
|  | Broad | NA |

Abbreviations: CPRD, Clinical Practice Research Datalink; HF, heart failure; NA, not available; THIN, The Health Improvement Network; UK,

United Kingdom

^1^This definition was not used in analyses as per local expert suggestion

## Table S5a. Baseline characteristics in the empagliflozin and DPP-4i sub-cohorts after PS matching among patients without pre-existing cardiovascular disease

| **Variable** | **Denmark** | | | **Finland** | | | **Germany** | | | **Israel** | | | **Japan** | | | **South Korea** | | | **Norway** | | | **Spain** | | | **Sweden** | | | **Taiwan** | | | **UK CPRD** | | | **UK THIN** | | |
| --- | --- | --- | --- | --- | --- | --- | --- | --- | --- | --- | --- | --- | --- | --- | --- | --- | --- | --- | --- | --- | --- | --- | --- | --- | --- | --- | --- | --- | --- | --- | --- | --- | --- | --- | --- | --- |
|  | **EMPA** | **DPP4i** | **Std Diff** | **EMPA** | **DPP4i** | **Std Diff** | **EMPA** | **DPP4i** | **Std Diff** | **EMPA** | **DPP4i** | **Std Diff** | **EMPA** | **DPP4i** | **Std Diff** | **EMPA** | **DPP4i** | **Std Diff** | **EMPA** | **DPP4i** | **Std Diff** | **EMPA** | **DPP4i** | **Std Diff** | **EMPA** | **DPP4i** | **Std Diff** | **EMPA** | **DPP4i** | **Std Diff** | **EMPA** | **DPP4i** | **Std Diff** | **EMPA** | **DPP4i** | **Std Diff** |
| **Number of patients** | 5829 | 5829 |  | 5874 | 5874 |  | 671 | 671 |  | 2861 | 2861 |  | 3437 | 3437 |  | 5629 | 5629 |  | 3711 | 3711 |  | 4305 | 4305 |  | 7241 | 7241 |  | 10154 | 10154 |  | 476 | 476 |  | 1139 | 1139 |  |
| **Age at index** | | | | | | | | | | | | | | | | | | | | | | | | | | | | | | | | | | | | |
| Mean | - | - | - | 59.55 | 59.45 | 0.01 | 61.64 | 62.17 | 0.05 | 59.65 | 59.67 | 0.00 | 56 | 55.92 | 0.01 | 52.71 | 52.45 | 0.02 | 58 | 58.41 | 0.03 | 63.99 | 63.92 | 0.01 | 59.94 | 59.91 | 0.00 | 55.12 | 55.07 | 0.00 | 55.57 | 56.25 | 0.06 | 58.59 | 58.92 | 0.03 |
| SD | - | - | - | 11.26 | 11.97 | - | 10.4 | 11.36 | - | 10.6 | 10.97 | - | 13.69 | 14.22 | - | 12.1 | 12.7 | - | 12.02 | 12.23 | - | 11.01 | 11.65 | - | 11.3 | 11.71 | - | 12.34 | 13.1 | - | 11.34 | 10.94 | - | 11.18 | 12.4 | - |
| Median | - | - | - | 60 | 60 | - | 62.32 | 62.77 | - | 60.18 | 60.15 | - | 56 | 56 | - | 53 | 53 | - | 58 | 59 | - | 64 | 64 | - | 61 | 60 | - | 55.79 | 55.75 | - | 55.5 | 56.1 | - | 59 | 59 | - |
| Q1 ; Q3 | - | - | - | 52.00 ; 68.00 | 52.00 ; 68.00 | - | 54.78 ; 69.08 | 55.83 ; 70.12 | - | 52.46 ; 67.21 | 52.20 ;   67.42 | - | 46.0 ; 66.0 | 46.0 ; 67.0 | - | 45 ; 61 | 44 ; 61 | - | 50.00 ; 67.00 | 50.00 ; 67.00 | - | 57.00 ; 72.00 | 56.00 ; 72.00 | - | 52.00 ; 68.00 | 52.00 ; 69.00 | - | 46.84 ; 63.86 | 46.28 ; 63.78 | - | 48.57 ; 63.35 | 49.82 ; 63.45 | - | 51.00 ; 66.00 | 51.00 ; 67.00 | - |
| Min ; Max | - | - | - | 18.00 ; 92.00 | 18.00 ; 92.00 | - | - | - | - | 19.42 ; 93.40 | 18.61 ;   99.32 | - | 18.0 ; 90.0 | 18.0 ; 90.0 | - | 18 ; 90 | 18 ; 91 | - | 19.00 ; 97.00 | 18.00 ; 96.00 | - | 19.00 ; 97.00 | 22.00 ; 99.00 | - | 18.00 ; 94.00 | 19.00 ; 96.00 | - | 18.31 ; 94.71 | 18.06 ; 99.23 | - | 19.02 ; 86.11 | 22.57 ; 93.13 | - | 23.70 ; 89.34 | 18.55 ; 94.15 | - |
| 18-54 | - | - | - | 1886 (32.11) | 1897 (32.29) | - | 170 (25.34) | 149 (22.21) | - | 931 (32.54) | 932 (32.58) | - | 1565 (45.53) | 1578 (45.91) | - | 3035 (53.92) | 3049 (54.17) | 0.01 | 1433 (38.61) | 1384 (37.29) | - | 848 (19.70) | 841 (19.54) | - | 2272 (31.38) | 2319 (32.03) | - | 4807 (47.34) | 4845 (47.72) | - | 229 (48.11) | 222 (46.64) | 0.04 | 395 (34.68) | 424 (37.23) | - |
| 55-64 | - | - | - | 1903 (32.40) | 1870 (31.84) | - | 233 (34.72) | 245 (36.51) | - | 999 (34.92) | 982 (34.32) | - | 834 (24.27) | 801 (23.31) | - | 1720 (30.56) | 1694 (30.09) | - | 1125 (30.32) | 1135 (30.58) | - | 1327 (30.82) | 1313 (30.50) | - | 2231 (30.81) | 2220 (30.66) | - | 3171 (31.23) | 3104 (30.57) | - | 153 (32.14) | 155 (32.56) | - | 410 (36.00) | 356 (31.26) | - |
| 65-74 | - | - | - | 1613 (27.46) | 1641 (27.94) | - | 197 (29.36) | 199 (29.66) | - | 742 (25.93) | 749 (26.18) | - | 759 (22.08) | 757 (22.03) | - | 667 (11.85) | 630 (11.19) | - | 876 (23.61) | 910 (24.52) | - | 1388 (32.24) | 1421 (33.01) | - | 2135 (29.48) | 2086 (28.81) | - | 1699 (16.73) | 1585 (15.61) | - | 78 (16.39) | 84 (17.65) | - | 267 (23.44) | 254 (22.30) | - |
| 75+ | - | - | - | 472 (8.04) | 466 (7.93) | - | 71 (10.58) | 78 (11.62) | - | 189 (6.61) | 198 (6.92) | - | 279 (8.12) | 301 (8.76) | - | 207 (3.68) | 256 (4.55) | - | 277 (7.46) | 282 (7.60) | - | 742 (17.24) | 730 (16.96) | - | 603 (8.33) | 616 (8.51) | - | 477 (4.70) | 620 (6.11) | - | 16 (3.36) | 15 (3.15) | - | 67 (5.88) | 105 (9.22) | - |
| **Sex** | | | | | | | | | | | | | | | | | | | | | | | | | | | | | | | | | | | | |
| Female | - | - | - | 2469 (42.03) | 2473 (42.10) | 0.00 | 291 (43.37) | 293 (43.67) | 0.01 | 1180 (41.24) | 1191 (41.63) | 0.01 | 1301 (37.85) | 1244 (36.19) | 0.03 | 3205 (56.94) | 3212 (57.06) | 0.00 | 1477 (39.80) | 1506 (40.58) | 0.02 | 1745 (40.53) | 1745 (40.53) | 0.00 | 2784 (38.45) | 2769 (38.24) | 0.00 | 4447 (43.80) | 4365 (42.99) | 0.02 | 203 (42.65) | 193 (40.55) | 0.04 | 481 (42.23) | 477 (41.88) | 0.01 |
| Male | - | - | - | 3405 (57.97) | 3401 (57.90) | - | 380 (56.63) | 378 (56.33) | - | 1681 (58.76) | 1670 (58.37) | - | 2136 (62.15) | 2193 (63.81) | - | 2424 (43.06) | 2417 (42.94) | - | 2234 (60.20) | 2205 (59.42) | - | 2560 (59.47) | 2560 (59.47) | - | 4457 (61.55) | 4472 (61.76) | - | 5707 (56.20) | 5789 (57.01) | - | 273 (57.35) | 283 (59.45) | - | 658 (57.77) | 662 (58.12) | - |
| **HbA1c (%)** | | | | | | | | | | | | | | | | | | | | | | | | | | | | | | | | | | | | |
| Mean | - | - | - | 7.46 | 7.29 | 0.11 | 7.7 | 7.69 | 0.01 | 8.21 | 8.19 | 0.01 | 8.36 | 8.1 | 0.14 | - | - | - | - | - | - | 7.2 | 7.13 | 0.05 | 8.21 | 8.21 | 0.00 | - | - | - | 9.22 | 9.26 | 0.02 | 9.1 | 9.06 | 0.03 |
| SD | - | - | - | 3.81 | 3.79 | - | 1.4 | 1.47 | - | 1.72 | 1.82 | - | 1.91 | 1.82 | - | - | - | - | - | - | - | 1.3 | 1.33 | - | 3.58 | 3.62 | - | - | - | - | 1.51 | 1.81 | - | 1.63 | 1.56 | - |
| Median | - | - | - | 7.00 | 6.82 | - | 7.46 | 7.46 | - | 7.8 | 7.7 | - | 7.78 | 7.61 | - | - | - | - | - | - | - | 6.9 | 6.8 | - | 7.92 | 7.92 | - | - | - | - | 8.92 | 8.93 | - | 8 | 8 | - |
| Q1 ; Q3 | - | - | - | 6.27 ; 8.19 | 6.18 ; 7.82 | - | 6.77 ; 8.38 | 6.67 ; 8.45 | - | 7.00 ; 9.00 | 7.00 ; 9.00 | - | 7.0 ; 9.37 | 6.94 ; 8.65 | - | - | - | - | - | - | - | 6.30 ; 7.70 | 6.30 ; 7.60 | - | 7.27 ; 8.83 | 7.18 ; 8.92 | - | - | - | - | 8.19 ; 10.11 | 8.01 ; 10.03 | - | 7.00 ; 10.00 | 7.00 ; 10.00 | - |
| Min ; Max | - | - | - | 4.44 ; 15.78 | 3.98 ; 17.25 | - | - | - | - | 4.70 ; 16.60 | 4.30 ;   17.40 | - | 5.55 ; 18.0 | 4.8 ; 16.0 | - | - | - | - | - | - | - | 5.00 ; 16.00 | 5.00 ; 15.40 | - | 4.71 ; 15.33 | 4.99 ; 16.70 | - | - | - | - | 5.45 ; 15.78 | 5.34 ; 17.34 | - | 3.07 ; 14.78 | 3.16 ; 14.59 | - |
| Missing | - | - | - | 3472 (59.11) | 3420 (58.22) | - | - | - | - | 74 | 56 | - | 3075 (89.47) | 3131 (91.10) | - | - | - | - | - | - | - | 2497 (58.00) | 2639 (61.30) | - | - | - | - | - | - | - | - | - | - | - | - | - |
| **Total cholesterol (mg/dl)** | | | | | | | | | | | | | | | | | | | | | | | | | | | | | | | | | | | | |
| Mean | - | - | - | 184.71 | 185.28 | 0.01 | 185.08 | 190.6 | - | 180.99 | 180.33 | 0.01 | 197.21 | 192.48 | 0.12 | - | - | - | - | - | - | 183.71 | 187.65 | 0.09 | 180.57 | 180.45 | 0.00 | - | - | - | 176.12 | 176.38 | 0.01 | 173.4 | 176.78 | 0.08 |
| SD | - | - | - | 43.65 | 43.42 | - | 44.32 | 45.55 | - | 46.44 | 46.17 | - | 40.99 | 37 | - | - | - | - | - | - | - | 41.65 | 42.88 | - | 44.32 | 44.18 | - | - | - | - | 41.46 | 45.97 | - | 44.62 | 45.4 | - |
| Median | - | - | - | 177.88 | 181.75 | - | 180.5 | 185 | - | 174 | 174 | - | 195.5 | 186.12 | - | - | - | - | - | - | - | 180 | 184 | - | 174.01 | 174.01 | - | - | - | - | 170.15 | 170.15 | - | 166 | 170 | - |
| Q1 ; Q3 | - | - | - | 154.68 ; 208.82 | 154.68 ; 208.82 | - | 153.00 ; 211.00 | 157.00 ; 222.00 | - | 149.00 ;   207.00 | 147.00 ;   207.00 | - | 170.67 ; 215.17 | 167.75 ; 216.5 | - | - | - | - | - | - | - | 154.00 ; 208.00 | 159.00 ; 211.00 | - | 150.81 ; 204.95 | 150.81 ; 204.95 | - | - | - | - | 143.08 ; 201.08 | 143.08 ; 204.95 | - | 143.00 ; 201.00 | 143.00 ; 201.00 | - |
| Min ; Max | - | - | - | 73.47 ; 564.58 | 58.01 ; 498.84 | - | - | - | - | 79.00 ; 546.00 | 55.00 ;   607.00 | - | 107.0 ; 487.0 | 94.0 ; 324.0 | - | - | - | - | - | - | - | 72.00 ; 578.00 | 76.00 ; 465.00 | - | 50.27 ; 549.11 | 73.47 ; 657.39 | - | - | - | - | 88.94 ; 371.23 | 88.94 ; 355.76 | - | 61.10 ; 398.30 | 85.07 ; 402.17 | - |
| Missing | - | - | - | 3395 (57.80) | 3380 (57.54) | - | 186 (27.72) | 233 (34.72) | - | 83 | 70 | - | 3148 (91.59) | 3221 (93.72) | - | - | - | - | - | - | - | 2241 (52.10) | 2371 (55.10) | - | - | - | - | - | - | - | - | - | - | - | - | - |
| **Low-density lipoprotein level (mg/dl)** | | | | | | | | | | | | | | | | | | | | | | | | | | | | | | | | | | | | |
| Mean | - | - | - | 112 | 111.77 | 0.01 | 110.11 | 116.58 | - | 98.14 | 96.41 | 0.05 | 117.84 | 114.37 | 0.11 | - | - | - | - | - | - | 103.61 | 107.3 | 0.11 | 100.83 | 100.45 | 0.01 | - | - | - | 117.03 | 117.92 | 0.02 | 96.79 | 99.27 | 0.06 |
| SD | - | - | - | 37.2 | 37.7 | - | 36.31 | 38.63 | - | 35.62 | 35.05 | - | 33.74 | 29.34 | - | - | - | - | - | - | - | 33.99 | 33.96 | - | 37.14 | 37.02 | - | - | - | - | 42.14 | 45.9 | - | 42.13 | 43.02 | - |
| Median | - | - | - | 108.28 | 108.28 | - | 107.5 | 114 | - | 93.2 | 92.4 | - | 112.5 | 111.67 | - | - | - | - | - | - | - | 100 | 105 | - | 96.29 | 95.51 | - | - | - | - | 112.14 | 112.14 | - | 88 | 88 | - |
| Q1 ; Q3 | - | - | - | 85.07 ; 135.35 | 85.07 ; 135.35 | - | 83.00 ; 134.57 | 88.00 ; 141.00 | - | 72.00 ; 118.70 | 70.70 ;   117.70 | - | 96.78 ; 134.8 | 95.0 ; 133.0 | - | - | - | - | - | - | - | 79.00 ; 126.00 | 82.40 ; 129.00 | - | 73.47 ; 123.74 | 73.47 ; 123.74 | - | - | - | - | 87.59 ; 143.08 | 84.25 ; 146.95 | - | 65.00 ; 119.00 | 69.00 ; 121.00 | - |
| Min ; Max | - | - | - | 13.53 ; 278.42 | 7.73 ; 282.29 | - | - | - | - | 3.70 ; 273.40 | 13.10 ;   246.80 | - | 50.98 ; 291.0 | 53.0 ; 208.5 | - | - | - | - | - | - | - | 19.00 ; 261.00 | 0.00 ; 273.00 | - | 11.21 ; 309.36 | 12.76 ; 324.83 | - | - | - | - | 30.94 ; 328.69 | 15.47 ; 313.23 | - | 11.60 ; 317.09 | 15.47 ; 282.29 | - |
| Missing | - | - | - | 3481 (59.26) | 3458 (58.87) | - | 222 (33.08) | 306 (45.60) | - | 389 | 380 | - | 3174 (92.35) | 3210 (93.40) | - | - | - | - | - | - | - | 2160 (50.20) | 2391 (55.50) | - | - | - | - | - | - | - | - | - | - | - | - | - |
| **High-density lipoprotein level (mg/dl)** | | | | | | | | | | | | | | | | | | | | | | | | | | | | | | | | | | | | |
| Mean | - | - | - | 47.7 | 48.76 | 0.08 | 48.06 | 46.52 | - | 43.7 | 43.72 | 0.00 | 49.79 | 50.77 | 0.08 | - | - | - | - | - | - | 46.44 | 46.97 | 0.04 | 45.54 | 45.53 | 0.00 | - | - | - | 44.57 | 44.35 | 0.02 | 43.9 | 43.8 | 0.01 |
| SD | - | - | - | 13.38 | 14.66 | - | 22 | 13.03 | - | 10.49 | 10.44 | - | 13.05 | 11.97 | - | - | - | - | - | - | - | 12.26 | 12.35 | - | 13.32 | 13.3 | - | - | - | - | 11.32 | 11.89 | - | 12.01 | 10.93 | - |
| Median | - | - | - | 45.63 | 46.4 | - | 45 | 45 | - | 42 | 42 | - | 47.71 | 48.17 | - | - | - | - | - | - | - | 45 | 45 | - | 42.54 | 42.54 | - | - | - | - | 42.54 | 42.54 | - | 42 | 42 | - |
| Q1 ; Q3 | - | - | - | 38.28 ; 54.52 | 39.06 ; 55.68 | - | 37.45 ; 54.75 | 38.00 ; 53.00 | - | 36.00 ; 49.00 | 37.00 ;   49.00 | - | 40.72 ; 56.94 | 41.73 ; 58.8 | - | - | - | - | - | - | - | 38.00 ; 53.00 | 38.00 ; 54.00 | - | 38.67 ; 50.27 | 34.80 ; 54.14 | - | - | - | - | 37.51 ; 50.27 | 36.16 ; 50.27 | - | 34.00 ; 50.00 | 34.00 ; 50.00 | - |
| Min ; Max | - | - | - | 6.57 ; 141.15 | 8.89 ; 204.95 | - | - | - | - | 13.00 ; 97.00 | 15.00 ;   110.00 | - | 19.67 ; 98.6 | 28.0 ; 93.91 | - | - | - | - | - | - | - | 20.00 ; 124.80 | 20.00 ; 111.00 | - | 7.73 ; 170.15 | 7.73 ; 193.35 | - | - | - | - | 19.33 ; 116.01 | 15.47 ; 123.74 | - | 11.60 ; 146.95 | 19.33 ; 116.01 | - |
| Missing | - | - | - | 3406 (57.98) | 3412 (58.09) | - | 227 (33.83) | 277 (41.28) | - | 83 | 76 | - | 3125 (90.92) | 3186 (92.70) | - | - | - | - | - | - | - | 2083 (48.40) | 2335 (54.20) | - | - | - | - | - | - | - | - | - | - | - | - | - |
| **Triglyceride level (mg/dl)** | | | | | | | | | | | | | | | | | | | | | | | | | | | | | | | | | | | | |
| Mean | - | - | - | 192.47 | 184.59 | 0.05 | 208.56 | 214.42 | - | 210.93 | 210.47 | 0.00 | 201.82 | 178.23 | 0.15 | - | - | - | - | - | - | 154.74 | 153.36 | 0.02 | 196.24 | 195.96 | 0.01 | - | - | - | 201.52 | 207.65 | 0.04 | 211.39 | 217.17 | 0.04 |
| SD | - | - | - | 149.08 | 147.78 | - | 133.85 | 136.37 | - | 183.62 | 189.26 | - | 157.64 | 151.19 | - | - | - | - | - | - | - | 70.38 | 69.39 | - | 165.18 | 149.4 | - | - | - | - | 157.38 | 153.45 | - | 127.36 | 130.64 | - |
| Median | - | - | - | 159.43 | 155.88 | - | 174 | 174 | - | 168 | 170 | - | 167.8 | 142 | - | - | - | - | - | - | - | 140 | 140 | - | 159.43 | 159.43 | - | - | - | - | 173.06 | 168.29 | - | 177 | 186 | - |
| Q1 ; Q3 | - | - | - | 115.14 ; 223.20 | 108.94 ; 214.34 | - | 121.00 ; 244.00 | 128.00 ; 260.00 | - | 122.00 ;   238.00 | 120.00 ;   243.00 | - | 109.33 ; 241.5 | 100.5 ; 199.25 | - | - | - | - | - | - | - | 104.00 ; 187.00 | 100.00 ; 191.00 | - | 115.14 ; 230.28 | 115.14 ; 230.28 | - | - | - | - | 124.00 ; 239.15 | 124.00 ; 239.15 | - | 126.00 ; 256.00 | 131.00 ; 264.00 | - |
| Min ; Max | - | - | - | 29.23 ; 2941.41 | 34.54 ; 3099.06 | - | - | - | - | 40.00 ;   3490.00 | 35.00 ;   4120.00 | - | 42.0 ; 1332.0 | 34.6 ; 1783.0 | - | - | - | - | - | - | - | 41.00 ; 400.00 | 40.80 ; 389.00 | - | 8.86 ; 2949.38 | 8.86 ; 3268.23 | - | - | - | - | 0.00 ; 1771.48 | 0.00 ; 1389.73 | - | 44.29 ; 921.17 | 44.29 ; 992.03 | - |
| Missing | - | - | - | 3405 (57.97) | 3407 (58.00) | - | 200 (29.81) | 254 (37.85) | - | 84 | 69 | - | 3112 (90.54) | 3166 (92.12) | - | - | - | - | - | - | - | 2080 (48.30) | 2291 (53.20) | - | - | - | - | - | - | - | - | - | - | - | - | - |
| **Estimated Glomerular Filtration Rate (mL/min/1,73m^2^)** | | | | | | | | | | | | | | | | | | | | | | | | | | | | | | | | | | | | |
| Mean | - | - | - | 91.97 | 89.91 | 0.11 | 82.44 | 78.77 | - | 91.92 | 92.31 | 0.02 | - | - | - | - | - | - | - | - | - | 87.07 | 83.71 | 0.18 | 92.32 | 92.05 | 0.00 | - | - | - | 90.6 | 90.62 | 0.00 | 89.63 | 89.71 | 0.00 |
| SD | - | - | - | 15.87 | 20.33 | - | 19.95 | 21.81 | - | 16.97 | 18.64 | - | - | - | - | - | - | - | - | - | - | 16.94 | 20.03 | - | 16.58 | 18.31 | - | - | - | - | 17.61 | 18.05 | - | 17.19 | 17.55 | - |
| Median | - | - | - | 93.21 | 92.76 | - | 86.08 | 81.38 | - | 94.26 | 95.43 | - | - | - | - | - | - | - | - | - | - | 89.31 | 88 | - | 93.66 | 94.47 | - | - | - | - | 90 | 90 | - | 91 | 91 | - |
| Q1 ; Q3 | - | - | - | 81.57 ; 102.59 | 78.98 ; 103.36 | - | 69.43 ; 96.98 | 65.20 ; 94.72 | - | 83.02 ; 103.60 | 83.79 ;   104.63 | - | - | - | - | - | - | - | - | - | - | 77.68 ; 98.39 | 70.92 ; 98.00 | - | 83.16 ; 103.07 | 81.93 ; 104.46 | - | - | - | - | 78.00 ; 103.75 | 78.00 ; 103.49 | - | 77.00 ; 101.00 | 77.00 ; 101.00 | - |
| Min ; Max | - | - | - | 30.16 ; 157.19 | 12.37 ; 175.46 | - | - | - | - | 27.17 ; 155.93 | 19.99 ;   146.93 | - | - | - | - | - | - | - | - | - | - | 21.20 ; 271.83 | 15.73 ; 132.55 | - | 14.76 ; 157.91 | 26.83 ; 178.85 | - | - | - | - | 2.00 ; 143.04 | 26.00 ; 138.00 | - | 30.23 ; 139.28 | 28.39 ; 153.16 | - |
| Missing | - | - | - | 3098 (52.74) | 3037 (51.70) | - | 98 (14.61) | 162 (24.14) | - | 67 | 57 | - | - | - | - | - | - | - | - | - | - | 1920 (55.40) | 2452 (57.00) | - | - | - | - | - | - | - | - | - | - | - | - | - |
| **Creatinine (mg/dl)** | | | | | | | | | | | | | | | | | | | | | | | | | | | | | | | | | | | | |
| Mean | - | - | - | 0.8 | 0.84 | 0.18 | 0.91 | 0.97 | - | 0.8 | 0.8 | 0.01 | 0.77 | 0.84 | 0.12 | - | - | - | - | - | - | 0.83 | 0.88 | 0.21 | 0.8 | 0.8 | 0.00 | - | - | - | 0.81 | 0.81 | 0.02 | - | - | - |
| SD | - | - | - | 0.17 | 0.25 | - | 0.28 | 0.36 | - | 0.21 | 0.24 | - | 0.36 | 0.75 | - | - | - | - | - | - | - | 0.21 | 0.28 | - | 0.19 | 0.2 | - | - | - | - | 0.16 | 0.19 | - | - | - | - |
| Median | - | - | - | 0.79 | 0.8 | - | 0.87 | 0.9 | - | 0.77 | 0.77 | - | 0.71 | 0.73 | - | - | - | - | - | - | - | 0.8 | 0.83 | - | 0.78 | 0.78 | - | - | - | - | 0.8 | 0.79 | - | - | - | - |
| Q1 ; Q3 | - | - | - | 0.68 ; 0.90 | 0.69 ; 0.94 | - | 0.72 ; 1.03 | 0.78 ; 1.10 | - | 0.65 ; 0.92 | 0.65 ; 0.89 | - | 0.59 ; 0.87 | 0.6 ; 0.87 | - | - | - | - | - | - | - | 0.70 ; 0.94 | 0.70 ; 1.00 | - | 0.67 ; 0.90 | 0.66 ; 0.92 | - | - | - | - | 0.70 ; 0.92 | 0.69 ; 0.90 | - | - | - | - |
| Min ; Max | - | - | - | 0.36 ; 1.59 | 0.26 ; 3.62 | - | - | - | - | 0.30 ; 2.22 | 0.30 ; 2.48 | - | 0.36 ; 4.86 | 0.39 ; 9.91 | - | - | - | - | - | - | - | 0.06 ; 2.12 | 0.33 ; 3.05 | - | 0.25 ; 4.43 | 0.14 ; 2.19 | - | - | - | - | 0.42 ; 1.36 | 0.41 ; 1.76 | - | - | - | - |
| Missing | - | - | - | 3096 (52.71) | 3037 (51.70) | - | 98 (14.61) | 162 (24.14) | - | 67 | 57 | - | 3076 (89.50) | 3126 (90.95) | - | - | - | - | - | - | - | 2385 (55.40) | 1853 (57.00) | - | - | - | - | - | - | - | - | - | - | - | - | - |
| **Combined comorbidity score** | | | | | | | | | | | | | | | | | | | | | | | | | | | | | | | | | | | | |
| Mean | - | - | - | - | - | - | - | - | - | - | - | - | 0.5 | 0.48 | 0.02 | 0.22 | 0.21 | 0.01 | - | - | - | 0.08 | 0.07 | 0.01 | - | - | - | 0.49 | 0.48 | 0.00 | 0.04 | 0.05 | 0.03 | - | - | - |
| SD | - | - | - | - | - | - | - | - | - | - | - | - | 1.28 | 1.25 | - | 0.92 | 0.93 | - | - | - | - | 0.64 | 0.61 | - | - | - | - | 1.17 | 1.19 | - | 0.24 | 0.32 | - | - | - | - |
| Median | - | - | - | - | - | - | - | - | - | - | - | - | 0 | 0 | - | 0 | 0 | - | - | - | - | 0 | 0 | - | - | - | - | 0 | 0 | - | 0 | 0 | - | - | - | - |
| Q1 ; Q3 | - | - | - | - | - | - | - | - | - | - | - | - | 0.0 ; 1.0 | 0.0 ; 1.0 | - | 0 ; 1 | 0 ; 1 | - | - | - | - | 0.00 ; 0.00 | 0.00 ; 0.00 | - | - | - | - | 0.00 ; 1.00 | 0.00 ; 1.00 | - | 0.00 ; 0.00 | 0.00 ; 0.00 | - | - | - | - |
| Min ; Max | - | - | - | - | - | - | - | - | - | - | - | - | -1.0 ; 10.0 | -1.0 ; 9.0 | - | -1 ; 9 | -1 ; 6 | - | - | - | - | 0.00 ; 10.00 | 0.00 ; 7.00 | - | - | - | - | -1.00 ; 9.00 | -1.00 ; 9.00 | - | 0.00 ; 3.00 | 0.00 ; 5.00 | - | - | - | - |
| Missing | - | - | - | - | - | - | - | - | - | - | - | - | - | - | - | - | - | - | - | - | - | 0.00 (0.00) | 0.00 (0.00) | - | - | - | - | - | - | - | - | - | - | - | - | - |
| **Comorbidities and other medical conditions** | | | | | | | | | | | | | | | | | | | | | | | | | | | | | | | | | | | | |
| Diabetic retinopathy | - | - | - | 121 (2.06) | 140 (2.38) | 0.02 | 25 (3.73) | 26 (3.87) | 0.01 | 349 (12.20) | 355 (12.41) | 0.01 | 573 (16.67) | 535 (15.57) | 0.03 | 922 (16.38) | 973 (17.29) | 0.02 | 264 (7.11) | 265 (7.14) | 0.00 | 26 (0.60) | 30 (0.70) | 0.01 | 658 (9.09) | 680 (9.39) | 0.01 | 994 (9.79) | 1004 (9.89) | 0.00 | 0 (0.00) | 0 (0.00) | - | 174 (15.28) | 189 (16.59) | 0.04 |
| Diabetic neuropathy | - | - | - | 20 (0.34) | 23 (0.39) | 0.01 | 498 (74.22) | 475 (70.79) | - | 338 (11.81) | 344 (12.02) | 0.01 | 123 (3.58) | 129 (3.75) | 0.01 | 721 (12.81) | 721 (12.81) | 0.00 | 37 (1.00) | 33 (0.89) | 0.01 | 50 (1.16) | 54 (1.25) | 0.01 | 55 (0.76) | 64 (0.88) | 0.01 | 668 (6.58) | 638 (6.28) | 0.01 | 0 (0.00) | 0 (0.00) | - | 58 (5.09) | 49 (4.30) | 0.04 |
| Diabetic nephropathy | - | - | - | 17 (0.29) | 16 (0.27) | 0.00 | 78 (11.62) | 87 (12.97) | 0.05 | 151 (5.28) | 148 (5.17) | 0.00 | 274 (7.97) | 283 (8.23) | 0.01 | 736 (13.08) | 771 (13.70) | 0.02 | 22 (0.59) | 18 (0.49) | 0.01 | 16 (0.37) | 16 (0.37) | 0.00 | 29 (0.40) | 34 (0.47) | 0.01 | 2079 (20.47) | 2024 (19.93) | 0.01 | 0 (0.00) | 0 (0.00) | - | 0 (0.00) | 0 (0.00) | - |
| Diabetes with peripheral circulatory disorders | - | - | - | 10 (0.17) | 10 (0.17) | 0.00 | - | - | - | 34 (1.19) | 37 (1.29) | 0.01 | 15 (0.44) | 17 (0.49) | 0.01 | 5 (0.09) | 5 (0.09) | 0.00 | 15 (0.40) | 13 (0.35) | 0.01 | 52 (1.21) | 53 (1.23) | 0.00 | 14 (0.19) | 15 (0.21) | 0.00 | 109 (1.07) | 97 (0.96) | 0.01 | 0 (0.00) | 0 (0.00) | - | 0 (0.00) | 0 (0.00) | - |
| Diabetic foot | - | - | - | 0 (0.00) | 0 (0.00) | - | 147 (21.91) | 135 (20.12) | - | 12 (0.42) | 13 (0.45) | 0.01 | 15 (0.44) | 15 (0.44) | 0.00 | - | - | - | - | - | - | 0 (0.00) | 0 (0.00) | - | 26 (0.36) | 27 (0.37) | 0.00 | 19 (0.19) | 24 (0.24) | 0.01 | 0 (0.00) | 0 (0.00) | - | 896 (78.67) | 894 (78.49) | 0.00 |
| Hypoglycemia | - | - | - | 12 (0.20) | 9 (0.15) | 0.01 | <5 | <5 | - | 44 (1.54) | 42 (1.47) | 0.01 | 22 (0.64) | 25 (0.73) | 0.01 | 38 (0.68) | 49 (0.87) | 0.02 | <5 | 5 (0.13) | 0.01 | 35 (0.81) | 31 (0.72) | 0.01 | 38 (0.52) | 42 (0.58) | 0.01 | 45 (0.44) | 51 (0.50) | 0.01 | 0 (0.00) | 0 (0.00) | - | 5 (0.44) | <5 | 0.03 |
| Hyperlipidemia | - | - | - | 397 (6.76) | 405 (6.89) | 0.01 | 479 (71.39) | 469 (69.90) | - | 753 (26.32) | 781 (27.30) | 0.02 | 1754 (51.03) | 1655 (48.15) | 0.06 | 4659 (82.77) | 4594 (81.61) | 0.03 | 293 (7.90) | 297 (8.00) | 0.00 | 507 (11.78) | 507 (11.78) | 0.00 | 814 (11.24) | 831 (11.48) | 0.01 | 7143 (70.35) | 7210 (71.01) | 0.01 | 122 (25.63) | 124 (26.05) | 0.01 | 0 (0.00) | 0 (0.00) | - |
| Ischemic heart disease | - | - | - | 0 (0.00) | 0 (0.00) | - | - | - | - | 0 (0.00) | 0 (0.00) | - | 300 (8.73) | 299 (8.70) | 0.00 | 165 (2.93) | 149 (2.65) | 0.02 | 0 (0.00) | 0 (0.00) | - | 0 (0.00) | 0 (0.00) | - | 0 (0.00) | 0 (0.00) | - | 0 (0.00) | 0 (0.00) | 0.00 | 0 (0.00) | 0 (0.00) | - | 14 (1.23) | 17 (1.49) | 0.02 |
| Stable angina | - | - | - | 0 (0.00) | 0 (0.00) | - | - | - | - | 0 (0.00) | 0 (0.00) | - | 297 (8.64) | 295 (8.58) | 0.00 | 25 (0.44) | 26 (0.46) | 0.00 | 0 (0.00) | 0 (0.00) | - | 0 (0.00) | 0 (0.00) | - | 0 (0.00) | 0 (0.00) | - | 0 (0.00) | 0 (0.00) | 0.00 | 0 (0.00) | 0 (0.00) | - | 7 (0.61) | 9 (0.79) | 0.02 |
| History of CABG or PTCA | - | - | - | 0 (0.00) | <5 | 0.02 | 0 (0.00) | 0 (0.00) | - | 0 (0.00) | 0 (0.00) | - | 0 (0.00) | 0 (0.00) | 0.00 | <5 | 0 (0.00) | 0.02 | <5 | <5 | 0.00 | 0 (0.00) | 0 (0.00) | - | 5 (0.07) | 5 (0.07) | 0.00 | 0 (0.00) | 0 (0.00) | 0.00 | - | - | - | 0 (0.00) | 0 (0.00) | - |
| Any stroke | - | - | - | 0 (0.00) | 0 (0.00) | - | 0 (0.00) | 0 (0.00) | - | 0 (0.00) | 0 (0.00) | - | 63 (1.83) | 55 (1.60) | 0.02 | 41 (0.73) | 40 (0.71) | 0.00 | 0 (0.00) | 0 (0.00) | - | 0 (0.00) | 0 (0.00) | - | 0 (0.00) | 0 (0.00) | - | 418 (4.12) | 444 (4.37) | 0.01 | 0 (0.00) | 0 (0.00) | - | 0 (0.00) | 0 (0.00) | - |
| CHF | - | - | - | 0 (0.00) | 0 (0.00) | - | - | - | - | 0 (0.00) | 0 (0.00) | - | 0 (0.00) | 0 (0.00) | 0.00 | 38 (0.68) | 31 (0.55) | 0.02 | 0 (0.00) | 0 (0.00) | - | 0 (0.00) | 0 (0.00) | - | 0 (0.00) | 0 (0.00) | - | 0 (0.00) | 0 (0.00) | 0.00 | 0 (0.00) | 0 (0.00) | - | <5 | 7 (0.61) | 0.04 |
| Peripheral Vascular disease or surgery | - | - | - | 8 (0.14) | 9 (0.15) | 0.00 | - | - | - | 0 (0.00) | 0 (0.00) | - | 0 (0.00) | 0 (0.00) | 0.00 | 255 (4.53) | 239 (4.25) | 0.01 | 351 (9.46) | 358 (9.65) | 0.01 | 0 (0.00) | 0 (0.00) | - | 17 (0.23) | 20 (0.28) | 0.01 | 0 (0.00) | 0 (0.00) | 0.00 | 0 (0.00) | 0 (0.00) | - | <5 | <5 | 0.02 |
| Hypertension | - | - | - | 0 (0.00) | 0 (0.00) | - | 570 (84.95) | 548 (81.67) | - | 860 (30.06) | 869 (30.37) | 0.01 | 1791 (52.11) | 1763 (51.29) | 0.02 | 2844 (50.52) | 2819 (50.08) | 0.01 | 0 (0.00) | 0 (0.00) | - | 0 (0.00) | 0 (0.00) | - | 0 (0.00) | 0 (0.00) | - | 6128 (60.35) | 6191 (60.97) | 0.01 | 267 (56.09) | 271 (56.93) | 0.02 | 75 (6.58) | 66 (5.79) | 0.03 |
| Chronic kidney disease | - | - | - | <5 | <5 | 0.00 | - | - | - | 53 (1.85) | 60 (2.10) | 0.02 | 66 (1.92) | 72 (2.09) | 0.01 | 50 (0.89) | 56 (0.99) | 0.01 | 12 (0.32) | 7 (0.19) | 0.03 | 10 (0.23) | 13 (0.30) | 0.01 | 8 (0.11) | 9 (0.12) | 0.00 | 541 (5.33) | 541 (5.33) | 0.00 | 0 (0.00) | 0 (0.00) | - | <5 | <5 | 0.02 |
| **Number of antidiabetic substances at index date** | | | | | | | | | | | | | | | | | | | | | | | | | | | | | | | | | | | | |
| Mean | - | - | - | 2.07 | 2.08 | 0.02 | - | - | - | 2.17 | 2.18 | 0.01 | 2.2 | 2.15 | 0.04 | 2.11 | 2.12 | 0.01 | 2.13 | 2.14 | 0.00 | 1.69 | 1.67 | 0.02 | 2.18 | 2.19 | 0.02 | 2.68 | 2.68 | - | 1.18 | 1.18 | 0.00 | 2.34 | 2.35 | 0.02 |
| SD | - | - | - | 0.74 | 0.75 | - | - | - | - | 0.71 | 0.68 | - | 1.15 | 1.13 | - | 0.68 | 0.67 | - | 0.83 | 0.8 | - | 0.97 | 0.95 | - | 0.71 | 0.71 | - | 1.1 | 1.04 | - | 0.48 | 0.43 | - | 0.72 | 0.72 | - |
| Median | - | - | - | 2 | 2 | - | - | - | - | 2 | 2 | - | 2 | 2 | - | 2 | 2 | - | 2 | 2 | - | 2 | 2 | - | 2 | 2 | - | 3 | 3 | - | 1 | 1 | - | 2 | 2 | - |
| Q1 ; Q3 | - | - | - | 2.00 ; 2.00 | 2.00 ; 2.00 | - | - | - | - | 2.00 ; 2.00 | 2.00 ; 3.00 | - | 1.0 ; 3.0 | 1.0 ; 3.0 | - | 2 ; 3 | 2 ; 3 | - | 2.00 ; 3.00 | 2.00 ; 3.00 | - | 1.00 ; 2.00 | 1.00 ; 2.00 | - | 2.00 ; 3.00 | 2.00 ; 3.00 | - | 2.00 ; 3.00 | 2.00 ; 3.00 | - | 1.00 ; 1.00 | 1.00 ; 1.00 | - | 2.00 ; 3.00 | 2.00 ; 3.00 | - |
| Min ; Max | - | - | - | 1.00 ; 6.00 | 1.00 ; 6.00 | - | - | - | - | 1.00 ; 6.00 | 1.00 ; 5.00 | - | 1.0 ; 7.0 | 1.0 ; 8.0 | - | 1 ; 5 | 1 ; 5 | - | 1.00 ; 7.00 | 1.00 ; 6.00 | - | 0.00 ; 6.00 | 0.00 ; 6.00 | - | 1.00 ; 6.00 | 1.00 ; 5.00 | - | 0.00 ; 10.00 | 0.00 ; 10.00 | - | 1.00 ; 6.00 | 1.00 ; 4.00 | - | 1.00 ; 4.00 | 1.00 ; 4.00 | - |
| **Diabetes medication use** | | | | | | | | | | | | | | | | | | | | | | | | | | | | | | | | | | | | |
| Naive new use of antidiabetic drugs | - | - | - | 117 (1.99) | 125 (2.13) | 0.01 | - | - | - | 377 (13.18) | 383 (13.39) | 0.01 | 1194 (34.74) | 1246 (36.25) | 0.03 | 1619 (28.76) | 1625 (28.87) | 0.00 | 122 (3.29) | 116 (3.13) | 0.01 | 268 (6.23) | 242 (5.62) | 0.03 | 17 (0.23) | 13 (0.18) | 0.01 | 0 (0.00) | 0 (0.00) | 0.00 | 7 (1.47) | 7 (1.47) | 0.00 | 22 (1.93) | 17 (1.49) | 0.03 |
| Initiation of the study drug (empagliflozin/any SGLT-2 inhibitor/any DPP-4 inhibitor) as monotherapy | - | - | - | 53 (0.90) | 55 (0.94) | 0.00 | - | - | - | 122 (4.26) | 121 (4.23) | 0.00 | 813 (23.65) | 826 (24.03) | 0.01 | 428 (7.60) | 440 (7.82) | 0.01 | 91 (2.45) | 86 (2.32) | 0.01 | 2055 (47.74) | 2109 (48.99) | 0.03 | 9 (0.12) | 7 (0.10) | 0.01 | 1060 (10.44) | 1062 (10.46) | 0.00 | 474 (99.58) | 474 (99.58) | 0.00 | 16 (1.40) | 13 (1.14) | 0.02 |
| Dual therapy with metformin (without the use of other antidiabetic drugs) | - | - | - | <5 | <5 | 0.00 | - | - | - | 1342 (46.91) | 1351 (47.22) | 0.01 | 412 (11.99) | 453 (13.18) | 0.04 | 528 (9.38) | 510 (9.06) | 0.01 | 199 (5.36) | 207 (5.58) | 0.01 | - | - | - | 363 (5.01) | 382 (5.28) | 0.01 | 3247 (31.98) | 3242 (31.93) | 0.00 | 7 (1.47) | 6 (1.26) | 0.02 | 478 (41.97) | 466 (40.91) | 0.02 |
| Sulfonylureas 2nd generation (concomitant initiation or current use) | - | - | - | 12 (0.20) | 15 (0.26) | 0.01 | - | - | - | 282 (9.86) | 294 (10.28) | 0.01 | - | - | - | 1582 (28.10) | 1623 (28.83) | 0.02 | 553 (14.90) | 581 (15.66) | 0.02 | 102 (2.37) | 105 (2.44) | 0.00 | 875 (12.08) | 881 (12.17) | 0.00 | - | - | - | 10 (2.10) | 12 (2.52) | 0.03 | 376 (33.01) | 365 (32.05) | 0.02 |
| Insulin (concomitant initiation or current use) | - | - | - | 174 (2.96) | 189 (3.22) | 0.01 | - | - | - | 456 (15.94) | 444 (15.52) | 0.01 | - | - | - | 444 (7.89) | 486 (8.63) | 0.03 | 319 (8.60) | 319 (8.60) | 0.00 | 1315 (30.55) | 1260 (29.27) | 0.03 | 1605 (22.17) | 1670 (23.06) | 0.02 | - | - | - | 5 (1.05) | 6 (1.26) | 0.02 | 104 (9.13) | 125 (10.97) | 0.06 |

**CABG** - coronary artery bypass grafting; **CHF** - congestive heart failure; **DPP-4i -** dipeptidyl peptidase-4 inhibitor; **HbA1c** - Glycated hemoglobin; **Max** - maximum; **Min** - minimum; **PTCA** - percutaneous transluminal coronary angioplasty; **Q1** - 1st quartile; **Q3** - 3rd quartile; **SD** - standard deviation; **SGLT2i** - sodium-glucose cotransporter-2 inhibitor; **Std Diff** - standardized difference.

The combined comorbidity score is a single numeric comorbidity score for predicting short and long-term mortality, by combining conditions in the Charlson and Elixhauser comorbidity measures. Presence of diagnosis codes indicating existence of certain conditions may themselves be indicators for other factors that are inversely associated with 1-year mortality or may reflect idiosyncrasies of administrative data which is why negative values can occur. However, the underlying diagnoses should not be regarded as having a preventive/protective effect. JJ Gagne et al. *A combined comorbidity score predicted mortality in elderly patients better than existing scores.* J Clin Epidemiol. 2011 July ; 64(7): 749–759. doi:10.1016/j.jclinepi.2010.10.004.

Lookback period for combined comorbidity score is ever before index date for Denmark, Finland, Norway, Sweden and UK THIN and 12 months prior to the index date for other countries.

Lookback period for comorbidities and diabetes medication use is ever before index date for Denmark, Finland, Norway, Sweden and UK THIN, 6 months prior to the index date for Germany and 12 months or ever before for UK CPRD depending on covariate. For other countries, it is 12 months prior to the index date.

**Table S5b. Baseline characteristics in the empagliflozin and DPP-4i sub-cohorts after PS matching among patients with pre-existing cardiovascular disease**

| **Variable** | **Denmark** | | | **Finland** | | | **Germany** | | | **Israel** | | | **Japan** | | | **South Korea** | | | **Norway** | | | **Spain** | | | **Sweden** | | | **Taiwan** | | | **UK CPRD** | | | **UK THIN** | | |
| --- | --- | --- | --- | --- | --- | --- | --- | --- | --- | --- | --- | --- | --- | --- | --- | --- | --- | --- | --- | --- | --- | --- | --- | --- | --- | --- | --- | --- | --- | --- | --- | --- | --- | --- | --- | --- |
|  | **EMPA** | **DPP4i** | **Std Diff** | **EMPA** | **DPP4i** | **Std Diff** | **EMPA** | **DPP4i** | **Std Diff** | **EMPA** | **DPP4i** | **Std Diff** | **EMPA** | **DPP4i** | **Std Diff** | **EMPA** | **DPP4i** | **Std Diff** | **EMPA** | **DPP4i** | **Std Diff** | **EMPA** | **DPP4i** | **Std Diff** | **EMPA** | **DPP4i** | **Std Diff** | **EMPA** | **DPP4i** | **Std Diff** | **EMPA** | **DPP4i** | **Std Diff** | **EMPA** | **DPP4i** | **Std Diff** |
| **Number of patients** | 3859 | 3859 |  | 5819 | 5819 |  | 166 | 166 |  | 983 | 983 |  | 2152 | 5152 |  | 3444 | 3444 |  | 2559 | 2559 |  | 1536 | 1536 |  | 8429 | 8429 |  | 3889 | 3889 |  | 229 | 229 |  | 127 | 127 |  |
| **Age at index** | | | | | | | | | | | | | | | | | | | | | | | | | | | | | | | | | | | | |
| Mean | - | - | - | 65.62 | 65.6 | 0.00 | 66.79 | 66.38 | 0.04 | 66.24 | 66.9 | 0.07 | 64.54 | 64.72 | 0.01 | 61.27 | 61.15 | 0.01 | 64.83 | 64.85 | 0.00 | 68.57 | 68.48 | 0.01 | 66.57 | 66.3 | 0.03 | 61.67 | 61.5 | 0.01 | 61.59 | 61.31 | 0.03 | 65.9 | 66.87 | 0.10 |
| SD | - | - | - | 10.07 | 10.81 | - | 9.58 | 9.78 | - | 9.89 | 10.24 | - | 12.29 | 12.58 | - | 11.3 | 11.56 | - | 10.45 | 11.21 | - | 10.09 | 10.9 | - | 9.57 | 9.95 | - | 11.88 | 12.31 | - | 10.71 | 10.67 | - | 9.02 | 9.91 | - |
| Median | - | - | - | 66 | 67 | - | 68.39 | 67.67 | - | 66.87 | 67.19 | - | 66 | 66 | - | 61 | 61 | - | 66 | 66 | - | 69 | 69 | - | 68 | 67 | - | 62.15 | 61.53 | - | 62.65 | 61.35 | - | 67 | 67 | - |
| Q1 ; Q3 | - | - | - | 59.00 ; 72.00 | 59.00 ; 72.00 | - | 59.71 ; 73.82 | 59.08 ; 73.07 | - | 60.01 ; 73.02 | 60.97 ;   73.44 | - | 56.0 ; 73.0 | 56.0 ; 74.0 | - | 54 ; 69 | 54 ; 70 | - | 57.00 ; 72.00 | 58.00 ; 72.00 | - | 62.00 ; 76.00 | 61.00 ; 76.00 | - | 61.00 ; 73.00 | 60.00 ; 73.00 | - | 54.05 ; 69.20 | 53.22 ; 69.29 | - | 53.88 ; 69.80 | 54.27 ; 69.63 | - | 59.00 ; 72.00 | 60.00 ; 74.00 | - |
| Min ; Max | - | - | - | 27.00 ; 99.00 | 19.00 ; 98.00 | - | - | - | - | 33.25 ; 92.81 | 32.94 ;   92.44 | - | 24.0 ; 90.0 | 22.0 ; 90.0 | - | 19 ; 94 | 19 ; 94 | - | 29.00 ; 99.00 | 21.00 ; 95.00 | - | 36.00 ; 93.00 | 22.00 ; 97.00 | - | 24.00 ; 95.00 | 19.00 ; 96.00 | - | 20.47 ; 95.37 | 19.04 ; 97.31 | - | 32.39 ; 84.69 | 24.99 ; 85.72 | - | 47.34 ; 90.38 | 40.20 ; 90.82 | - |
| 18-54 | - | - | - | 868 (14.92) | 841 (14.45) | - | 21 (12.65) | 22 (13.25) | - | 129 (13.12) | 133 (13.53) | - | 476 (22.12) | 492 (22.86) | - | 907 (26.34) | 947 (27.50) | 0.02 | 447 (17.47) | 439 (17.16) | - | 137 (8.92) | 149 (9.70) | - | 1001 (11.88) | 1020 (12.10) | - | 1070 (27.51) | 1147 (29.49) | - | 66 (28.82) | 64 (27.95) | 0.07 | 17 (13.39) | 16 (12.60) | - |
| 55-64 | - | - | - | 1589 (27.31) | 1598 (27.46) | - | 46 (27.71) | 48 (28.92) | - | 285 (28.99) | 268 (27.26) | - | 504 (23.42) | 450 (20.91) | - | 1195 (34.70) | 1184 (34.38) | - | 748 (29.23) | 754 (29.46) | - | 386 (25.13) | 380 (24.74) | - | 2217 (26.30) | 2225 (26.40) | - | 1283 (32.99) | 1239 (31.86) | - | 69 (30.13) | 75 (32.75) | - | 38 (29.92) | 32 (25.20) | - |
| 65-74 | - | - | - | 2284 (39.25) | 2321 (39.89) | - | 66 (39.76) | 66 (39.76) | - | 378 (38.45) | 385 (39.17) | - | 700 (32.53) | 710 (32.99) | - | 903 (26.22) | 880 (25.55) | - | 895 (34.97) | 898 (35.09) | - | 562 (36.59) | 544 (35.42) | - | 3527 (41.84) | 3595 (42.65) | - | 1009 (25.94) | 950 (24.43) | - | 76 (33.19) | 75 (32.75) | - | 50 (39.37) | 48 (37.80) | - |
| 75+ | - | - | - | 1078 (18.53) | 1059 (18.20) | - | 33 (19.88) | 30 (18.07) | - | 191 (19.43) | 197 (20.04) | - | 472 (21.93) | 500 (23.23) | - | 439 (12.75) | 433 (12.57) | - | 469 (18.33) | 468 (18.29) | - | 451 (29.36) | 463 (30.14) | - | 1684 (19.98) | 1589 (18.85) | - | 527 (13.55) | 553 (14.22) | - | 18 (7.86) | 15 (6.55) | - | 22 (17.32) | 31 (24.41) | - |
| **Sex** | | | | | | | | | | | | | | | | | | | | | | | | | | | | | | | | | | | | |
| Female | - | - | - | 2402 (41.28) | 2384 (40.97) | 0.01 | 44 (26.51) | 40 (24.10) | 0.05 | 276 (28.08) | 272 (27.67) | 0.01 | 545 (25.33) | 571 (26.53) | 0.03 | 1474 (42.80) | 1428 (41.46) | 0.03 | 807 (31.54) | 826 (32.28) | 0.02 | 621 (40.43) | 625 (40.69) | 0.01 | 2781 (32.99) | 2712 (32.17) | 0.02 | 1435 (36.90) | 1478 (38.00) | 0.02 | 82 (35.81) | 90 (39.30) | 0.07 | 33 (25.98) | 32 (25.20) | 0.02 |
| Male | - | - | - | 3417 (58.72) | 3435 (59.03) | - | 122 (73.49) | 126 (75.90) | - | 707 (71.92) | 711 (72.33) | - | 1607 (74.67) | 1581 (73.47) | - | 1970 (57.20) | 2016 (58.54) | - | 1752 (68.46) | 1733 (67.72) | - | 915 (59.57) | 911 (59.31) | - | 5648 (67.01) | 5717 (67.83) | - | 2454 (63.10) | 2411 (62.00) | - | 147 (64.19) | 139 (60.70) | - | 94 (74.02) | 95 (74.80) | - |
| **HbA1c (%)** | | | | | | | | | | | | | | | | | | | | | | | | | | | | | | | | | | | | |
| Mean | - | - | - | 7.40 | 7.29 | 0.07 | 7.94 | 7.99 | 0.04 | 7.85 | 7.86 | 0.00 | 7.65 | 7.64 | 0.01 | - | - | - | - | - | - | 7.28 | 7.15 | 0.09 | 8.06 | 8.08 | 0.02 | - | - | - | 9.2 | 9.37 | 0.10 | 9.15 | 9.14 | 0.00 |
| SD | - | - | - | 3.81 | 3.76 | - | 1.68 | 1.56 | - | 1.51 | 1.54 | - | 1.65 | 1.74 | - | - | - | - | - | - | - | 1.41 | 1.27 | - | 3.53 | 3.57 | - | - | - | - | 1.6 | 1.76 | - | 1.46 | 1.7 | - |
| Median | - | - | - | 6.91 | 6.82 | - | 7.63 | 7.6 | - | 7.6 | 7.6 | - | 7.11 | 7.1 | - | - | - | - | - | - | - | 6.9 | 6.9 | - | 7.82 | 7.82 | - | - | - | - | 9.01 | 8.92 | - | 9 | 8 | - |
| Q1 ; Q3 | - | - | - | 6.27 ; 8.01 | 6.18 ; 7.92 | - | 6.88 ; 8.59 | 6.81 ; 9.01 | - | 6.80 ; 8.50 | 6.80 ; 8.50 | - | 6.7 ; 8.17 | 6.64 ; 8.03 | - | - | - | - | - | - | - | 6.40 ; 7.90 | 6.30 ; 7.60 | - | 7.09 ; 8.74 | 7.09 ; 8.74 | - | - | - | - | 8.01 ; 10.02 | 8.10 ; 10.48 | - | 8.00 ; 9.00 | 7.00 ; 10.00 | - |
| Min ; Max | - | - | - | 4.62 ; 17.52 | 3.89 ; 16.97 | - | - | - | - | 4.70 ; 16.20 | 4.80 ;   18.70 | - | 5.3 ; 17.1 | 5.41 ; 17.0 | - | - | - | - | - | - | - | 5.00 ; 16.40 | 5.00 ; 14.40 | - | 5.08 ; 16.24 | 4.80 ; 17.52 | - | - | - | - | 3.20 ; 13.68 | 5.99 ; 15.87 | - | 5.90 ; 14.69 | 5.72 ; 14.14 | - |
| Missing | - | - | - | 3211 (55.18) | 3139 (53.94) | - | - | - | - | 15 | 13 | - | 1926 (89.50) | 1972 (91.64) | - | - | - | - | - | - | - | 790 (51.40) | 841 (54.80) | - | - | - | - | - | - | - | - | - | - | - | - | - |
| **Total cholesterol (mg/dl)** | | | | | | | | | | | | | | | | | | | | | | | | | | | | | | | | | | | | |
| Mean | - | - | - | 172.55 | 171.52 | 0.02 | 167.36 | 177.6 | - | 156.97 | 154.83 | 0.05 | 180.63 | 183.33 | 0.07 | - | - | - | - | - | - | 174.88 | 175.66 | 0.02 | 168.94 | 168.7 | 0.00 | - | - | - | 167.6 | 165.31 | 0.06 | 159.2 | 154.3 | 0.12 |
| SD | - | - | - | 48.21 | 45.32 | - | 38.67 | 49.64 | - | 44.27 | 40.21 | - | 40.27 | 40.62 | - | - | - | - | - | - | - | 39.57 | 39.89 | - | 44.78 | 44.73 | - | - | - | - | 40.49 | 39.95 | - | 45.95 | 38.16 | - |
| Median | - | - | - | 166.28 | 166.28 | - | 163 | 171.5 | - | 149 | 149 | - | 176.2 | 179 | - | - | - | - | - | - | - | 170 | 173 | - | 162.41 | 162.41 | - | - | - | - | 162.41 | 162.41 | - | 150 | 150 | - |
| Q1 ; Q3 | - | - | - | 139.21 ; 201.08 | 139.21 ; 197.22 | - | 144.00 ; 188.00 | 144.00 ; 203.50 | - | 128.00 ;   174.00 | 127.00 ;   174.00 | - | 154.33 ; 202.0 | 157.83 ; 205.33 | - | - | - | - | - | - | - | 146.00 ; 199.00 | 147.00 ; 201.00 | - | 139.21 ; 193.35 | 135.35 ; 193.35 | - | - | - | - | 139.21 ; 185.61 | 135.34 ; 185.61 | - | 127.00 ; 181.00 | 127.00 ; 177.00 | - |
| Min ; Max | - | - | - | 61.87 ; 858.47 | 73.47 ; 576.18 | - | - | - | - | 65.00 ; 461.00 | 72.00 ;   360.00 | - | 84.14 ; 329.0 | 93.0 ; 377.0 | - | - | - | - | - | - | - | 73.00 ; 324.00 | 74.00 ; 323.00 | - | 46.40 ; 464.04 | 54.14 ; 429.24 | - | - | - | - | 73.47 ; 313.23 | 88.94 ; 340.29 | - | 77.34 ; 324.83 | 88.94 ; 270.69 | - |
| Missing | - | - | - | 3109 (53.43) | 3058 (52.55) | - | 45 (27.11) | 54 (32.53) | - | 18 | 18 | - | 1950 (90.61) | 1995 (92.70) | - | - | - | - | - | - | - | 665 (43.30) | 717 (46.70) | - | - | - | - | - | - | - | - | - | - | - | - | - |
| **Low-density lipoprotein level (mg/dl)** | | | | | | | | | | | | | | | | | | | | | | | | | | | | | | | | | | | | |
| Mean | - | - | - | 100.41 | 99.82 | 0.02 | 99.13 | 106.94 | - | 80.74 | 79.13 | 0.05 | 110.14 | 108.96 | 0.04 | - | - | - | - | - | - | 97.83 | 96.83 | 0.03 | 91.59 | 91.57 | 0.00 | - | - | - | 109.3 | 105.98 | 0.08 | 85.77 | 79.07 | 0.17 |
| SD | - | - | - | 38.73 | 37.88 | - | 35.1 | 40.29 | - | 33.43 | 31.03 | - | 32.24 | 35.54 | - | - | - | - | - | - | - | 33.13 | 32.44 | - | 37.69 | 37.57 | - | - | - | - | 41.53 | 40.27 | - | 43.29 | 33.78 | - |
| Median | - | - | - | 92.81 | 92.81 | - | 93 | 99 | - | 73.9 | 73 | - | 109 | 106.7 | - | - | - | - | - | - | - | 92.8 | 94 | - | 84.69 | 85.07 | - | - | - | - | 104.41 | 100.54 | - | 73 | 73 | - |
| Q1 ; Q3 | - | - | - | 73.47 ; 123.74 | 73.47 ; 123.74 | - | 75.00 ; 121.00 | 80.00 ; 129.25 | - | 58.80 ; 94.70 | 57.90 ;   93.20 | - | 88.52 ; 126.75 | 85.7 ; 131.0 | - | - | - | - | - | - | - | 74.00 ; 118.00 | 73.00 ; 117.20 | - | 65.74 ; 112.14 | 64.58 ; 112.14 | - | - | - | - | 81.21 ; 134.18 | 79.27 ; 130.60 | - | 54.00 ; 108.00 | 58.00 ; 92.00 | - |
| Min ; Max | - | - | - | 7.73 ; 309.36 | 11.60 ; 297.76 | - | - | - | - | 16.60 ; 253.20 | 10.10 ;   221.10 | - | 48.0 ; 224.0 | 25.0 ; 267.0 | - | - | - | - | - | - | - | 22.00 ; 217.00 | 0.00 ; 210.00 | - | 8.89 ; 320.96 | 8.89 ; 363.50 | - | - | - | - | 27.07 ; 282.29 | 27.07 ; 259.09 | - | 15.47 ; 286.16 | 19.33 ; 224.28 | - |
| Missing | - | - | - | 3170 (54.48) | 3105 (53.36) | - | 58 (34.94) | 66 (39.76) | - | 60 | 80 | - | 1966 (91.36) | 1998 (92.84) | - | - | - | - | - | - | - | 639 (41.60) | 706 (46.00) | - | - | - | - | - | - | - | - | - | - | - | - | - |
| **High-density lipoprotein level (mg/dl)** | | | | | | | | | | | | | | | | | | | | | | | | | | | | | | | | | | | | |
| Mean | - | - | - | 46.59 | 46.67 | 0.01 | 47.44 | 43.28 | - | 41.22 | 41.02 | 0.02 | 46.39 | 49.8 | 0.23 | - | - | - | - | - | - | 45.5 | 45.29 | 0.02 | 44.31 | 43.98 | 0.02 | - | - | - | 43.16 | 43.35 | 0.02 | 40.65 | 41.17 | 0.05 |
| SD | - | - | - | 13.49 | 13.92 | - | 30.55 | 11.15 | - | 9.99 | 10.29 | - | 14.49 | 15.18 | - | - | - | - | - | - | - | 11.98 | 12.25 | - | 12.71 | 12.58 | - | - | - | - | 10.68 | 11.75 | - | 10.14 | 11.45 | - |
| Median | - | - | - | 44.47 | 44.86 | - | 42 | 42 | - | 40 | 40 | - | 44 | 46.5 | - | - | - | - | - | - | - | 44 | 43 | - | 42.54 | 42.54 | - | - | - | - | 42.54 | 42.54 | - | 38 | 38 | - |
| Q1 ; Q3 | - | - | - | 37.32 ; 53.75 | 37.12 ; 53.75 | - | 34.00 ; 50.00 | 35.00 ; 51.00 | - | 34.00 ; 46.00 | 34.00 ;   46.00 | - | 38.12 ; 51.7 | 40.25 ; 56.3 | - | - | - | - | - | - | - | 37.00 ; 52.00 | 36.10 ; 52.00 | - | 34.80 ; 50.27 | 34.80 ; 50.27 | - | - | - | - | 35.58 ; 49.50 | 34.80 ; 49.11 | - | 34.00 ; 46.00 | 34.00 ; 46.00 | - |
| Min ; Max | - | - | - | 12.76 ; 114.85 | 9.28 ; 190.26 | - | - | - | - | 11.00 ; 96.00 | 13.00 ;   90.00 | - | 17.33 ; 154.0 | 16.0 ; 128.0 | - | - | - | - | - | - | - | 21.00 ; 101.00 | 21.00 ; 96.60 | - | 7.73 ; 158.55 | 11.60 ; 154.68 | - | - | - | - | 19.33 ; 85.07 | 23.20 ; 116.78 | - | 19.33 ; 73.47 | 15.47 ; 75.41 | - |
| Missing | - | - | - | 3115 (53.53) | 3069 (52.74) | - | 57 (34.34) | 63 (37.95) | - | 18 | 19 | - | 1929 (89.64) | 1967 (91.40) | - | - | - | - | - | - | - | 621 (40.40) | 700 (45.60) | - | - | - | - | - | - | - | - | - | - | - | - | - |
| **Triglyceride level (mg/dl)** | | | | | | | | | | | | | | | | | | | | | | | | | | | | | | | | | | | | |
| Mean | - | - | - | 182.78 | 184.76 | 0.01 | 193.78 | 200.15 | - | 176.54 | 179.56 | 0.03 | 177.65 | 162.73 | 0.13 | - | - | - | - | - | - | 150.03 | 156.7 | 0.09 | 189.49 | 189.85 | 0.00 | - | - | - | 210.74 | 211.58 | 0.00 | 209.55 | 220.31 | 0.08 |
| SD | - | - | - | 127.54 | 152.31 | - | 113.31 | 102.67 | - | 113.62 | 117.11 | - | 136.02 | 97.62 | - | - | - | - | - | - | - | 68.94 | 72.42 | - | 135.29 | 140.44 | - | - | - | - | 237.15 | 168.58 | - | 137.44 | 132.94 | - |
| Median | - | - | - | 151.45 | 152.34 | - | 164 | 173 | - | 154 | 152 | - | 142.5 | 143.25 | - | - | - | - | - | - | - | 134 | 144 | - | 159.43 | 159.43 | - | - | - | - | 177.15 | 159.43 | - | 168 | 186 | - |
| Q1 ; Q3 | - | - | - | 112.48 ; 212.57 | 112.48 ; 216.11 | - | 129.00 ; 220.50 | 123.00 ; 256.50 | - | 108.00 ;   217.00 | 108.00 ;   214.00 | - | 102.93 ; 200.69 | 97.0 ; 190.0 | - | - | - | - | - | - | - | 101.00 ; 184.00 | 100.00 ; 197.20 | - | 115.14 ; 221.43 | 115.14 ; 221.43 | - | - | - | - | 115.15 ; 248.01 | 119.57 ; 256.86 | - | 124.00 ; 248.00 | 124.00 ; 288.00 | - |
| Min ; Max | - | - | - | 35.43 ; 1879.46 | 26.57 ; 3657.06 | - | - | - | - | 41.00 ;   1644.00 | 43.00 ;   1745.00 | - | 43.0 ; 1117.8 | 29.0 ; 703.5 | - | - | - | - | - | - | - | 43.00 ; 397.00 | 43.00 ; 399.00 | - | 35.43 ; 2648.24 | 26.57 ; 2922.81 | - | - | - | - | 0.00 ; 3348.10 | 44.29 ; 1302.04 | - | 57.57 ; 956.60 | 53.14 ; 806.02 | - |
| Missing | - | - | - | 3107 (53.39) | 3063 (52.64) | - | 49 (29.52) | 57 (34.34) | - | 18 | 19 | - | 1930 (89.68) | 1970 (91.54) | - | - | - | - | - | - | - | 599 (39.00) | 679 (44.20) | - | - | - | - | - | - | - | - | - | - | - | - | - |
| **Estimated Glomerular Filtration Rate (mL/min/1,73m^2^)** | | | | | | | | | | | | | | | | | | | | | | | | | | | | | | | | | | | | |
| Mean | - | - | - | 83.88 | 79.89 | 0.20 | 73.21 | 73.08 | - | 80.53 | 80.68 | 0.01 | - | - | - | - | - | - | - | - | - | 80.6 | 77.31 | 0.17 | 82.69 | 82.88 | 0.02 | - | - | - | 82.62 | 83.14 | 0.03 | 80.94 | 80.16 | 0.05 |
| SD | - | - | - | 17.09 | 21.88 | - | 20.47 | 20.68 | - | 19.08 | 20.89 | - | - | - | - | - | - | - | - | - | - | 18.15 | 20.65 | - | 17.16 | 18.98 | - | - | - | - | 17.37 | 21.84 | - | 16.9 | 16.68 | - |
| Median | - | - | - | 86.11 | 83.74 | - | 71.36 | 74.69 | - | 83.08 | 86 | - | - | - | - | - | - | - | - | - | - | 84.29 | 81.16 | - | 85.17 | 86.52 | - | - | - | - | 83 | 85 | - | 81 | 82 | - |
| Q1 ; Q3 | - | - | - | 71.81 ; 95.97 | 64.34 ; 96.31 | - | 58.81 ; 88.09 | 58.96 ; 88.06 | - | 66.10 ; 95.66 | 66.57 ;   95.82 | - | - | - | - | - | - | - | - | - | - | 68.88 ; 93.77 | 62.18 ; 92.97 | - | 71.37 ; 94.65 | 69.76 ; 96.57 | - | - | - | - | 71.00 ; 95.79 | 72.00 ; 98.28 | - | 67.00 ; 94.00 | 68.00 ; 92.00 | - |
| Min ; Max | - | - | - | 19.34 ; 143.44 | 10.56 ; 145.13 | - | - | - | - | 27.13 ; 126.57 | 16.55 ;   125.60 | - | - | - | - | - | - | - | - | - | - | 19.93 ; 135.83 | 18.94 ; 128.57 | - | 21.48 ; 216.17 | 20.80 ; 201.51 | - | - | - | - | 39.00 ; 123.69 | 18.00 ; 163.95 | - | 39.39 ; 130.70 | 37.51 ; 112.90 | - |
| Missing | - | - | - | 2781 (47.79) | 2701 (46.42) | - | 29 (17.47) | 42 (25.30) | - | 13 | 14 | - | - | - | - | - | - | - | - | - | - | 719 (46.80) | 740 (48.20) | - | - | - | - | - | - | - | - | - | - | - | - | - |
| **Creatinine (mg/dl)** | | | | | | | | | | | | | | | | | | | | | | | | | | | | | | | | | | | | |
| Mean | - | - | - | 0.86 | 0.94 | 0.27 | 1.07 | 1.07 | - | 0.93 | 0.93 | 0.03 | 1.02 | 1.11 | 0.09 | - | - | - | - | - | - | 0.88 | 0.94 | 0.21 | 0.88 | 0.89 | 0.01 | - | - | - | 0.89 | 0.88 | 0.05 | - | - | - |
| SD | - | - | - | 0.22 | 0.31 | - | 0.68 | 0.38 | - | 0.25 | 0.32 | - | 0.54 | 1.29 | - | - | - | - | - | - | - | 0.26 | 0.3 | - | 0.23 | 0.25 | - | - | - | - | 0.22 | 0.28 | - | - | - | - |
| Median | - | - | - | 0.84 | 0.87 | - | 1 | 1 | - | 0.89 | 0.86 | - | 0.9 | 0.86 | - | - | - | - | - | - | - | 0.84 | 0.89 | - | 0.86 | 0.85 | - | - | - | - | 0.86 | 0.83 | - | - | - | - |
| Q1 ; Q3 | - | - | - | 0.72 ; 0.97 | 0.73 ; 1.06 | - | 0.81 ; 1.14 | 0.81 ; 1.20 | - | 0.75 ; 1.06 | 0.73 ; 1.06 | - | 0.72 ; 1.11 | 0.73 ; 1.08 | - | - | - | - | - | - | - | 0.70 ; 1.01 | 0.74 ; 1.09 | - | 0.73 ; 0.99 | 0.72 ; 1.02 | - | - | - | - | 0.72 ; 1.02 | 0.71 ; 0.95 | - | - | - | - |
| Min ; Max | - | - | - | 0.36 ; 3.27 | 0.29 ; 3.97 | - | - | - | - | 0.37 ; 2.07 | 0.35 ; 3.23 | - | 0.46 ; 4.14 | 0.42 ; 11.94 | - | - | - | - | - | - | - | 0.30 ; 2.51 | 0.37 ; 3.19 | - | 0.12 ; 2.53 | 0.12 ; 2.68 | - | - | - | - | 0.46 ; 1.73 | 0.37 ; 2.04 | - | - | - | - |
| Missing | - | - | - | 2777 (47.72) | 2700 (46.40) | - | 29 (17.47) | 42 (25.30) | - | 13 | 14 | - | 1918 (89.13) | 1958 (90.99) | - | - | - | - | - | - | - | 719 (46.80) | 740 (48.20) | - | - | - | - | - | - | - | - | - | - | - | - | - |
| **Combined comorbidity score** | | | | | | | | | | | | | | | | | | | | | | | | | | | | | | | | | | | | |
| Mean | - | - | - | - | - | - | - | - | - | - | - | - | 2.28 | 2.22 | 0.03 | 0.58 | 0.5 | 0.07 | - | - | - | 1.21 | 1.17 | 0.02 | - | - | - | 1.22 | 1.24 | 0.01 | 0.45 | 0.46 | 0.02 | - | - | - |
| SD | - | - | - | - | - | - | - | - | - | - | - | - | 2.02 | 1.96 | - | 1.15 | 1.14 | - | - | - | - | 2.57 | 2.52 | - | - | - | - | 1.64 | 1.74 | - | 1.12 | 1.13 | - | - | - | - |
| Median | - | - | - | - | - | - | - | - | - | - | - | - | 2 | 2 | - | 0 | 0 | - | - | - | - | 0 | 0 | - | - | - | - | 1 | 1 | - | 0 | 0 | - | - | - | - |
| Q1 ; Q3 | - | - | - | - | - | - | - | - | - | - | - | - | 1.0 ; 3.0 | 1.0 ; 3.0 | - | 0 ; 1 | 0 ; 1 | - | - | - | - | 0.00 ; 0.00 | 0.00 ; 0.00 | - | - | - | - | 0.00 ; 2.00 | 0.00 ; 2.00 | - | 0.00 ; 0.00 | 0.00 ; 0.00 | - | - | - | - |
| Min ; Max | - | - | - | - | - | - | - | - | - | - | - | - | -1.0 ; 13.0 | -1.0 ; 11.0 | - | -1 ; 7 | -1 ; 7 | - | - | - | - | -1.00 ; 13.00 | -1.00 ; 15.00 | - | - | - | - | -1.00 ; 9.00 | -2.00 ; 11.00 | - | 0.00 ; 9.00 | 0.00 ; 7.00 | - | - | - | - |
| Missing | - | - | - | - | - | - | - | - | - | - | - | - | - | - | - | - | - | - | - | - | - | 0.00 (0.00) | 0.00 (0.00) | - | - | - | - | - | - | - | - | - | - | - | - | - |
| **Comorbidities and other medical conditions** | | | | | | | | | | | | | | | | | | | | | | | | | | | | | | | | | | | | |
| Diabetic retinopathy | - | - | - | 274 (4.71) | 297 (5.10) | 0.02 | 5 (3.01) | 6 (3.61) | 0.03 | 174 (17.70) | 185 (18.82) | 0.03 | 244 (11.34) | 221 (10.27) | 0.03 | 870 (25.26) | 888 (25.78) | 0.01 | 286 (11.18) | 307 (12.00) | 0.03 | 37 (2.41) | 35 (2.28) | 0.01 | 1005 (11.92) | 997 (11.83) | 0.00 | 366 (9.41) | 339 (8.72) | 0.02 | 0 (0.00) | 0 (0.00) | - | 21 (16.54) | 18 (14.17) | 0.06 |
| Diabetic neuropathy | - | - | - | 95 (1.63) | 97 (1.67) | 0.00 | 138 (83.13) | 124 (74.70) | - | 213 (21.67) | 212 (21.57) | 0.00 | 52 (2.42) | 42 (1.95) | 0.03 | 801 (23.26) | 767 (22.27) | 0.02 | 59 (2.31) | 54 (2.11) | 0.01 | 37 (2.41) | 41 (2.67) | 0.02 | 152 (1.80) | 160 (1.90) | 0.01 | 273 (7.02) | 287 (7.38) | 0.01 | <5 | <5 | 0.00 | 5 (3.94) | 8 (6.30) | 0.11 |
| Diabetic nephropathy | - | - | - | 83 (1.43) | 83 (1.43) | 0.00 | 12 (7.23) | 12 (7.23) | 0.00 | 108 (10.99) | 102 (10.38) | 0.02 | 158 (7.34) | 141 (6.55) | 0.03 | 557 (16.17) | 557 (16.17) | 0.00 | 38 (1.48) | 41 (1.60) | 0.01 | 30 (1.95) | 33 (2.15) | 0.01 | 121 (1.44) | 130 (1.54) | 0.01 | 676 (17.38) | 668 (17.18) | 0.01 | 0 (0.00) | 0 (0.00) | - | 0 (0.00) | 0 (0.00) | - |
| Diabetes with peripheral circulatory disorders | - | - | - | 120 (2.06) | 126 (2.17) | 0.01 | - | - | - | 52 (5.29) | 58 (5.90) | 0.03 | 28 (1.30) | 28 (1.30) | 0.00 | 42 (1.22) | 42 (1.22) | 0.00 | 40 (1.56) | 33 (1.29) | 0.02 | 77 (5.01) | 81 (5.27) | 0.01 | 125 (1.48) | 124 (1.47) | 0.00 | 67 (1.72) | 70 (1.80) | 0.01 | 0 (0.00) | 0 (0.00) | - | 0 (0.00) | 0 (0.00) | - |
| Diabetic foot | - | - | - | 0 (0.00) | 0 (0.00) | - | 51 (30.72) | 46 (27.71) | - | 11 (1.12) | 8 (0.81) | 0.03 | 26 (1.21) | 29 (1.35) | 0.01 | - | - | - | - | - | - | 42 (2.73) | 34 (2.21) | 0.03 | 91 (1.08) | 93 (1.10) | 0.00 | 7 (0.18) | <5 | 0.03 | 0 (0.00) | 0 (0.00) | - | 97 (76.38) | 99 (77.95) | 0.04 |
| Hypoglycemia | - | - | - | 22 (0.38) | 25 (0.43) | 0.01 | <5 | <5 | - | 36 (3.66) | 37 (3.76) | 0.01 | 16 (0.74) | 14 (0.65) | 0.01 | 61 (1.77) | 54 (1.57) | 0.02 | 8 (0.31) | 13 (0.51) | 0.03 | 36 (2.34) | 30 (1.95) | 0.03 | 81 (0.96) | 70 (0.83) | 0.01 | 25 (0.64) | 31 (0.80) | 0.02 | 0 (0.00) | 0 (0.00) | - | <5 | 0 (0.00) | 0.12 |
| Hyperlipidemia | - | - | - | 1504 (25.85) | 1525 (26.21) | 0.01 | 136 (81.93) | 133 (80.12) | - | 319 (32.45) | 339 (34.49) | 0.04 | 1591 (73.93) | 1587 (73.75) | 0.00 | 3236 (93.96) | 3253 (94.45) | 0.02 | 460 (17.98) | 471 (18.41) | 0.01 | 558 (36.33) | 558 (36.33) | 0.00 | 2269 (26.92) | 2255 (26.75) | 0.00 | 2367 (60.86) | 2393 (61.53) | 0.01 | 71 (31.00) | 69 (30.13) | 0.02 | 0 (0.00) | 0 (0.00) | - |
| Ischemic heart disease | - | - | - | 1731 (29.75) | 1749 (30.06) | 0.01 | - | - | - | 532 (54.12) | 538 (54.73) | 0.01 | 1517 (70.49) | 1517 (70.49) | 0.00 | 1804 (52.38) | 1816 (52.73) | 0.01 | 1301 (50.84) | 1282 (50.10) | 0.01 | 483 (31.45) | 467 (30.40) | 0.02 | 3403 (40.37) | 3312 (39.29) | 0.02 | 2804 (72.10) | 2778 (71.43) | 0.01 | 99 (43.23) | 98 (42.79) | 0.01 | 13 (10.24) | 14 (11.02) | 0.02 |
| Stable angina | - | - | - | 685 (11.77) | 690 (11.86) | 0.00 | - | - | - | 41 (4.17) | 46 (4.68) | 0.02 | 925 (42.98) | 902 (41.91) | 0.02 | 399 (11.59) | 390 (11.32) | 0.01 | 537 (20.98) | 518 (20.24) | 0.02 | 385 (25.07) | 369 (24.02) | 0.02 | 1650 (19.58) | 1589 (18.85) | 0.02 | 746 (19.18) | 773 (19.88) | 0.02 | 52 (22.71) | 49 (21.40) | 0.03 | <5 | <5 | 0.00 |
| History of CABG or PTCA | - | - | - | 282 (4.85) | 289 (4.97) | 0.01 | 67 (40.36) | 50 (30.12) | - | 132 (13.43) | 153 (15.56) | 0.06 | 17 (0.79) | 18 (0.84) | 0.01 | 355 (10.31) | 342 (9.93) | 0.01 | 114 (4.45) | 109 (4.26) | 0.01 | 16 (1.04) | 15 (0.98) | 0.01 | 1357 (16.10) | 1328 (15.76) | 0.01 | 24 (0.62) | 21 (0.54) | 0.01 | - | - | - | 16 (12.60) | 14 (11.02) | 0.05 |
| Any stroke | - | - | - | 550 (9.45) | 520 (8.94) | 0.02 | 39 (23.49) | 53 (31.93) | - | 170 (17.29) | 155 (15.77) | 0.04 | 278 (12.92) | 231 (10.73) | 0.07 | 501 (14.55) | 495 (14.37) | 0.01 | 232 (9.07) | 233 (9.11) | 0.00 | 112 (7.29) | 103 (6.71) | 0.02 | 872 (10.35) | 873 (10.36) | 0.00 | 210 (5.40) | 210 (5.40) | 0.00 | 28 (12.23) | 28 (12.23) | 0.00 | <5 | <5 | 0.06 |
| CHF | - | - | - | 784 (13.47) | 759 (13.04) | 0.01 | - | - | - | 0 (0.00) | 0 (0.00) | - | 1521 (70.68) | 1512 (70.26) | 0.01 | 671 (19.48) | 651 (18.90) | 0.01 | 350 (13.68) | 354 (13.83) | 0.00 | 148 (9.64) | 149 (9.70) | 0.00 | 1187 (14.08) | 1168 (13.86) | 0.01 | 861 (22.14) | 890 (22.89) | 0.02 | 17 (7.42) | 22 (9.61) | 0.08 | <5 | <5 | 0.00 |
| Peripheral Vascular disease or surgery | - | - | - | 405 (6.96) | 407 (6.99) | 0.00 | - | - | - | 119 (12.11) | 121 (12.31) | 0.01 | 222 (10.32) | 254 (11.80) | 0.05 | 1643 (47.71) | 1625 (47.18) | 0.01 | 1636 (63.93) | 1648 (64.40) | 0.01 | 89 (5.79) | 88 (5.73) | 0.00 | 845 (10.02) | 832 (9.87) | 0.01 | 245 (6.30) | 258 (6.63) | 0.01 | 21 (9.17) | 21 (9.17) | 0.00 | 0 (0.00) | 0 (0.00) | - |
| Hypertension | - | - | - | 2990 (51.38) | 2988 (51.35) | 0.00 | 150 (90.36) | 150 (90.36) | - | 500 (50.86) | 499 (50.76) | 0.00 | 1772 (82.34) | 1738 (80.76) | 0.04 | 2655 (77.09) | 2639 (76.63) | 0.01 | 374 (14.62) | 368 (14.38) | 0.01 | 1012 (65.89) | 1003 (65.30) | 0.01 | 4318 (51.23) | 4284 (50.82) | 0.01 | 2747 (70.64) | 2794 (71.84) | 0.03 | 170 (74.24) | 173 (75.55) | 0.03 | 8 (6.30) | 12 (9.45) | 0.12 |
| Chronic kidney disease | - | - | - | 36 (0.62) | 37 (0.64) | 0.00 | - | - | - | 74 (7.53) | 85 (8.65) | 0.04 | 182 (8.46) | 210 (9.76) | 0.05 | 94 (2.73) | 104 (3.02) | 0.02 | 54 (2.11) | 50 (1.95) | 0.01 | 30 (1.95) | 25 (1.63) | 0.02 | 85 (1.01) | 80 (0.95) | 0.01 | 249 (6.40) | 246 (6.33) | 0.00 | 0 (0.00) | 0 (0.00) | - | 0 (0.00) | 0 (0.00) | - |
| **Number of antidiabetic substances at index date** | | | | | | | | | | | | | | | | | | | | | | | | | | | | | | | | | | | | |
| Mean | - | - | - | 2.14 | 2.16 | 0.02 | - | - | - | 2.25 | 2.24 | 0.01 | 1.82 | 1.7 | 0.12 | 2.09 | 2.09 | 0.00 | 2.18 | 2.2 | 0.02 | 1.73 | 1.73 | 0.00 | 2.11 | 2.14 | 0.03 | 2.46 | 2.45 | - | 1.21 | 1.2 | 0.02 | 2.37 | 2.35 | 0.02 |
| SD | - | - | - | 0.81 | 0.82 | - | - | - | - | 0.71 | 0.71 | - | 1.09 | 0.98 | - | 0.74 | 0.71 | - | 0.88 | 0.86 | - | 1.02 | 0.96 | - | 0.73 | 0.74 | - | 1.1 | 0.99 | - | 0.51 | 0.49 | - | 0.71 | 0.83 | - |
| Median | - | - | - | 2 | 2 | - | - | - | - | 2 | 2 | - | 1 | 1 | - | 2 | 2 | - | 2 | 2 | - | 2 | 2 | - | 2 | 2 | - | 2 | 2 | - | 1 | 1 | - | 2 | 2 | - |
| Q1 ; Q3 | - | - | - | 2.00 ; 2.00 | 2.00 ; 3.00 | - | - | - | - | 2.00 ; 3.00 | 2.00 ; 3.00 | - | 1.0 ; 2.0 | 1.0 ; 2.0 | - | 2 ; 3 | 2 ; 3 | - | 2.00 ; 3.00 | 2.00 ; 3.00 | - | 1.00 ; 2.00 | 1.00 ; 2.00 | - | 2.00 ; 3.00 | 2.00 ; 3.00 | - | 2.00 ; 3.00 | 2.00 ; 3.00 | - | 1.00 ; 1.00 | 1.00 ; 1.00 | - | 2.00 ; 3.00 | 2.00 ; 3.00 | - |
| Min ; Max | - | - | - | 1.00 ; 6.00 | 1.00 ; 6.00 | - | - | - | - | 1.00 ; 5.00 | 1.00 ; 5.00 | - | 1.0 ; 7.0 | 1.0 ; 6.0 | - | 1 ; 5 | 1 ; 4 | - | 1.00 ; 7.00 | 1.00 ; 6.00 | - | 0.00 ; 6.00 | 0.00 ; 5.00 | - | 1.00 ; 6.00 | 1.00 ; 7.00 | - | 1.00 ; 9.00 | 0.00 ; 8.00 | - | 1.00 ; 4.00 | 1.00 ; 4.00 | - | 1.00 ; 4.00 | 1.00 ; 5.00 | - |
| **Diabetes medication use** | | | | | | | | | | | | | | | | | | | | | | | | | | | | | | | | | | | | |
| Naive new use of antidiabetic drugs | - | - | - | 64 (1.10) | 74 (1.27) | 0.02 | - | - | - | 66 (6.71) | 69 (7.02) | 0.01 | 1029 (47.82) | 1150 (53.44) | 0.11 | 685 (19.89) | 679 (19.72) | 0.00 | 59 (2.31) | 62 (2.42) | 0.01 | 120 (7.81) | 114 (7.42) | 0.01 | 57 (0.68) | 49 (0.58) | 0.01 | 0 (0.00) | 0 (0.00) | 0.00 | <5 | <5 | 0.00 | 6 (4.72) | 8 (6.30) | 0.07 |
| Initiation of the study drug (empagliflozin/any SGLT-2 inhibitor/any DPP-4 inhibitor) as monotherapy | - | - | - | 44 (0.76) | 49 (0.84) | 0.01 | - | - | - | 24 (2.44) | 29 (2.95) | 0.03 | 894 (41.54) | 1013 (47.07) | 0.11 | 333 (9.67) | 316 (9.18) | 0.02 | 48 (1.88) | 48 (1.88) | 0.00 | 692 (45.05) | 693 (45.12) | 0.00 | 40 (0.47) | 34 (0.40) | 0.01 | 562 (14.45) | 559 (14.37) | 0.00 | 226 (98.69) | 227 (99.13) | 0.04 | <5 | 7 (5.51) | 0.12 |
| Dual therapy with metformin (without the use of other antidiabetic drugs) | - | - | - | <5 | <5 | 0.01 | - | - | - | 449 (45.68) | 452 (45.98) | 0.01 | 96 (4.46) | 80 (3.72) | 0.04 | 371 (10.77) | 356 (10.34) | 0.01 | 129 (5.04) | 122 (4.77) | 0.01 | - | - | - | 353 (4.19) | 342 (4.06) | 0.01 | 1541 (39.62) | 1565 (40.24) | 0.01 | <5 | <5 | 0.00 | 46 (36.22) | 44 (34.65) | 0.03 |
| Sulfonylureas 2nd generation (concomitant initiation or current use) | - | - | - | 12 (0.21) | 15 (0.26) | 0.01 | - | - | - | 115 (11.70) | 116 (11.80) | 0.00 | - | - | - | 1151 (33.42) | 1166 (33.86) | 0.01 | 451 (17.62) | 470 (18.37) | 0.02 | 36 (2.34) | 43 (2.80) | 0.03 | 879 (10.43) | 880 (10.44) | 0.00 | - | - | - | 5 (2.18) | 7 (3.06) | 0.06 | 45 (35.43) | 42 (33.07) | 0.05 |
| Insulin (concomitant initiation or current use) | - | - | - | 240 (4.12) | 242 (4.16) | 0.00 | - | - | - | 192 (19.53) | 189 (19.23) | 0.01 | - | - | - | 319 (9.26) | 337 (9.79) | 0.02 | 323 (12.62) | 343 (13.40) | 0.02 | 583 (37.96) | 578 (37.63) | 0.01 | 2192 (26.01) | 2284 (27.10) | 0.02 | - | - | - | <5 | <5 | 0.00 | 17 (13.39) | 21 (16.54) | 0.09 |

**CABG** - coronary artery bypass grafting; **CHF** - congestive heart failure; **DPP-4i -** dipeptidyl peptidase-4 inhibitor; **HbA1c** - Glycated hemoglobin; **Max** - maximum; **Min** - minimum; **PTCA** - percutaneous transluminal coronary angioplasty; **Q1** - 1st quartile; **Q3** - 3rd quartile; **SD** - standard deviation; **SGLT2i** - sodium-glucose cotransporter-2 inhibitor; **Std Diff** - standardized difference.

The combined comorbidity score is a single numeric comorbidity score for predicting short and long-term mortality, by combining conditions in the Charlson and Elixhauser comorbidity measures. Presence of diagnosis codes indicating existence of certain conditions may themselves be indicators for other factors that are inversely associated with 1-year mortality or may reflect idiosyncrasies of administrative data which is why negative values can occur. However, the underlying diagnoses should not be regarded as having a preventive/protective effect. JJ Gagne et al. *A combined comorbidity score predicted mortality in elderly patients better than existing scores.* J Clin Epidemiol. 2011 July ; 64(7): 749–759. doi:10.1016/j.jclinepi.2010.10.004.

Lookback period for combined comorbidity score is ever before index date for Denmark, Finland, Norway, Sweden and UK THIN and 12 months prior to the index date for other countries.

Lookback period for comorbidities and diabetes medication use is ever before index date for Denmark, Finland, Norway, Sweden and UK THIN, 6 months prior to the index date for Germany and 12 months or ever before for UK CPRD depending on covariate. For other countries, it is 12 months prior to the index date.

## Table S5c. Baseline characteristics in the empagliflozin and DPP-4i sub-cohorts after PS matching among patients without pre-existing congestive heart failure

| **Variable** | **Denmark** | | | **Finland** | | | **Germany** | | | **Israel** | | | **Japan** | | | **South Korea** | | | **Norway** | | | **Spain** | | | **Sweden** | | | **Taiwan** | | | **UK CPRD** | | | **UK THIN** | | |
| --- | --- | --- | --- | --- | --- | --- | --- | --- | --- | --- | --- | --- | --- | --- | --- | --- | --- | --- | --- | --- | --- | --- | --- | --- | --- | --- | --- | --- | --- | --- | --- | --- | --- | --- | --- | --- |
|  | **EMPA** | **DPP4i** | **Std Diff** | **EMPA** | **DPP4i** | **Std Diff** | **EMPA** | **DPP4i** | **Std Diff** | **EMPA** | **DPP4i** | **Std Diff** | **EMPA** | **DPP4i** | **Std Diff** | **EMPA** | **DPP4i** | **Std Diff** | **EMPA** | **DPP4i** | **Std Diff** | **EMPA** | **DPP4i** | **Std Diff** | **EMPA** | **DPP4i** | **Std Diff** | **EMPA** | **DPP4i** | **Std Diff** | **EMPA** | **DPP4i** | **Std Diff** | **EMPA** | **DPP4i** | **Std Diff** |
| **Number of patients** | 9252 | 9252 |  | 10999 | 10999 |  | 763 | 763 |  | 3782 | 3782 |  | 4065 | 4065 |  | 8510 | 8510 |  | 5977 | 5977 |  | 5698 | 5698 |  | 14557 | 14557 |  | 13186 | 13186 |  | 762 | 762 |  | 1270 | 1270 |  |
| **Age at index** | | | | | | | | | | | | | | | | | | | | | | | | | | | | | | | | | | | | |
| Mean | - | - | - | 62.05 | 61.9 | 0.01 | 62.21 | 62.52 | 0.03 | 61.07 | 61.28 | 0.02 | 56.97 | 57.07 | 0.01 | 55.51 | 55.51 | 0.00 | 60.37 | 60.55 | 0.01 | 64.94 | 64.94 | 0.00 | 62.95 | 62.84 | 0.01 | 56.59 | 56.42 | 0.01 | 57.19 | 57.77 | 0.05 | 59.2 | 59.45 | 0.02 |
| SD | - | - | - | 11.06 | 11.82 | - | 10.44 | 11.2 | - | 10.63 | 11.1 | - | 13.52 | 14.22 | - | 12.42 | 12.55 | - | 11.88 | 12.39 | - | 10.93 | 11.54 | - | 10.91 | 11.36 | - | 12.42 | 13.1 | - | 11.4 | 11.45 | - | 11.15 | 11.84 | - |
| Median | - | - | - | 63 | 63 | - | 62.96 | 63.42 | - | 61.81 | 62.02 | - | 58 | 58 | - | 56 | 56 | - | 61 | 61 | - | 65 | 65 | - | 64 | 64 | - | 57.32 | 56.91 | - | 57.39 | 57.42 | - | 59 | 59 | - |
| Q1 ; Q3 | - | - | - | 55.00 ; 70.00 | 55.00 ; 70.00 | - | 55.38 ; 69.80 | 55.32 ; 70.58 | - | 53.85 ; 68.68 | 53.79 ;   68.84 | - | 47.0 ; 67.0 | 47.0 ; 68.0 | - | 47 ; 64 | 47 ; 64 | - | 52.00 ; 69.00 | 52.00 ; 69.00 | - | 58.00 ; 73.00 | 57.00 ; 73.00 | - | 56.00 ; 71.00 | 56.00 ; 71.00 | - | 48.38 ; 65.13 | 47.66 ; 65.26 | - | 49.83 ; 65.24 | 50.38 ; 65.70 | - | 52.00 ; 67.00 | 51.00 ; 67.00 | - |
| Min ; Max | - | - | - | 18.00 ; 92.00 | 18.00 ; 98.00 | - | - | - | - | 20.48 ; 93.40 | 18.61 ;   96.52 | - | 18.0 ; 90.0 | 18.0 ; 90.0 | - | 18 ; 94 | 18 ; 99 | - | 19.00 ; 97.00 | 18.00 ; 101.00 | - | 19.00 ; 97.00 | 22.00 ; 104.00 | - | 18.00 ; 95.00 | 18.00 ; 97.00 | - | 18.31 ; 94.71 | 18.01 ; 104.45 | - | 19.02 ; 86.11 | 22.57 ; 97.03 | - | 23.70 ; 90.38 | 18.55 ; 93.39 | - |
| 18-54 | - | - | - | 2721 (24.74) | 2737 (24.88) | - | 183 (23.98) | 182 (23.85) | - | 1054 (27.87) | 1060 (28.03) | - | 1729 (42.53) | 1724 (42.41) | - | 3801 (44.67) | 3792 (44.56) | 0.00 | 1855 (31.04) | 1802 (30.15) | - | 985 (17.29) | 987 (17.32) | - | 3227 (22.17) | 3257 (22.37) | - | 5631 (42.70) | 5884 (44.62) | - | 322 (42.26) | 319 (41.86) | 0.03 | 417 (32.83) | 451 (35.51) | - |
| 55-64 | - | - | - | 3350 (30.46) | 3357 (30.52) | - | 256 (33.55) | 243 (31.85) | - | 1289 (34.08) | 1274 (33.69) | - | 1009 (24.82) | 925 (22.76) | - | 2749 (32.30) | 2730 (32.08) | - | 1814 (30.35) | 1851 (30.97) | - | 1690 (29.66) | 1692 (29.69) | - | 4240 (29.13) | 4230 (29.06) | - | 4204 (31.88) | 3910 (29.65) | - | 245 (32.15) | 238 (31.23) | - | 454 (35.75) | 406 (31.97) | - |
| 65-74 | - | - | - | 3595 (32.68) | 3630 (33.00) | - | 236 (30.93) | 244 (31.98) | - | 1100 (29.09) | 1090 (28.82) | - | 957 (23.54) | 987 (24.28) | - | 1414 (16.62) | 1451 (17.05) | - | 1639 (27.42) | 1661 (27.79) | - | 1906 (33.45) | 1889 (33.15) | - | 5173 (35.54) | 5211 (35.80) | - | 2505 (19.00) | 2328 (17.66) | - | 159 (20.87) | 168 (22.05) | - | 312 (24.57) | 287 (22.60) | - |
| 75+ | - | - | - | 1333 (12.12) | 1275 (11.59) | - | 88 (11.53) | 94 (12.32) | - | 339 (8.96) | 358 (9.47) | - | 370 (9.10) | 429 (10.55) | - | 546 (6.42) | 537 (6.31) | - | 669 (11.19) | 663 (11.09) | - | 1117 (19.60) | 1130 (19.83) | - | 1917 (13.17) | 1859 (12.77) | - | 846 (6.42) | 1064 (8.07) | - | 36 (4.72) | 37 (4.86) | - | 87 (6.85) | 126 (9.92) | - |
| **Sex** | | | | | | | | | | | | | | | | | | | | | | | | | | | | | | | | | | | | |
| Female | - | - | - | 4595 (41.78) | 4571 (41.56) | 0.00 | 306 (40.10) | 314 (41.15) | 0.02 | 1432 (37.86) | 1422 (37.60) | 0.01 | 1447 (35.60) | 1373 (33.78) | 0.04 | 3671 (43.14) | 3708 (43.57) | 0.01 | 2205 (36.89) | 2247 (37.59) | 0.01 | 2302 (40.40) | 2325 (40.80) | 0.01 | 5233 (35.95) | 5236 (35.97) | 0.00 | 5552 (42.11) | 5478 (41.54) | 0.01 | 304 (39.90) | 304 (39.90) | 0.00 | 517 (40.71) | 525 (41.34) | 0.01 |
| Male | - | - | - | 6404 (58.22) | 6428 (58.44) | - | 457 (59.90) | 449 (58.85) | - | 2350 (62.14) | 2360 (62.40) | - | 2618 (64.40) | 2692 (66.22) | - | 4839 (56.86) | 4802 (56.43) | - | 3772 (63.11) | 3730 (62.41) | - | 3396 (59.60) | 3373 (59.20) | - | 9324 (64.05) | 9321 (64.03) | - | 7634 (57.89) | 7708 (58.46) | - | 458 (60.10) | 458 (60.10) | - | 753 (59.29) | 745 (58.66) | - |
| **HbA1c (%)** | | | | | | | | | | | | | | | | | | | | | | | | | | | | | | | | | | | | |
| Mean | - | - | - | 7.42 | 7.24 | 0.11 | 7.73 | 7.78 | 0.04 | 8.1 | 8.12 | 0.01 | 8.23 | 8.16 | 0.04 | - | - | - | - | - | - | 7.23 | 7.18 | 0.04 | 8.13 | 8.12 | 0.00 | - | - | - | 9.25 | 9.22 | 0.02 | 9.11 | 9.12 | 0.01 |
| SD | - | - | - | 3.80 | 3.74 | - | 1.43 | 1.52 | - | 1.67 | 1.74 | - | 1.87 | 1.9 | - | - | - | - | - | - | - | 1.34 | 1.35 | - | 3.55 | 3.58 | - | - | - | - | 1.55 | 1.78 | - | 1.61 | 1.55 | - |
| Median | - | - | - | 7 | 6.80 | - | 7.48 | 7.53 | - | 7.7 | 7.7 | - | 7.68 | 7.57 | - | - | - | - | - | - | - | 6.9 | 6.8 | - | 7.82 | 7.82 | - | - | - | - | 9.01 | 8.92 | - | 8 | 8 | - |
| Q1 ; Q3 | - | - | - | 6.27 ; 8.10 | 6.18 ; 7.80 | - | 6.77 ; 8.35 | 6.75 ; 8.51 | - | 6.90 ; 8.90 | 6.90 ; 8.80 | - | 6.94 ; 9.13 | 6.81 ; 8.96 | - | - | - | - | - | - | - | 6.30 ; 7.80 | 6.30 ; 7.70 | - | 7.18 ; 8.83 | 7.18 ; 8.74 | - | - | - | - | 8.19 ; 10.20 | 8.01 ; 10.02 | - | 8.00 ; 10.00 | 8.00 ; 10.00 | - |
| Min ; Max | - | - | - | 4.44 ; 17.52 | 3.98 ; 17.25 | - | - | - | - | 4.70 ; 16.60 | 4.30 ;   16.30 | - | 5.4 ; 18.0 | 5.32 ; 17.5 | - | - | - | - | - | - | - | 5.00 ; 16.40 | 5.00 ; 15.40 | - | 4.71 ; 16.15 | 4.44 ; 16.70 | - | - | - | - | 3.20 ; 15.78 | 5.34 ; 17.34 | - | 3.07 ; 14.78 | 5.44 ; 14.87 | - |
| Missing | - | - | - | 6329 (57.54) | 6250 (56.82) | - | - | - | - | 90 | 73 | - | 3630 (89.30) | 3711 (91.29) | - | - | - | - | - | - | - | 3234 (56.60) | 2317 (59.30) | - | - | - | - | - | - | - | - | - | - | - | - | - |
| **Total cholesterol (mg/dl)** | | | | | | | | | | | | | | | | | | | | | | | | | | | | | | | | | | | | |
| Mean | - | - | - | 179.91 | 179.59 | 0.01 | 182.29 | 187.9 | - | 174.99 | 174.14 | 0.02 | 194.44 | 194.25 | 0.00 | - | - | - | - | - | - | 181.44 | 184.67 | 0.08 | 174.96 | 174.77 | 0.00 | - | - | - | 175.35 | 174.33 | 0.02 | 172.87 | 172.78 | 0.00 |
| SD | - | - | - | 45.4 | 44.44 | - | 44.08 | 46.81 | - | 46.81 | 47.39 | - | 41.08 | 53.03 | - | - | - | - | - | - | - | 41.05 | 42.87 | - | 44.93 | 44.45 | - | - | - | - | 41.79 | 43.75 | - | 45.2 | 45.21 | - |
| Median | - | - | - | 174.01 | 174.01 | - | 177.5 | 183 | - | 167 | 167 | - | 190 | 187.36 | - | - | - | - | - | - | - | 178 | 181 | - | 170.15 | 170.15 | - | - | - | - | 170.15 | 166.28 | - | 166 | 166 | - |
| Q1 ; Q3 | - | - | - | 146.95 ; 204.95 | 146.95 ; 204.95 | - | 150.00 ; 207.00 | 154.00 ; 218.00 | - | 142.00 ;   201.00 | 141.00 ;   200.00 | - | 166.5 ; 213.8 | 166.4 ; 213.5 | - | - | - | - | - | - | - | 153.00 ; 205.00 | 154.00 ; 211.00 | - | 143.08 ; 201.08 | 143.08 ; 201.08 | - | - | - | - | 143.08 ; 201.08 | 143.08 ; 197.22 | - | 139.00 ; 201.00 | 139.00 ; 197.00 | - |
| Min ; Max | - | - | - | 61.87 ; 564.58 | 58.01 ; 576.18 | - | - | - | - | 78.00 ; 546.00 | 55.00 ;   607.00 | - | 107.0 ; 487.0 | 96.0 ; 768.0 | - | - | - | - | - | - | - | 72.00 ; 578.00 | 76.00 ; 411.00 | - | 46.40 ; 549.11 | 54.14 ; 696.06 | - | - | - | - | 73.47 ; 371.23 | 88.94 ; 355.76 | - | 77.34 ; 398.30 | 85.07 ; 402.17 | - |
| Missing | - | - | - | 6180 (56.19) | 6158 (55.99) | - | 218 (28.57) | 275 (36.04) | - | 104 | 90 | - | 3706 (91.17) | 3811 (93.75) | - | - | - | - | - | - | - | 2854 (50.10) | 2665 (53.20) | - | - | - | - | - | - | - | - | - | - | - | - | - |
| **Low-density lipoprotein level (mg/dl)** | | | | | | | | | | | | | | | | | | | | | | | | | | | | | | | | | | | | |
| Mean | - | - | - | 107.37 | 107.15 | 0.01 | 108.9 | 113.31 | - | 93.63 | 92.38 | 0.04 | 116.87 | 115.14 | 0.04 | - | - | - | - | - | - | 102.22 | 103.81 | 0.05 | 96.42 | 96.14 | 0.00 | - | - | - | 115.71 | 114.48 | 0.03 | 96.29 | 96.25 | 0.00 |
| SD | - | - | - | 38.49 | 38.19 | - | 36.62 | 39.11 | - | 35.96 | 35.08 | - | 33.94 | 42.99 | - | - | - | - | - | - | - | 33.77 | 34.38 | - | 37.7 | 37.26 | - | - | - | - | 42.76 | 44.71 | - | 42.93 | 41.56 | - |
| Median | - | - | - | 100.54 | 100.54 | - | 105 | 108 | - | 88.2 | 86.6 | - | 111.63 | 110.9 | - | - | - | - | - | - | - | 98 | 101 | - | 90.49 | 90.49 | - | - | - | - | 111.08 | 108.28 | - | 88 | 88 | - |
| Q1 ; Q3 | - | - | - | 77.34 ; 131.48 | 77.34 ; 131.48 | - | 82.50 ; 133.50 | 85.00 ; 139.00 | - | 67.40 ; 114.00 | 66.70 ;   112.50 | - | 93.78 ; 132.9 | 91.06 ; 133.9 | - | - | - | - | - | - | - | 78.00 ; 123.00 | 79.00 ; 125.00 | - | 69.61 ; 118.72 | 69.61 ; 117.94 | - | - | - | - | 85.07 ; 141.14 | 81.21 ; 135.34 | - | 65.00 ; 119.00 | 65.00 ; 119.00 | - |
| Min ; Max | - | - | - | 7.73 ; 309.36 | 7.73 ; 282.29 | - | - | - | - | 3.70 ; 273.40 | 10.10 ;   281.60 | - | 50.98 ; 291.0 | 37.0 ; 571.0 | - | - | - | - | - | - | - | 16.40 ; 261.00 | 0.00 ; 265.00 | - | 8.89 ; 320.96 | 8.89 ; 324.83 | - | - | - | - | 15.47 ; 328.69 | 15.47 ; 313.23 | - | 11.60 ; 337.83 | 11.60 ; 282.29 | - |
| Missing | - | - | - | 6322 (57.48) | 6271 (57.01) | - | 266 (34.86) | 339 (44.43) | - | 457 | 447 | - | 3739 (91.98) | 3802 (93.53) | - | - | - | - | - | - | - | 2724 (48.30) | 2671 (53.10) | - | - | - | - | - | - | - | - | - | - | - | - | - |
| **High-density lipoprotein level (mg/dl)** | | | | | | | | | | | | | | | | | | | | | | | | | | | | | | | | | | | | |
| Mean | - | - | - | 47.28 | 47.91 | 0.05 | 48.36 | 45.06 | - | 43.05 | 42.84 | 0.02 | 49.23 | 50.12 | 0.06 | - | - | - | - | - | - | 46.3 | 46.64 | 0.03 | 45.02 | 45.01 | 0.00 | - | - | - | 44.21 | 44.35 | 0.01 | 43.64 | 43.29 | 0.03 |
| SD | - | - | - | 13.42 | 14.15 | - | 24.93 | 12.84 | - | 10.39 | 10.54 | - | 13.13 | 15.49 | - | - | - | - | - | - | - | 12.31 | 12.62 | - | 13 | 13.09 | - | - | - | - | 11.25 | 12.05 | - | 11.9 | 11.04 | - |
| Median | - | - | - | 45.24 | 45.63 | - | 44 | 43 | - | 42 | 41 | - | 47.33 | 47.75 | - | - | - | - | - | - | - | 45 | 45 | - | 42.54 | 42.54 | - | - | - | - | 42.54 | 42.54 | - | 42 | 42 | - |
| Q1 ; Q3 | - | - | - | 38.28 ; 54.14 | 38.28 ; 54.91 | - | 37.00 ; 54.00 | 37.00 ; 51.50 | - | 36.00 ; 49.00 | 36.00 ;   48.00 | - | 40.18 ; 55.95 | 40.5 ; 56.67 | - | - | - | - | - | - | - | 38.00 ; 53.00 | 38.00 ; 53.00 | - | 34.80 ; 50.27 | 34.80 ; 50.27 | - | - | - | - | 37.12 ; 50.27 | 35.96 ; 50.27 | - | 34.00 ; 50.00 | 34.00 ; 50.00 | - |
| Min ; Max | - | - | - | 3.87 ; 141.15 | 8.89 ; 204.95 | - | - | - | - | 8.00 ; 99.00 | 13.00 ;   110.00 | - | 19.67 ; 104.0 | 19.75 ; 160.0 | - | - | - | - | - | - | - | 20.00 ; 124.80 | 20.00 ; 112.00 | - | 7.73 ; 170.15 | 7.73 ; 174.01 | - | - | - | - | 15.47 ; 116.40 | 15.47 ; 123.74 | - | 11.60 ; 146.95 | 15.47 ; 112.14 | - |
| Missing | - | - | - | 6196 (56.33) | 6193 (56.31) | - | 268 (35.12) | 325 (42.60) | - | 104 | 94 | - | 3680 (90.53) | 3772 (92.79) | - | - | - | - | - | - | - | 2663 (46.70) | 2756 (51.60) | - | - | - | - | - | - | - | - | - | - | - | - | - |
| **Triglyceride level (mg/dl)** | | | | | | | | | | | | | | | | | | | | | | | | | | | | | | | | | | | | |
| Mean | - | - | - | 188.53 | 183.95 | 0.03 | 204.54 | 212.71 | - | 205.21 | 203.22 | 0.01 | 197.45 | 185.05 | 0.08 | - | - | - | - | - | - | 154.65 | 155.99 | 0.02 | 192.51 | 192.03 | 0.00 | - | - | - | 206.63 | 202.8 | 0.02 | 212.98 | 217.74 | 0.04 |
| SD | - | - | - | 141.37 | 134.79 | - | 132.12 | 137.99 | - | 182.63 | 186.92 | - | 153.67 | 154.57 | - | - | - | - | - | - | - | 71.27 | 72.17 | - | 152.31 | 142.51 | - | - | - | - | 187.02 | 147.8 | - | 128.44 | 137.08 | - |
| Median | - | - | - | 156.77 | 155 | - | 169 | 169.25 | - | 165 | 165 | - | 163.3 | 143 | - | - | - | - | - | - | - | 139 | 141 | - | 159.43 | 159.43 | - | - | - | - | 175.27 | 168.29 | - | 177 | 185 | - |
| Q1 ; Q3 | - | - | - | 115.14 ; 218.77 | 111.60 ; 216.11 | - | 121.00 ; 237.00 | 129.75 ; 249.50 | - | 119.00 ;   234.00 | 116.00 ;   232.00 | - | 108.67 ; 230.1 | 103.0 ; 205.5 | - | - | - | - | - | - | - | 103.00 ; 189.00 | 101.00 ; 194.00 | - | 115.14 ; 221.43 | 115.14 ; 230.28 | - | - | - | - | 124.00 ; 239.15 | 119.57 ; 239.15 | - | 132.00 ; 256.00 | 124.00 ; 265.00 | - |
| Min ; Max | - | - | - | 29.23 ; 2941.41 | 26.57 ; 3099.06 | - | - | - | - | 39.00 ;   3490.00 | 35.00 ;   4120.00 | - | 42.0 ; 1332.0 | 40.0 ; 1783.0 | - | - | - | - | - | - | - | 41.00 ; 400.00 | 40.00 ; 399.00 | - | 8.86 ; 3099.95 | 26.57 ; 3011.38 | - | - | - | - | 0.00 ; 3348.10 | 0.00 ; 1389.73 | - | 44.29 ; 956.60 | 44.29 ; 974.31 | - |
| Missing | - | - | - | 6189 (56.27) | 6186 (56.24) | - | 233 (30.54) | 307 (40.24) | - | 104 | 90 | - | 3668 (90.23) | 3754 (92.35) | - | - | - | - | - | - | - | 2642 (46.40) | 2798 (50.90) | - | - | - | - | - | - | - | - | - | - | - | - | - |
| **Estimated Glomerular Filtration Rate (mL/min/1,73m^2^)** | | | | | | | | | | | | | | | | | | | | | | | | | | | | | | | | | | | | |
| Mean | - | - | - | 88.79 | 85.92 | 0.15 | 82.1 | 77.45 | - | 89.66 | 89.74 | 0.00 | - | - | - | - | - | - | - | - | - | 85.48 | 81.64 | 0.20 | 88.21 | 88.13 | 0.02 | - | - | - | 88.48 | 88.18 | 0.02 | 89.27 | 88.95 | 0.02 |
| SD | - | - | - | 16.63 | 21.24 | - | 19.75 | 23.17 | - | 17.61 | 19.72 | - | - | - | - | - | - | - | - | - | - | 17.19 | 20.48 | - | 17.14 | 18.88 | - | - | - | - | 17.89 | 19.14 | - | 17.07 | 16.86 | - |
| Median | - | - | - | 90.35 | 89.83 | - | 85.07 | 81.16 | - | 92.41 | 93.6 | - | - | - | - | - | - | - | - | - | - | 88.07 | 85.84 | - | 90.19 | 91.11 | - | - | - | - | 89.63 | 89 | - | 90 | 90 | - |
| Q1 ; Q3 | - | - | - | 77.88 ; 99.86 | 72.89 ; 101.01 | - | 68.50 ; 96.39 | 62.43 ; 94.71 | - | 78.45 ; 101.91 | 79.40 ;   102.96 | - | - | - | - | - | - | - | - | - | - | 75.37 ; 97.19 | 68.18 ; 96.49 | - | 77.41 ; 99.42 | 76.56 ; 101.04 | - | - | - | - | 76.00 ; 102.73 | 76.00 ; 102.12 | - | 77.00 ; 101.00 | 77.00 ; 100.00 | - |
| Min ; Max | - | - | - | 19.34 ; 157.19 | 7.36 ; 175.46 | - | - | - | - | 27.17 ; 155.93 | 17.59 ;   146.93 | - | - | - | - | - | - | - | - | - | - | 19.93 ; 271.83 | 5.30 ; 133.17 | - | 14.76 ; 216.17 | 23.65 ; 170.51 | - | - | - | - | 2.00 ; 143.04 | 21.00 ; 138.00 | - | 30.23 ; 139.28 | 33.49 ; 153.16 | - |
| Missing | - | - | - | 5586 (50.79) | 5503 (50.03) | - | 125 (16.38) | 182 (23.85) | - | 84 | 71 | - | - | - | - | - | - | - | - | - | - | 3051 (53.50) | 2569 (54.90) | - | - | - | - | - | - | - | - | - | - | - | - | - |
| **Creatinine (mg/dl)** | | | | | | | | | | | | | | | | | | | | | | | | | | | | | | | | | | | | |
| Mean | - | - | - | 0.82 | 0.88 | 0.22 | 0.92 | 1.02 | - | 0.83 | 0.83 | 0.02 | 0.8 | 0.78 | 0.06 | - | - | - | - | - | - | 0.84 | 0.91 | 0.22 | 0.83 | 0.83 | 0.01 | - | - | - | 0.83 | 0.84 | 0.01 | - | - | - |
| SD | - | - | - | 0.19 | 0.29 | - | 0.39 | 0.56 | - | 0.22 | 0.26 | - | 0.36 | 0.27 | - | - | - | - | - | - | - | 0.22 | 0.35 | - | 0.2 | 0.22 | - | - | - | - | 0.18 | 0.21 | - | - | - | - |
| Median | - | - | - | 0.8 | 0.82 | - | 0.87 | 0.9 | - | 0.8 | 0.78 | - | 0.73 | 0.73 | - | - | - | - | - | - | - | 0.81 | 0.85 | - | 0.81 | 0.8 | - | - | - | - | 0.81 | 0.81 | - | - | - | - |
| Q1 ; Q3 | - | - | - | 0.69 ; 0.93 | 0.70 ; 0.98 | - | 0.73 ; 1.03 | 0.77 ; 1.12 | - | 0.67 ; 0.95 | 0.66 ; 0.93 | - | 0.6 ; 0.9 | 0.62 ; 0.88 | - | - | - | - | - | - | - | 0.70 ; 0.96 | 0.71 ; 1.01 | - | 0.69 ; 0.95 | 0.68 ; 0.95 | - | - | - | - | 0.70 ; 0.94 | 0.70 ; 0.93 | - | - | - | - |
| Min ; Max | - | - | - | 0.35 ; 3.27 | 0.24 ; 7.74 | - | - | - | - | 0.30 ; 2.22 | 0.30 ; 3.26 | - | 0.36 ; 4.86 | 0.18 ; 2.89 | - | - | - | - | - | - | - | 0.06 ; 2.18 | 0.33 ; 10.70 | - | 0.12 ; 4.43 | 0.14 ; 2.36 | - | - | - | - | 0.42 ; 1.76 | 0.37 ; 1.96 | - | - | - | - |
| Missing | - | - | - | 5580 (50.73) | 5502 (50.02) | - | 125 (16.38) | 182 (23.85) | - | 84 | 71 | - | 3628 (89.25) | 3692 (90.82) | - | - | - | - | - | - | - | 3051 (53.50) | 2569 (54.90) | - | - | - | - | - | - | - | - | - | - | - | - | - |
| **Combined comorbidity score** | | | | | | | | | | | | | | | | | | | | | | | | | | | | | | | | | | | | |
| Mean | - | - | - | - | - | - | - | - | - | - | - | - | 0.52 | 0.5 | 0.02 | 0.3 | 0.27 | 0.04 | - | - | - | 0.27 | 0.25 | 0.01 | - | - | - | 0.55 | 0.56 | 0.01 | 0.13 | 0.18 | 0.07 | - | - | - |
| SD | - | - | - | - | - | - | - | - | - | - | - | - | 1.32 | 1.27 | - | 0.97 | 0.96 | - | - | - | - | 1.21 | 1.15 | - | - | - | - | 1.22 | 1.26 | - | 0.54 | 0.71 | - | - | - | - |
| Median | - | - | - | - | - | - | - | - | - | - | - | - | 0 | 0 | - | 0 | 0 | - | - | - | - | 0 | 0 | - | - | - | - | 0 | 0 | - | 0 | 0 | - | - | - | - |
| Q1 ; Q3 | - | - | - | - | - | - | - | - | - | - | - | - | 0.0 ; 1.0 | 0.0 ; 1.0 | - | 0 ; 1 | 0 ; 1 | - | - | - | - | 0.00 ; 0.00 | 0.00 ; 0.00 | - | - | - | - | 0.00 ; 1.00 | 0.00 ; 1.00 | - | 0.00 ; 0.00 | 0.00 ; 0.00 | - | - | - | - |
| Min ; Max | - | - | - | - | - | - | - | - | - | - | - | - | -1.0 ; 10.0 | -1.0 ; 9.0 | - | -1 ; 9 | -1 ; 7 | - | - | - | - | -1.00 ; 12.00 | -1.00 ; 12.00 | - | - | - | - | -1.00 ; 9.00 | -1.00 ; 11.00 | - | 0.00 ; 6.00 | 0.00 ; 8.00 | - | - | - | - |
| Missing | - | - | - | - | - | - | - | - | - | - | - | - | - | - | - | - | - | - | - | - | - | - | - | - | - | - | - | - | - | - | - | - | - | - | - | - |
| **Comorbidities and other medical conditions** | | | | | | | | | | | | | | | | | | | | | | | | | | | | | | | | | | | | |
| Diabetic retinopathy | - | - | - | 349 (3.17) | 359 (3.26) | 0.01 | 26 (3.41) | 35 (4.59) | 0.07 | 506 (13.38) | 504 (13.33) | 0.00 | 661 (16.26) | 637 (15.67) | 0.02 | 1675 (19.68) | 1622 (19.06) | 0.02 | 506 (8.47) | 525 (8.78) | 0.01 | 53 (0.93) | 53 (0.93) | 0.00 | 1472 (10.11) | 1456 (10.00) | 0.00 | 1290 (9.78) | 1310 (9.93) | 0.01 | 0 (0.00) | 0 (0.00) | - | 196 (15.43) | 187 (14.72) | 0.02 |
| Diabetic neuropathy | - | - | - | 103 (0.94) | 113 (1.03) | 0.01 | 580 (76.02) | 528 (69.20) | - | 532 (14.07) | 565 (14.94) | 0.02 | 143 (3.52) | 153 (3.76) | 0.01 | 1421 (16.70) | 1423 (16.72) | 0.00 | 94 (1.57) | 99 (1.66) | 0.01 | 83 (1.46) | 87 (1.53) | 0.01 | 192 (1.32) | 194 (1.33) | 0.00 | 901 (6.83) | 938 (7.11) | 0.01 | <5 | <5 | 0.04 | 66 (5.20) | 63 (4.96) | 0.01 |
| Diabetic nephropathy | - | - | - | 93 (0.85) | 94 (0.85) | 0.00 | 84 (11.01) | 92 (12.06) | 0.04 | 243 (6.43) | 251 (6.64) | 0.01 | 327 (8.04) | 327 (8.04) | 0.00 | 1195 (14.04) | 1169 (13.74) | 0.01 | 55 (0.92) | 52 (0.87) | 0.01 | 44 (0.77) | 42 (0.74) | 0.00 | 123 (0.84) | 133 (0.91) | 0.01 | 2570 (19.49) | 2584 (19.60) | 0.00 | 0 (0.00) | 0 (0.00) | - | <5 | 0 (0.00) | 0.04 |
| Diabetes with peripheral circulatory disorders | - | - | - | 99 (0.90) | 104 (0.95) | 0.00 | - | - | - | 80 (2.12) | 80 (2.12) | 0.00 | 32 (0.79) | 28 (0.69) | 0.01 | 33 (0.39) | 30 (0.35) | 0.01 | 47 (0.79) | 45 (0.75) | 0.00 | 113 (1.98) | 120 (2.11) | 0.01 | 107 (0.74) | 119 (0.82) | 0.01 | 164 (1.24) | 155 (1.18) | 0.01 | 0 (0.00) | 0 (0.00) | - | 0 (0.00) | 0 (0.00) | - |
| Diabetic foot | - | - | - | 0 (0.00) | 0 (0.00) | - | 176 (23.07) | 178 (23.33) | - | 18 (0.48) | 21 (0.56) | 0.01 | 18 (0.44) | 18 (0.44) | 0.00 | - | - | - | - | - | - | 33 (0.58) | 37 (0.65) | 0.01 | 91 (0.63) | 96 (0.66) | 0.00 | 23 (0.17) | 22 (0.17) | 0.00 | 0 (0.00) | 0 (0.00) | - | 998 (78.58) | 977 (76.93) | 0.04 |
| Hypoglycemia | - | - | - | 35 (0.32) | 37 (0.34) | 0.00 | <5 | <5 | - | 71 (1.88) | 62 (1.64) | 0.02 | 23 (0.57) | 18 (0.44) | 0.02 | 84 (0.99) | 80 (0.94) | 0.01 | 12 (0.20) | 10 (0.17) | 0.01 | 68 (1.19) | 56 (0.98) | 0.02 | 107 (0.74) | 110 (0.76) | 0.00 | 66 (0.50) | 58 (0.44) | 0.01 | 0 (0.00) | 0 (0.00) | - | 6 (0.47) | <5 | 0.04 |
| Hyperlipidemia | - | - | - | 1696 (15.42) | 1682 (15.29) | 0.00 | 551 (72.21) | 528 (69.20) | - | 1062 (28.08) | 1066 (28.19) | 0.00 | 2244 (55.20) | 2229 (54.83) | 0.01 | 7344 (86.30) | 7327 (86.10) | 0.01 | 719 (12.03) | 706 (11.81) | 0.01 | 1005 (17.64) | 1024 (17.97) | 0.01 | 2753 (18.91) | 2726 (18.73) | 0.00 | 9063 (68.73) | 9038 (68.54) | 0.00 | 208 (27.30) | 214 (28.08) | 0.02 | 0 (0.00) | 0 (0.00) | - |
| Ischemic heart disease | - | - | - | 1415 (12.86) | 1361 (12.37) | 0.01 | - | - | - | 502 (13.27) | 507 (13.41) | 0.00 | 786 (19.34) | 801 (19.70) | 0.01 | 1573 (18.48) | 1609 (18.91) | 0.01 | 1147 (19.19) | 1133 (18.96) | 0.01 | 414 (7.27) | 397 (6.97) | 0.01 | 2831 (19.45) | 2805 (19.27) | 0.00 | 2351 (17.83) | 2339 (17.74) | 0.00 | 92 (12.07) | 92 (12.07) | 0.00 | 28 (2.20) | 31 (2.44) | 0.02 |
| Stable angina | - | - | - | 557 (5.06) | 536 (4.87) | 0.01 | - | - | - | 47 (1.24) | 52 (1.37) | 0.01 | 530 (13.04) | 524 (12.89) | 0.00 | 336 (3.95) | 357 (4.20) | 0.01 | 481 (8.05) | 440 (7.36) | 0.03 | 324 (5.69) | 321 (5.63) | 0.00 | 1375 (9.45) | 1393 (9.57) | 0.00 | 630 (4.78) | 625 (4.74) | 0.00 | 51 (6.69) | 53 (6.96) | 0.01 | 12 (0.94) | 11 (0.87) | 0.01 |
| History of CABG or PTCA | - | - | - | 213 (1.94) | 205 (1.86) | 0.01 | 56 (7.34) | 36 (4.72) | - | 114 (3.01) | 118 (3.12) | 0.01 | <5 | <5 | 0.01 | 244 (2.87) | 233 (2.74) | 0.01 | 95 (1.59) | 100 (1.67) | 0.01 | 11 (0.19) | 7 (0.12) | 0.02 | 1114 (7.65) | 1106 (7.60) | 0.00 | 19 (0.14) | 28 (0.21) | 0.02 | - | - | - | 18 (1.42) | 18 (1.42) | 0.00 |
| Any stroke | - | - | - | 479 (4.35) | 495 (4.50) | 0.01 | 33 (4.33) | 58 (7.60) | - | 159 (4.20) | 168 (4.44) | 0.01 | 204 (5.02) | 187 (4.60) | 0.02 | 470 (5.52) | 445 (5.23) | 0.01 | 199 (3.33) | 203 (3.40) | 0.00 | 99 (1.74) | 83 (1.46) | 0.02 | 744 (5.11) | 721 (4.95) | 0.01 | 581 (4.41) | 572 (4.34) | 0.00 | 21 (2.76) | 29 (3.81) | 0.06 | <5 | <5 | 0.02 |
| CHF | - | - | - | 0 (0.00) | 0 (0.00) | - | - | - | - | 0 (0.00) | 0 (0.00) | - | 0 (0.00) | 0 (0.00) | 0.00 | 145 (1.70) | 144 (1.69) | 0.00 | 0 (0.00) | 0 (0.00) | - | 0 (0.00) | 0 (0.00) | - | 0 (0.00) | 0 (0.00) | - | - | - | - | 0 (0.00) | 0 (0.00) | - | 0 (0.00) | 0 (0.00) | - |
| Peripheral Vascular disease or surgery | - | - | - | 339 (3.08) | 334 (3.04) | 0.00 | - | - | - | 107 (2.83) | 112 (2.96) | 0.01 | 104 (2.56) | 93 (2.29) | 0.02 | 1744 (20.49) | 1727 (20.29) | 0.00 | 1773 (29.66) | 1744 (29.18) | 0.01 | 71 (1.25) | 81 (1.42) | 0.02 | 652 (4.48) | 627 (4.31) | 0.01 | 212 (1.61) | 209 (1.59) | 0.00 | 24 (3.15) | 23 (3.02) | 0.01 | <5 | <5 | 0.00 |
| Hypertension | - | - | - | 2539 (23.08) | 2525 (22.96) | 0.00 | 646 (84.67) | 635 (83.22) | - | 1325 (35.03) | 1321 (34.93) | 0.00 | 2286 (56.24) | 2206 (54.27) | 0.04 | 5013 (58.91) | 5032 (59.13) | 0.00 | 302 (5.05) | 303 (5.07) | 0.00 | 889 (15.60) | 885 (15.53) | 0.00 | 3689 (25.34) | 3643 (25.03) | 0.01 | 8194 (62.14) | 8156 (61.85) | 0.01 | 471 (61.81) | 477 (62.60) | 0.02 | 85 (6.69) | 84 (6.61) | 0.00 |
| Chronic kidney disease | - | - | - | 28 (0.25) | 20 (0.18) | 0.02 | - | - | - | 102 (2.70) | 108 (2.86) | 0.01 | 95 (2.34) | 90 (2.21) | 0.01 | 121 (1.42) | 114 (1.34) | 0.01 | 46 (0.77) | 53 (0.89) | 0.01 | 28 (0.49) | 33 (0.58) | 0.01 | 54 (0.37) | 52 (0.36) | 0.00 | 714 (5.41) | 735 (5.57) | 0.01 | 0 (0.00) | 0 (0.00) | - | <5 | <5 | 0.02 |
| **Number of antidiabetic substances at index date** | | | | | | | | | | | | | | | | | | | | | | | | | | | | | | | | | | | | |
| Mean | - | - | - | 2.1 | 2.11 | 0.01 | - | - | - | 2.18 | 2.19 | 0.01 | 2.17 | 2.1 | 0.06 | 2.11 | 2.1 | 0.01 | 2.16 | 2.16 | 0.00 | 1.7 | 1.68 | 0.02 | 2.15 | 2.16 | 0.02 | 2.64 | 2.62 | - | 1.21 | 1.2 | 0.01 | 2.35 | 2.35 | 0.00 |
| SD | - | - | - | 0.77 | 0.78 | - | - | - | - | 0.7 | 0.69 | - | 1.15 | 1.13 | - | 0.7 | 0.69 | - | 0.84 | 0.82 | - | 0.98 | 0.97 | - | 0.72 | 0.73 | - | 1.1 | 1.03 | - | 0.5 | 0.47 | - | 0.72 | 0.73 | - |
| Median | - | - | - | 2 | 2 | - | - | - | - | 2 | 2 | - | 2 | 2 | - | 2 | 2 | - | 2 | 2 | - | 2 | 2 | - | 2 | 2 | - | 2 | 3 | - | 1 | 1 | - | 2 | 2 | - |
| Q1 ; Q3 | - | - | - | 2.00 ; 2.00 | 2.00 ; 2.00 | - | - | - | - | 2.00 ; 3.00 | 2.00 ; 3.00 | - | 1.0 ; 3.0 | 1.0 ; 3.0 | - | 2 ; 3 | 2 ; 3 | - | 2.00 ; 3.00 | 2.00 ; 3.00 | - | 1.00 ; 2.00 | 1.00 ; 2.00 | - | 2.00 ; 3.00 | 2.00 ; 3.00 | - | 2.00 ; 3.00 | 2.00 ; 3.00 | - | 1.00 ; 1.00 | 1.00 ; 1.00 | - | 2.00 ; 3.00 | 2.00 ; 3.00 | - |
| Min ; Max | - | - | - | 1.00 ; 7.00 | 1.00 ; 6.00 | - | - | - | - | 1.00 ; 5.00 | 1.00 ; 5.00 | - | 1.0 ; 7.0 | 1.0 ; 8.0 | - | 1 ; 5 | 1 ; 5 | - | 1.00 ; 7.00 | 1.00 ; 6.00 | - | 0.00 ; 6.00 | 0.00 ; 6.00 | - | 1.00 ; 6.00 | 1.00 ; 7.00 | - | 0.00 ; 10.00 | 0.00 ; 9.00 | - | 1.00 ; 6.00 | 1.00 ; 4.00 | - | 1.00 ; 4.00 | 1.00 ; 5.00 | - |
| **Diabetes medication use** | | | | | | | | | | | | | | | | | | | | | | | | | | | | | | | | | | | | |
| Naive new use of antidiabetic drugs | - | - | - | 170 (1.55) | 174 (1.58) | 0.00 | - | - | - | 444 (11.74) | 442 (11.69) | 0.00 | 1452 (35.72) | 1534 (37.74) | 0.04 | 2148 (25.24) | 2111 (24.81) | 0.01 | 169 (2.83) | 146 (2.44) | 0.02 | 365 (6.41) | 378 (6.63) | 0.01 | 66 (0.45) | 63 (0.43) | 0.00 | 0 (0.00) | 0 (0.00) | 0.00 | 10 (1.31) | 10 (1.31) | 0.00 | 28 (2.20) | 32 (2.52) | 0.02 |
| Initiation of the study drug (empagliflozin/any SGLT-2 inhibitor/any DPP-4 inhibitor) as monotherapy | - | - | - | 88 (0.80) | 86 (0.78) | 0.00 | - | - | - | 148 (3.91) | 145 (3.83) | 0.00 | 1027 (25.26) | 1094 (26.91) | 0.04 | 685 (8.05) | 686 (8.06) | 0.00 | 128 (2.14) | 122 (2.04) | 0.01 | 2706 (47.49) | 2782 (48.82) | 0.03 | 42 (0.29) | 40 (0.27) | 0.00 | 1468 (11.13) | 1558 (11.82) | 0.02 | 757 (99.34) | 758 (99.48) | 0.02 | 20 (1.57) | 21 (1.65) | 0.01 |
| Dual therapy with metformin (without the use of other antidiabetic drugs) | - | - | - | 6 (0.05) | 5 (0.05) | 0.00 | - | - | - | 1795 (47.46) | 1794 (47.44) | 0.00 | 442 (10.87) | 479 (11.78) | 0.03 | 839 (9.86) | 850 (9.99) | 0.00 | 317 (5.30) | 325 (5.44) | 0.01 | - | - | - | 676 (4.64) | 693 (4.76) | 0.01 | 4436 (33.64) | 4379 (33.21) | 0.01 | 14 (1.84) | 13 (1.71) | 0.01 | 519 (40.87) | 492 (38.74) | 0.04 |
| Sulfonylureas 2nd generation (concomitant initiation or current use) | - | - | - | 22 (0.20) | 29 (0.26) | 0.01 | - | - | - | 403 (10.66) | 389 (10.29) | 0.01 | - | - | - | 2571 (30.21) | 2581 (30.33) | 0.00 | 957 (16.01) | 1000 (16.73) | 0.02 | 134 (2.35) | 138 (2.42) | 0.00 | 1658 (11.39) | 1647 (11.31) | 0.00 | - | - | - | 17 (2.23) | 19 (2.49) | 0.02 | 420 (33.07) | 414 (32.60) | 0.01 |
| Insulin (concomitant initiation or current use) | - | - | - | 381 (3.46) | 398 (3.62) | 0.01 | - | - | - | 600 (15.86) | 619 (16.37) | 0.01 | - | - | - | 705 (8.28) | 698 (8.20) | 0.00 | 598 (10.01) | 601 (10.06) | 0.00 | 1842 (32.33) | 1761 (30.91) | 0.03 | 3419 (23.49) | 3480 (23.91) | 0.01 | - | - | - | 13 (1.71) | 13 (1.71) | 0.00 | 130 (10.24) | 142 (11.18) | 0.03 |

**CABG** - coronary artery bypass grafting; **CHF** - congestive heart failure; **DPP-4i -** dipeptidyl peptidase-4 inhibitor; **HbA1c** - Glycated hemoglobin; **Max** - maximum; **Min** - minimum; **PTCA** - percutaneous transluminal coronary angioplasty; **Q1** - 1st quartile; **Q3** - 3rd quartile; **SD** - standard deviation; **SGLT2i** - sodium-glucose cotransporter-2 inhibitor; **Std Diff** - standardized difference.

The combined comorbidity score is a single numeric comorbidity score for predicting short and long-term mortality, by combining conditions in the Charlson and Elixhauser comorbidity measures. Presence of diagnosis codes indicating existence of certain conditions may themselves be indicators for other factors that are inversely associated with 1-year mortality or may reflect idiosyncrasies of administrative data which is why negative values can occur. However, the underlying diagnoses should not be regarded as having a preventive/protective effect. JJ Gagne et al. *A combined comorbidity score predicted mortality in elderly patients better than existing scores.* J Clin Epidemiol. 2011 July ; 64(7): 749–759. doi:10.1016/j.jclinepi.2010.10.004.

Lookback period for combined comorbidity score is ever before index date for Denmark, Finland, Norway, Sweden and UK THIN and 12 months prior to the index date for other countries.

Lookback period for comorbidities and diabetes medication use is ever before index date for Denmark, Finland, Norway, Sweden and UK THIN, 6 months prior to the index date for Germany and 12 months or ever before for UK CPRD depending on covariate. For other countries, it is 12 months prior to the index date.

## Table S5d. Baseline characteristics in the empagliflozin and DPP-4i sub-cohorts after PS matching among patients with pre-existing congestive heart failure

| **Variable** | **Denmark** | | | **Finland** | | | **Germany** | | | **Israel** | | | **Japan** | | | **South Korea** | | | **Norway** | | | **Spain** | | | **Sweden** | | | **Taiwan** | | | **UK CPRD** | | | **UK THIN** | | |
| --- | --- | --- | --- | --- | --- | --- | --- | --- | --- | --- | --- | --- | --- | --- | --- | --- | --- | --- | --- | --- | --- | --- | --- | --- | --- | --- | --- | --- | --- | --- | --- | --- | --- | --- | --- | --- |
|  | **EMPA** | **DPP4i** | **Std Diff** | **EMPA** | **DPP4i** | **Std Diff** | **EMPA** | **DPP4i** | **Std Diff** | **EMPA** | **DPP4i** | **Std Diff** | **EMPA** | **DPP4i** | **Std Diff** | **EMPA** | **DPP4i** | **Std Diff** | **EMPA** | **DPP4i** | **Std Diff** | **EMPA** | **DPP4i** | **Std Diff** | **EMPA** | **DPP4i** | **Std Diff** | **EMPA** | **DPP4i** | **Std Diff** | **EMPA** | **DPP4i** | **Std Diff** | **EMPA** | **DPP4i** | **Std Diff** |
| **Number of patients** | 458 | 458 |  | 729 | 729 |  | 79 | 79 |  | 71 | 71 |  | 1525 | 1525 |  | 561 | 561 |  | 326 | 326 |  | 130 | 130 |  | 1127 | 1127 |  | 861 | 861 |  | - | - | - | - | - | - |
| **Age at index** | | | | | | | | | | | | | | | | | | | | | | | | | | | | | | | | | | | | |
| Mean | - | - | - | 69.27 | 69.32 | 0.00 | 68.22 | 68.26 | 0.00 | 69.81 | 73.13 | 0.34 | 65.46 | 66.2 | 0.06 | 62.65 | 62.32 | 0.03 | 67.2 | 67.36 | 0.01 | 73.22 | 73.57 | 0.03 | 69.66 | 69.35 | 0.03 | 62.15 | 62.46 | 0.02 | - | - | - | - | - | - |
| SD | - | - | - | 10.16 | 11.02 | - | 8.87 | 9.75 | - | 9.61 | 10.04 | - | 12.59 | 12.59 | - | 12.17 | 12.9 | - | 10.78 | 10.9 | - | 9.84 | 10.62 | - | 9.41 | 9.58 | - | 13.53 | 14.3 | - | - | - | - | - | - | - |
| Median | - | - | - | 70 | 70 | - | 70.79 | 69.04 | - | 68.59 | 73.04 | - | 67 | 68 | - | 63 | 64 | - | 68 | 68 | - | 74 | 75 | - | 70 | 70 | - | 63.21 | 62.23 | - | - | - | - | - | - | - |
| Q1 ; Q3 | - | - | - | 63.00 ; 76.00 | 63.00 ; 77.00 | - | 61.00 ; 74.85 | 61.73 ; 74.13 | - | 63.26 ; 76.90 | 66.62 ;   80.76 | - | 56.0 ; 75.0 | 58.0 ; 75.0 | - | 55 ; 72 | 53 ; 72 | - | 60.00 ; 74.00 | 61.00 ; 74.00 | - | 67.00 ; 81.00 | 66.00 ; 83.00 | - | 64.00 ; 76.00 | 64.00 ; 76.00 | - | 53.29 ; 71.78 | 52.34 ; 73.33 | - | - | - | - | - | - | - |
| Min ; Max | - | - | - | 35.00 ; 99.00 | 18.00 ; 94.00 | - | - | - | - | 47.52 ; 91.87 | 48.51 ;   93.99 | - | 26.0 ; 90.0 | 22.0 ; 90.0 | - | 19 ; 94 | 20 ; 93 | - | 28.00 ; 99.00 | 32.00 ; 95.00 | - | 46.00 ; 91.00 | 47.00 ; 92.00 | - | 25.00 ; 95.00 | 34.00 ; 94.00 | - | 22.97 ; 95.37 | 25.65 ; 97.88 | - | - | - | - | - | - | - |
| 18-54 | - | - | - | 66 (9.05) | 63 (8.64) | - | 8 (10.13) | 6 (7.59) | - | <5 | <5 | - | 316 (20.72) | 290 (19.02) | - | 139 (24.78) | 147 (26.20) | 0.01 | 39 (11.96) | 36 (11.04) | - | 5 (3.85) | 5 (3.85) | - | 78 (6.92) | 83 (7.36) | - | 248 (28.80) | 262 (30.43) | - | - | - | - | - | - | - |
| 55-64 | - | - | - | 152 (20.85) | 153 (20.99) | - | 21 (26.58) | 23 (29.11) | - | 18 (25.35) | 13 (18.31) | - | 328 (21.51) | 314 (20.59) | - | 166 (29.59) | 147 (26.20) | - | 82 (25.15) | 85 (26.07) | - | 21 (16.15) | 23 (17.69) | - | 223 (19.79) | 222 (19.70) | - | 252 (29.27) | 235 (27.29) | - | - | - | - | - | - | - |
| 65-74 | - | - | - | 286 (39.23) | 291 (39.92) | - | 32 (40.51) | 33 (41.77) | - | 24 (33.80) | 25 (35.21) | - | 499 (32.72) | 510 (33.44) | - | 156 (27.81) | 174 (31.02) | - | 127 (38.96) | 127 (38.96) | - | 40 (30.77) | 35 (26.92) | - | 459 (40.73) | 477 (42.32) | - | 203 (23.58) | 178 (20.67) | - | - | - | - | - | - | - |
| 75+ | - | - | - | 225 (30.86) | 222 (30.45) | - | 18 (22.78) | 17 (21.52) | - | 25 (35.21) | 30 (42.25) | - | 382 (25.05) | 411 (26.95) | - | 100 (17.83) | 93 (16.58) | - | 78 (23.93) | 78 (23.93) | - | 64 (49.23) | 67 (51.54) | - | 367 (32.56) | 345 (30.61) | - | 158 (18.35) | 186 (21.60) | - | - | - | - | - | - | - |
| **Sex** | | | | | | | | | | | | | | | | | | | | | | | | | | | | | | | | | | | | |
| Female | - | - | - | 265 (36.35) | 237 (32.51) | 0.08 | 32 (40.51) | 27 (34.18) | 0.13 | 25 (35.21) | 25 (35.21) | 0.00 | 398 (26.10) | 432 (28.33) | 0.05 | 229 (40.82) | 238 (42.42) | 0.03 | 91 (27.91) | 96 (29.45) | 0.03 | 51 (39.23) | 58 (44.62) | 0.11 | 320 (28.39) | 327 (29.02) | 0.01 | 332 (38.56) | 349 (40.53) | 0.04 | - | - | - | - | - | - |
| Male | - | - | - | 464 (63.65) | 492 (67.49) | - | 47 (59.49) | 52 (65.82) | - | 46 (64.79) | 46 (64.79) | - | 1127 (73.90) | 1093 (71.67) | - | 332 (59.18) | 323 (57.58) | - | 235 (72.09) | 230 (70.55) | - | 79 (60.77) | 72 (55.38) | - | 807 (71.61) | 800 (70.98) | - | 529 (61.44) | 512 (59.47) | - | - | - | - | - | - | - |
| **HbA1c (%)** | | | | | | | | | | | | | | | | | | | | | | | | | | | | | | | | | | | | |
| Mean | - | - | - | 7.56 | 7.50 | 0.04 | 7.94 | 8.01 | 0.05 | 8.04 | 7.88 | 0.09 | 7.64 | 7.34 | 0.19 | - | - | - | - | - | - | 7.35 | 7.18 | 0.12 | 8.09 | 8.08 | 0.00 | - | - | - | - | - | - | - | - | - |
| SD | - | - | - | 3.86 | 3.82 | - | 1.54 | 1.59 | - | 1.78 | 1.55 | - | 1.68 | 1.47 | - | - | - | - | - | - | - | 1.59 | 1.4 | - | 3.59 | 3.64 | - | - | - | - | - | - | - | - | - | - |
| Median | - | - | - | 7.09 | 6.91 | - | 7.67 | 7.62 | - | 7.6 | 7.6 | - | 7.1 | 7.01 | - | - | - | - | - | - | - | 7 | 6.7 | - | 7.82 | 7.73 | - | - | - | - | - | - | - | - | - | - |
| Q1 ; Q3 | - | - | - | 6.36 ; 8.37 | 6.36 ; 8.28 | - | 6.82 ; 9.01 | 6.84 ; 9.02 | - | 7.00 ; 8.60 | 6.80 ; 8.60 | - | 6.71 ; 8.08 | 6.5 ; 7.66 | - | - | - | - | - | - | - | 6.30 ; 7.80 | 6.30 ; 7.80 | - | 7.09 ; 8.83 | 7.09 ; 8.92 | - | - | - | - | - | - | - | - | - | - |
| Min ; Max | - | - | - | 5.17 ; 17.25 | 4.90 ; 14.04 | - | - | - | - | 5.20 ; 16.10 | 5.20 ;   12.30 | - | 5.3 ; 17.1 | 5.03 ; 13.93 | - | - | - | - | - | - | - | 5.50 ; 13.20 | 5.00 ; 13.10 | - | 5.08 ; 15.60 | 5.17 ; 17.06 | - | - | - | - | - | - | - | - | - | - |
| Missing | - | - | - | 383 (52.54) | 378 (51.85) | - | - | - | - | 1 | 1 | - | 1373 (90.03) | 1405 (92.13) | - | - | - | - | - | - | - | 79 (60.80) | 75 (57.70) | - | - | - | - | - | - | - | - | - | - | - | - | - |
| **Total cholesterol (mg/dl)** | | | | | | | | | | | | | | | | | | | | | | | | | | | | | | | | | | | | |
| Mean | - | - | - | 159.14 | 161.8 | 0.06 | 176.56 | 172.68 | - | 151.21 | 152.89 | 0.04 | 179.13 | 178.66 | 0.01 | - | - | - | - | - | - | 165.86 | 168.68 | 0.07 | 165.12 | 165.75 | 0.01 | - | - | - | - | - | - | - | - | - |
| SD | - | - | - | 44.8 | 45 | - | 38.89 | 40.7 | - | 43.77 | 41.96 | - | 40.72 | 41.68 | - | - | - | - | - | - | - | 42.12 | 41.53 | - | 45.38 | 45.37 | - | - | - | - | - | - | - | - | - | - |
| Median | - | - | - | 150.81 | 154.68 | - | 174 | 165 | - | 148 | 141.5 | - | 176.25 | 174.92 | - | - | - | - | - | - | - | 158 | 168 | - | 158.55 | 158.55 | - | - | - | - | - | - | - | - | - | - |
| Q1 ; Q3 | - | - | - | 127.61 ; 189.48 | 127.61 ; 189.48 | - | 149.00 ; 204.95 | 144.00 ; 189.00 | - | 118.00 ;   175.00 | 124.00 ;   183.00 | - | 153.83 ; 203.0 | 150.5 ; 203.88 | - | - | - | - | - | - | - | 138.00 ; 190.00 | 137.00 ; 195.00 | - | 135.35 ; 189.48 | 131.48 ; 189.48 | - | - | - | - | - | - | - | - | - | - |
| Min ; Max | - | - | - | 77.34 ; 305.49 | 73.47 ; 371.23 | - | - | - | - | 73.00 ; 267.00 | 80.00 ;   250.00 | - | 84.14 ; 329.0 | 94.71 ; 326.0 | - | - | - | - | - | - | - | 87.00 ; 304.00 | 90.00 ; 273.00 | - | 58.01 ; 510.44 | 69.61 ; 421.50 | - | - | - | - | - | - | - | - | - | - |
| Missing | - | - | - | 365 (50.07) | 369 (50.62) | - | 22 (27.85) | 27 (34.18) | - | 1 | 1 | - | 1394 (91.41) | 1419 (93.05) | - | - | - | - | - | - | - | 68 (52.30) | 59 (45.40) | - | - | - | - | - | - | - | - | - | - | - | - | - |
| **Low-density lipoprotein level (mg/dl)** | | | | | | | | | | | | | | | | | | | | | | | | | | | | | | | | | | | | |
| Mean | - | - | - | 90.47 | 93.41 | 0.08 | 102.3 | 102.81 | - | 78.71 | 76.74 | 0.06 | 108.57 | 106.83 | 0.06 | - | - | - | - | - | - | 88.32 | 95.6 | 0.22 | 87.92 | 89.1 | 0.03 | - | - | - | - | - | - | - | - | - |
| SD | - | - | - | 37.61 | 38.06 | - | 31.04 | 34.62 | - | 32.27 | 33.12 | - | 30.99 | 31.31 | - | - | - | - | - | - | - | 32.06 | 32.74 | - | 36.85 | 37.06 | - | - | - | - | - | - | - | - | - | - |
| Median | - | - | - | 81.21 | 85.07 | - | 100 | 96 | - | 70.4 | 67.2 | - | 108.8 | 102.2 | - | - | - | - | - | - | - | 83.5 | 97 | - | 81.01 | 81.21 | - | - | - | - | - | - | - | - | - | - |
| Q1 ; Q3 | - | - | - | 61.87 ; 112.14 | 65.74 ; 112.14 | - | 76.00 ; 124.00 | 81.00 ; 124.00 | - | 54.90 ; 107.00 | 53.00 ;   97.00 | - | 88.52 ; 127.0 | 85.33 ; 124.5 | - | - | - | - | - | - | - | 65.00 ; 106.50 | 72.00 ; 114.00 | - | 61.87 ; 105.38 | 62.26 ; 108.28 | - | - | - | - | - | - | - | - | - | - |
| Min ; Max | - | - | - | 15.47 ; 212.68 | 23.20 ; 297.76 | - | - | - | - | 24.40 ; 165.20 | 29.80 ;   165.80 | - | 48.0 ; 224.0 | 49.0 ; 211.0 | - | - | - | - | - | - | - | 31.00 ; 200.00 | 35.00 ; 187.00 | - | 15.85 ; 259.09 | 15.85 ; 235.89 | - | - | - | - | - | - | - | - | - | - |
| Missing | - | - | - | 370 (50.75) | 372 (51.03) | - | 22 (27.85) | 32 (40.51) | - | 4 | 3 | - | 1403 (92.00) | 1426 (93.51) | - | - | - | - | - | - | - | 66 (50.80) | 64 (49.20) | - | - | - | - | - | - | - | - | - | - | - | - | - |
| **High-density lipoprotein level (mg/dl)** | | | | | | | | | | | | | | | | | | | | | | | | | | | | | | | | | | | | |
| Mean | - | - | - | 44.82 | 44.93 | 0.01 | 44.58 | 43.67 | - | 40.54 | 40.83 | 0.03 | 46.28 | 48.48 | 0.16 | - | - | - | - | - | - | 44.5 | 44.86 | 0.03 | 43.07 | 42.65 | 0.02 | - | - | - | - | - | - | - | - | - |
| SD | - | - | - | 13.57 | 14.92 | - | 12.36 | 12.48 | - | 11.12 | 8.84 | - | 15.06 | 11.79 | - | - | - | - | - | - | - | 11.78 | 11.34 | - | 12.53 | 12.66 | - | - | - | - | - | - | - | - | - | - |
| Median | - | - | - | 42.54 | 41.38 | - | 41.3 | 40 | - | 38.5 | 40 | - | 44 | 47 | - | - | - | - | - | - | - | 41 | 43 | - | 42.54 | 42.54 | - | - | - | - | - | - | - | - | - | - |
| Q1 ; Q3 | - | - | - | 35.58 ; 51.43 | 34.80 ; 51.43 | - | 35.00 ; 54.00 | 34.00 ; 53.00 | - | 32.00 ; 45.00 | 34.00 ;   45.00 | - | 38.0 ; 51.01 | 39.0 ; 57.0 | - | - | - | - | - | - | - | 37.10 ; 50.45 | 37.00 ; 50.60 | - | 34.80 ; 50.27 | 34.80 ; 50.27 | - | - | - | - | - | - | - | - | - | - |
| Min ; Max | - | - | - | 15.08 ; 104.02 | 17.79 ; 118.33 | - | - | - | - | 18.00 ; 72.00 | 26.00 ;   73.00 | - | 17.33 ; 154.0 | 28.0 ; 97.0 | - | - | - | - | - | - | - | 21.00 ; 82.00 | 25.00 ; 75.00 | - | 15.47 ; 131.48 | 15.47 ; 150.81 | - | - | - | - | - | - | - | - | - | - |
| Missing | - | - | - | 366 (50.21) | 370 (50.75) | - | 24 (30.38) | 32 (40.51) | - | 1 | 2 | - | 1376 (90.23) | 1408 (92.33) | - | - | - | - | - | - | - | 66 (50.80) | 64 (49.20) | - | - | - | - | - | - | - | - | - | - | - | - | - |
| **Triglyceride level (mg/dl)** | | | | | | | | | | | | | | | | | | | | | | | | | | | | | | | | | | | | |
| Mean | - | - | - | 173.93 | 170.14 | 0.03 | 215.34 | 208.02 | - | 160.39 | 172.83 | 0.14 | 177.09 | 153.97 | 0.20 | - | - | - | - | - | - | 143.79 | 152.32 | 0.12 | 192.31 | 192.17 | 0.00 | - | - | - | - | - | - | - | - | - |
| SD | - | - | - | 128.52 | 115.85 | - | 110.93 | 122 | - | 81.32 | 92.31 | - | 137.96 | 82.76 | - | - | - | - | - | - | - | 65.85 | 72.61 | - | 130.08 | 149.59 | - | - | - | - | - | - | - | - | - | - |
| Median | - | - | - | 137.28 | 141.71 | - | 197 | 188 | - | 140.5 | 141 | - | 141 | 131.17 | - | - | - | - | - | - | - | 134 | 135 | - | 159.43 | 159.43 | - | - | - | - | - | - | - | - | - | - |
| Q1 ; Q3 | - | - | - | 101.86 ; 204.60 | 106.28 ; 197.51 | - | 143.00 ; 261.00 | 113.00 ; 258.00 | - | 94.00 ; 198.00 | 103.00 ;   240.00 | - | 96.93 ; 204.0 | 98.08 ; 199.0 | - | - | - | - | - | - | - | 92.00 ; 176.00 | 98.00 ; 187.00 | - | 115.14 ; 230.28 | 115.14 ; 221.43 | - | - | - | - | - | - | - | - | - | - |
| Min ; Max | - | - | - | 38.97 ; 1347.15 | 44.28 ; 1140.78 | - | - | - | - | 58.00 ; 424.00 | 45.00 ;   443.00 | - | 43.0 ; 1117.8 | 39.0 ; 480.81 | - | - | - | - | - | - | - | 51.00 ; 385.00 | 44.00 ; 391.00 | - | 44.28 ; 1700.54 | 35.43 ; 2674.81 | - | - | - | - | - | - | - | - | - | - |
| Missing | - | - | - | 364 (49.93) | 370 (50.75) | - | 23 (29.11) | 28 (35.44) | - | 1 | 2 | - | 1376 (90.23) | 1406 (92.20) | - | - | - | - | - | - | - | 63 (48.50) | 55 (42.30) | - | - | - | - | - | - | - | - | - | - | - | - | - |
| **Estimated Glomerular Filtration Rate (mL/min/1,73m^2^)** | | | | | | | | | | | | | | | | | | | | | | | | | | | | | | | | | | | | |
| Mean | - | - | - | 77.62 | 70.36 | 0.34 | 66.61 | 65.75 | - | 66.81 | 65.9 | 0.05 | - | - | - | - | - | - | - | - | - | 71.53 | 68.55 | 0.15 | 74.85 | 74.63 | 0.01 | - | - | - | - | - | - | - | - | - |
| SD | - | - | - | 18.59 | 23.59 | - | 21.9 | 23.58 | - | 18.68 | 21.14 | - | - | - | - | - | - | - | - | - | - | 21.74 | 17.12 | - | 18.54 | 21.31 | - | - | - | - | - | - | - | - | - | - |
| Median | - | - | - | 78.67 | 70.83 | - | 66.66 | 66.5 | - | 66.66 | 60.8 | - | - | - | - | - | - | - | - | - | - | 74.18 | 67.98 | - | 76.97 | 75.19 | - | - | - | - | - | - | - | - | - | - |
| Q1 ; Q3 | - | - | - | 64.50 ; 91.26 | 51.24 ; 89.15 | - | 48.85 ; 82.49 | 47.58 ; 86.12 | - | 53.50 ; 81.92 | 50.60 ;   84.60 | - | - | - | - | - | - | - | - | - | - | 53.81 ; 88.02 | 57.98 ; 81.78 | - | 61.12 ; 89.01 | 59.04 ; 91.28 | - | - | - | - | - | - | - | - | - | - |
| Min ; Max | - | - | - | 25.85 ; 124.74 | 15.20 ; 158.84 | - | - | - | - | 27.13 ; 105.98 | 34.81 ;   113.97 | - | - | - | - | - | - | - | - | - | - | 23.10 ; 108.50 | 33.70 ; 110.04 | - | 24.88 ; 132.33 | 21.72 ; 178.98 | - | - | - | - | - | - | - | - | - | - |
| Missing | - | - | - | 321 (44.03) | 318 (43.62) | - | 8 (10.13) | 16 (20.25) | - | 1 | 1 | - | - | - | - | - | - | - | - | - | - | 68 (52.30) | 60 (46.20) | - | - | - | - | - | - | - | - | - | - | - | - | - |
| **Creatinine (mg/dl)** | | | | | | | | | | | | | | | | | | | | | | | | | | | | | | | | | | | | |
| Mean | - | - | - | 0.93 | 1.08 | 0.44 | 1.11 | 1.18 | - | 1.09 | 1.07 | 0.05 | 1.07 | 1.06 | 0.02 | - | - | - | - | - | - | 1.02 | 0.99 | 0.08 | 0.98 | 1 | 0.01 | - | - | - | - | - | - | - | - | - |
| SD | - | - | - | 0.25 | 0.39 | - | 0.39 | 0.49 | - | 0.3 | 0.3 | - | 0.61 | 0.63 | - | - | - | - | - | - | - | 0.35 | 0.25 | - | 0.28 | 0.32 | - | - | - | - | - | - | - | - | - | - |
| Median | - | - | - | 0.92 | 1.01 | - | 1.1 | 1.06 | - | 1.02 | 1.06 | - | 0.91 | 0.94 | - | - | - | - | - | - | - | 1 | 0.95 | - | 0.95 | 0.94 | - | - | - | - | - | - | - | - | - | - |
| Q1 ; Q3 | - | - | - | 0.77 ; 1.08 | 0.81 ; 1.24 | - | 0.84 ; 1.28 | 0.87 ; 1.31 | - | 0.87 ; 1.22 | 0.83 ; 1.28 | - | 0.72 ; 1.13 | 0.73 ; 1.18 | - | - | - | - | - | - | - | 0.80 ; 1.12 | 0.82 ; 1.14 | - | 0.80 ; 1.12 | 0.77 ; 1.18 | - | - | - | - | - | - | - | - | - | - |
| Min ; Max | - | - | - | 0.36 ; 2.64 | 0.35 ; 3.07 | - | - | - | - | 0.60 ; 2.06 | 0.58 ; 1.83 | - | 0.46 ; 4.14 | 0.42 ; 5.51 | - | - | - | - | - | - | - | 0.45 ; 2.51 | 0.49 ; 1.86 | - | 0.35 ; 2.53 | 0.11 ; 2.73 | - | - | - | - | - | - | - | - | - | - |
| Missing | - | - | - | 321 (44.03) | 318 (43.62) | - | 8 (10.13) | 16 (20.25) | - | 1 | 1 | - | 1368 (89.70) | 1398 (91.67) | - | - | - | - | - | - | - | 68 (52.30) | 60 (46.20) | - | - | - | - | - | - | - | - | - | - | - | - | - |
| **Combined comorbidity score** | | | | | | | | | | | | | | | | | | | | | | | | | | | | | | | | | | | | |
| Mean | - | - | - | - | - | - | - | - | - | - | - | - | 2.97 | 2.88 | 0.05 | 1.09 | 1.02 | 0.05 | - | - | - | 4.32 | 4.55 | 0.05 | - | - | - | 2.86 | 2.91 | 0.03 | - | - | - | - | - | - |
| SD | - | - | - | - | - | - | - | - | - | - | - | - | 1.8 | 1.75 | - | 1.46 | 1.42 | - | - | - | - | 4.3 | 4.51 | - | - | - | - | 1.47 | 1.51 | - | - | - | - | - | - | - |
| Median | - | - | - | - | - | - | - | - | - | - | - | - | 3 | 3 | - | 1 | 1 | - | - | - | - | 5 | 6 | - | - | - | - | 3 | 3 | - | - | - | - | - | - | - |
| Q1 ; Q3 | - | - | - | - | - | - | - | - | - | - | - | - | 2.0 ; 4.0 | 2.0 ; 4.0 | - | 0 ; 2 | 0 ; 2 | - | - | - | - | 0.00 ; 8.00 | 0.00 ; 8.00 | - | - | - | - | 2.00 ; 4.00 | 2.00 ; 4.00 | - | - | - | - | - | - | - |
| Min ; Max | - | - | - | - | - | - | - | - | - | - | - | - | -1.0 ; 13.0 | -1.0 ; 14.0 | - | -1 ; 7 | -1 ; 9 | - | - | - | - | 0.00 ; 13.00 | -1.00 ; 16.00 | - | - | - | - | 1.00 ; 9.00 | 1.00 ; 12.00 | - | - | - | - | - | - | - |
| Missing | - | - | - | - | - | - | - | - | - | - | - | - | - | - | - | - | - | - | - | - | - | - | - | - | - | - | - | - | - | - | - | - | - | - | - | - |
| **Comorbidities and other medical conditions** | | | | | | | | | | | | | | | | | | | | | | | | | | | | | | | | | | | | |
| Diabetic retinopathy | - | - | - | 43 (5.90) | 51 (7.00) | 0.04 | <5 | <5 | 0.00 | 15 (21.13) | 15 (21.13) | 0.00 | 153 (10.03) | 111 (7.28) | 0.10 | 114 (20.32) | 115 (20.50) | 0.00 | 43 (13.19) | 47 (14.42) | 0.04 | 9 (6.92) | 8 (6.15) | 0.03 | 162 (14.37) | 160 (14.20) | 0.01 | 73 (8.48) | 76 (8.83) | 0.01 | - | - | - | - | - | - |
| Diabetic neuropathy | - | - | - | 17 (2.33) | 13 (1.78) | 0.04 | 59 (74.68) | 62 (78.48) | - | 17 (23.94) | 21 (29.58) | 0.13 | 33 (2.16) | 25 (1.64) | 0.04 | 99 (17.65) | 107 (19.07) | 0.04 | <5 | 6 (1.84) | 0.05 | <5 | 7 (5.38) | 0.11 | 20 (1.77) | 19 (1.69) | 0.01 | 42 (4.88) | 39 (4.53) | 0.02 | - | - | - | - | - | - |
| Diabetic nephropathy | - | - | - | 13 (1.78) | 20 (2.74) | 0.06 | 8 (10.13) | 8 (10.13) | 0.00 | 15 (21.13) | 6 (8.45) | 0.36 | 109 (7.15) | 88 (5.77) | 0.06 | 98 (17.47) | 93 (16.58) | 0.02 | 8 (2.45) | 7 (2.15) | 0.02 | 5 (3.85) | 5 (3.85) | 0.00 | 21 (1.86) | 23 (2.04) | 0.01 | 185 (21.49) | 189 (21.95) | 0.01 | - | - | - | - | - | - |
| Diabetes with peripheral circulatory disorders | - | - | - | 30 (4.12) | 32 (4.39) | 0.01 | - | - | - | 6 (8.45) | 7 (9.86) | 0.05 | 11 (0.72) | 11 (0.72) | 0.00 | 15 (2.67) | 11 (1.96) | 0.05 | <5 | <5 | 0.03 | 11 (8.46) | 18 (13.85) | 0.17 | 26 (2.31) | 29 (2.57) | 0.02 | 12 (1.39) | 15 (1.74) | 0.03 | - | - | - | - | - | - |
| Diabetic foot | - | - | - | 0 (0.00) | 0 (0.00) | - | 23 (29.11) | 22 (27.85) | - | <5 | <5 | 0.07 | 23 (1.51) | 19 (1.25) | 0.02 | - | - | - | - | - | - | <5 | <5 | 0.00 | 23 (2.04) | 31 (2.75) | 0.05 | <5 | <5 | 0.02 | - | - | - | - | - | - |
| Hypoglycemia | - | - | - | <5 | <5 | 0.02 | <5 | 0 (0.00) | - | <5 | <5 | 0.00 | 15 (0.98) | 10 (0.66) | 0.04 | 15 (2.67) | 16 (2.85) | 0.01 | <5 | <5 | 0.00 | 7 (5.38) | 7 (5.38) | 0.00 | 14 (1.24) | 17 (1.51) | 0.02 | <5 | 5 (0.58) | 0.02 | - | - | - | - | - | - |
| Hyperlipidemia | - | - | - | 209 (28.67) | 214 (29.36) | 0.02 | 62 (78.48) | 62 (78.48) | - | 25 (35.21) | 25 (35.21) | 0.00 | 1099 (72.07) | 1086 (71.21) | 0.02 | 548 (97.68) | 546 (97.33) | 0.02 | 67 (20.55) | 68 (20.86) | 0.01 | 66 (50.77) | 67 (51.54) | 0.02 | 336 (29.81) | 321 (28.48) | 0.03 | 450 (52.26) | 413 (47.97) | 0.09 | - | - | - | - | - | - |
| Ischemic heart disease | - | - | - | 327 (44.86) | 326 (44.72) | 0.00 | - | - | - | 38 (53.52) | 40 (56.34) | 0.06 | 1031 (67.61) | 1061 (69.57) | 0.04 | 392 (69.88) | 395 (70.41) | 0.01 | 173 (53.07) | 161 (49.39) | 0.07 | 58 (44.62) | 54 (41.54) | 0.06 | 604 (53.59) | 584 (51.82) | 0.04 | 455 (52.85) | 438 (50.87) | 0.04 | - | - | - | - | - | - |
| Stable angina | - | - | - | 124 (17.01) | 116 (15.91) | 0.03 | - | - | - | <5 | <5 | 0.00 | 695 (45.57) | 698 (45.77) | 0.00 | 86 (15.33) | 88 (15.69) | 0.01 | 62 (19.02) | 60 (18.40) | 0.02 | 52 (40.00) | 48 (36.92) | 0.06 | 268 (23.78) | 251 (22.27) | 0.04 | 118 (13.70) | 121 (14.05) | 0.01 | - | - | - | - | - | - |
| History of CABG or PTCA | - | - | - | 66 (9.05) | 71 (9.74) | 0.02 | 9 (11.39) | 15 (18.99) | - | 15 (21.13) | 17 (23.94) | 0.07 | 14 (0.92) | 17 (1.11) | 0.02 | 112 (19.96) | 114 (20.32) | 0.01 | 17 (5.21) | 16 (4.91) | 0.01 | <5 | <5 | 0.00 | 249 (22.09) | 242 (21.47) | 0.02 | 5 (0.58) | - | 0.05 | - | - | - | - | - | - |
| Any stroke | - | - | - | 75 (10.29) | 82 (11.25) | 0.03 | 9 (11.39) | 11 (13.92) | - | 8 (11.27) | 9 (12.68) | 0.04 | 138 (9.05) | 121 (7.93) | 0.04 | 72 (12.83) | 57 (10.16) | 0.08 | 28 (8.59) | 32 (9.82) | 0.04 | 10 (7.69) | 10 (7.69) | 0.00 | 124 (11.00) | 124 (11.00) | 0.00 | 47 (5.46) | 40 (4.65) | 0.04 | - | - | - | - | - | - |
| CHF | - | - | - | 729 (100.00) | 729 (100.00) | - | - | - | - | 0 (0.00) | 0 (0.00) | - | 1525 (100.00) | 1525 (100.00) | - | 561 (100.00) | 561 (100.00) | 0.00 | 326 (100.00) | 326 (100.00) | - | 0 (0.00) | 0 (0.00) | - | 1127 (100.00) | 1127 (100.00) | - | - | - | 0.00 | - | - | - | - | - | - |
| Peripheral Vascular disease or surgery | - | - | - | 74 (10.15) | 74 (10.15) | 0.00 | - | - | - | 6 (8.45) | 7 (9.86) | 0.05 | 116 (7.61) | 123 (8.07) | 0.02 | 155 (27.63) | 163 (29.06) | 0.03 | 227 (69.63) | 228 (69.94) | 0.01 | 14 (10.77) | 12 (9.23) | 0.05 | 213 (18.90) | 207 (18.37) | 0.01 | 33 (3.83) | 39 (4.53) | 0.03 | - | - | - | - | - | - |
| Hypertension | - | - | - | 453 (62.14) | 455 (62.41) | 0.01 | 77 (97.47) | 77 (97.47) | - | 37 (52.11) | 35 (49.30) | 0.06 | 1281 (84.00) | 1254 (82.23) | 0.05 | 483 (86.10) | 478 (85.20) | 0.03 | 73 (22.39) | 80 (24.54) | 0.05 | 96 (73.85) | 92 (70.77) | 0.07 | 613 (54.39) | 607 (53.86) | 0.01 | 682 (79.21) | 667 (77.47) | 0.04 | - | - | - | - | - | - |
| Chronic kidney disease | - | - | - | 10 (1.37) | 12 (1.65) | 0.02 | - | - | - | 12 (16.90) | 12 (16.90) | 0.00 | 154 (10.10) | 174 (11.41) | 0.04 | 23 (4.10) | 18 (3.21) | 0.05 | 16 (4.91) | 18 (5.52) | 0.03 | 9 (6.92) | 10 (7.69) | 0.03 | 32 (2.84) | 30 (2.66) | 0.01 | 76 (8.83) | 78 (9.06) | 0.01 | - | - | - | - | - | - |
| **Number of antidiabetic substances at index date** | | | | | | | | | | | | | | | | | | | | | | | | | | | | | | | | | | | | |
| Mean | - | - | - | 2.18 | 2.25 | 0.07 | - | - | - | 2.37 | 2.31 | 0.06 | 1.74 | 1.63 | 0.11 | 2.04 | 2.05 | 0.01 | 2.17 | 2.19 | 0.02 | 1.76 | 1.84 | 0.07 | 2.04 | 2.06 | 0.02 | 2.32 | 2.26 | - | - | - | - | - | - | - |
| SD | - | - | - | 0.8 | 0.91 | - | - | - | - | 0.93 | 0.8 | - | 1.04 | 0.92 | - | 0.75 | 0.71 | - | 0.89 | 0.93 | - | 1.16 | 1.11 | - | 0.72 | 0.74 | - | 1.09 | 0.97 | - | - | - | - | - | - | - |
| Median | - | - | - | 2 | 2 | - | - | - | - | 2 | 2 | - | 1 | 1 | - | 2 | 2 | - | 2 | 2 | - | 2 | 2 | - | 2 | 2 | - | 2 | 2 | - | - | - | - | - | - | - |
| Q1 ; Q3 | - | - | - | 2.00 ; 3.00 | 2.00 ; 3.00 | - | - | - | - | 2.00 ; 3.00 | 2.00 ; 3.00 | - | 1.0 ; 2.0 | 1.0 ; 2.0 | - | 2 ; 3 | 2 ; 2 | - | 2.00 ; 3.00 | 2.00 ; 3.00 | - | 1.00 ; 3.00 | 1.00 ; 3.00 | - | 2.00 ; 2.00 | 2.00 ; 2.00 | - | 2.00 ; 3.00 | 2.00 ; 3.00 | - | - | - | - | - | - | - |
| Min ; Max | - | - | - | 1.00 ; 6.00 | 1.00 ; 6.00 | - | - | - | - | 1.00 ; 5.00 | 1.00 ; 4.00 | - | 1.0 ; 7.0 | 1.0 ; 6.0 | - | 1 ; 5 | 1 ; 4 | - | 1.00 ; 5.00 | 1.00 ; 6.00 | - | 0.00 ; 5.00 | 0.00 ; 5.00 | - | 1.00 ; 5.00 | 1.00 ; 5.00 | - | 1.00 ; 9.00 | 1.00 ; 6.00 | - | - | - | - | - | - | - |
| **Diabetes medication use** | | | | | | | | | | | | | | | | | | | | | | | | | | | | | | | | | | | | |
| Naive new use of antidiabetic drugs | - | - | - | 14 (1.92) | 14 (1.92) | 0.00 | - | - | - | 7 (9.86) | 5 (7.04) | 0.10 | 771 (50.56) | 850 (55.74) | 0.10 | 157 (27.99) | 164 (29.23) | 0.03 | 10 (3.07) | 10 (3.07) | 0.00 | 13 (10.00) | 13 (10.00) | 0.00 | 9 (0.80) | 8 (0.71) | 0.01 | 0 (0.00) | 0 (0.00) | 0.00 | - | - | - | - | - | - |
| Initiation of the study drug (empagliflozin/any SGLT-2 inhibitor/any DPP-4 inhibitor) as monotherapy | - | - | - | 11 (1.51) | 10 (1.37) | 0.01 | - | - | - | <5 | <5 | 0.00 | 680 (44.59) | 750 (49.18) | 0.09 | 75 (13.37) | 68 (12.12) | 0.04 | 9 (2.76) | 10 (3.07) | 0.02 | 61 (46.92) | 63 (48.46) | 0.03 | 7 (0.62) | 5 (0.44) | 0.02 | 155 (18.00) | 164 (19.05) | 0.03 | - | - | - | - | - | - |
| Dual therapy with metformin (without the use of other antidiabetic drugs) | - | - | - | 0 (0.00) | 0 (0.00) | - | - | - | - | 20 (28.17) | 20 (28.17) | 0.00 | 66 (4.33) | 67 (4.39) | 0.00 | 60 (10.70) | 70 (12.48) | 0.06 | 14 (4.29) | 16 (4.91) | 0.03 | - | - | - | 37 (3.28) | 40 (3.55) | 0.01 | 352 (40.88) | 350 (40.65) | 0.00 | - | - | - | - | - | - |
| Sulfonylureas 2nd generation (concomitant initiation or current use) | - | - | - | 0 (0.00) | 0 (0.00) | - | - | - | - | 7 (9.86) | 10 (14.08) | 0.13 | - | - | - | 161 (28.70) | 159 (28.34) | 0.01 | 51 (15.64) | 49 (15.03) | 0.02 | 5 (3.85) | 7 (5.38) | 0.07 | 107 (9.49) | 108 (9.58) | 0.00 | - | - | - | - | - | - | - | - | - |
| Insulin (concomitant initiation or current use) | - | - | - | 38 (5.21) | 42 (5.76) | 0.02 | - | - | - | 24 (33.80) | 17 (23.94) | 0.22 | - | - | - | 55 (9.80) | 62 (11.05) | 0.04 | 54 (16.56) | 54 (16.56) | 0.00 | 58 (44.62) | 61 (46.92) | 0.05 | 336 (29.81) | 349 (30.97) | 0.03 | - | - | - | - | - | - | - | - | - |

**CABG** - coronary artery bypass grafting; **CHF** - congestive heart failure; **DPP-4i -** dipeptidyl peptidase-4 inhibitor; **HbA1c** - Glycated hemoglobin; **Max** - maximum; **Min** - minimum; **PTCA** - percutaneous transluminal coronary angioplasty; **Q1** - 1st quartile; **Q3** - 3rd quartile; **SD** - standard deviation; **SGLT2i** - sodium-glucose cotransporter-2 inhibitor; **Std Diff** - standardized difference.

The combined comorbidity score is a single numeric comorbidity score for predicting short and long-term mortality, by combining conditions in the Charlson and Elixhauser comorbidity measures. Presence of diagnosis codes indicating existence of certain conditions may themselves be indicators for other factors that are inversely associated with 1-year mortality or may reflect idiosyncrasies of administrative data which is why negative values can occur. However, the underlying diagnoses should not be regarded as having a preventive/protective effect. JJ Gagne et al. *A combined comorbidity score predicted mortality in elderly patients better than existing scores.* J Clin Epidemiol. 2011 July ; 64(7): 749–759. doi:10.1016/j.jclinepi.2010.10.004.

Lookback period for combined comorbidity score is ever before index date for Denmark, Finland, Norway, Sweden and UK THIN and 12 months prior to the index date for other countries.

Lookback period for comorbidities and diabetes medication use is ever before index date for Denmark, Finland, Norway, Sweden and UK THIN, 6 months prior to the index date for Germany and 12 months or ever before for UK CPRD depending on covariate. For other countries, it is 12 months prior to the index date.


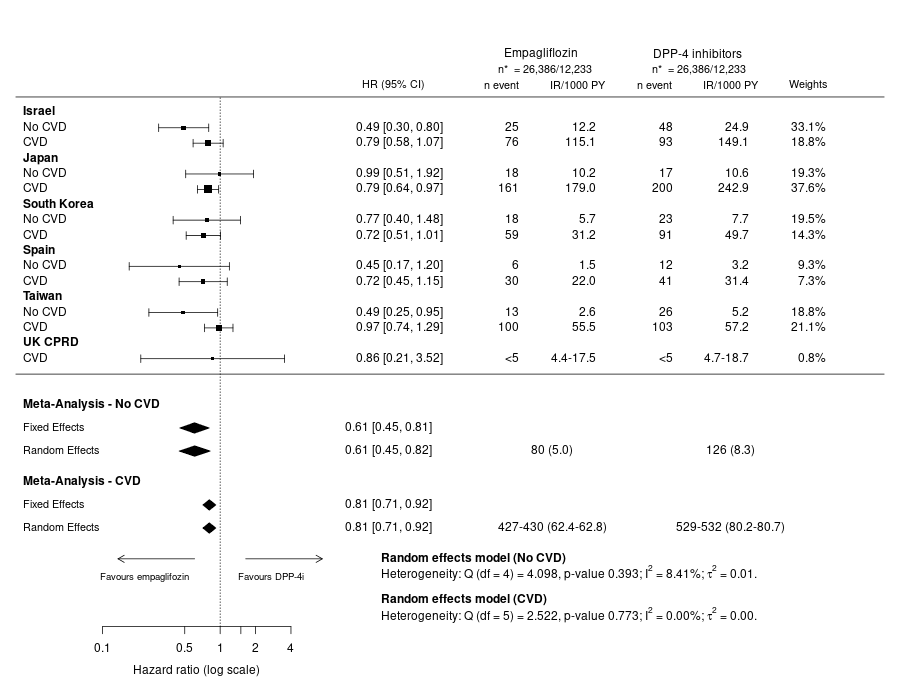


## Figure S1. Meta-analysis for hospitalization for heart failure (broad definition)^1^ in empagliflozin and DPP-4i initiators stratified by pre-existing cardiovascular disease (CVD)

Abbreviations: CI, confidence interval; CPRD, Clinical Practice Research Datalink; DPP-4i, dipeptidyl peptidase-4 inhibitors; HR, hazard ratio; PY, person-years; UK, United Kingdom

Analysis details: An as-treated approach was used and 100% grace period with no risk window was applied If countries had only one analysis (e.g. No CVD) then the other analysis (with CVD) is not displayed. Numbers <5 are not shown due to data protection, but they are included in meta-analysis. If values <5 exist, total number of events and incidence rates are presented as intervals. The number of countries included in each analysis therefore may vary.

^1^ Defined as any diagnosis of heart failure associated with hospitalizations, specialist outpatient and primary care encounters, and/or a dispensation/record of high-ceiling or loop diuretics. Includes Israel, Japan, South Korea, Spain, Taiwan and UK CPRD (CVD).

* Number of patients without/with CVD.


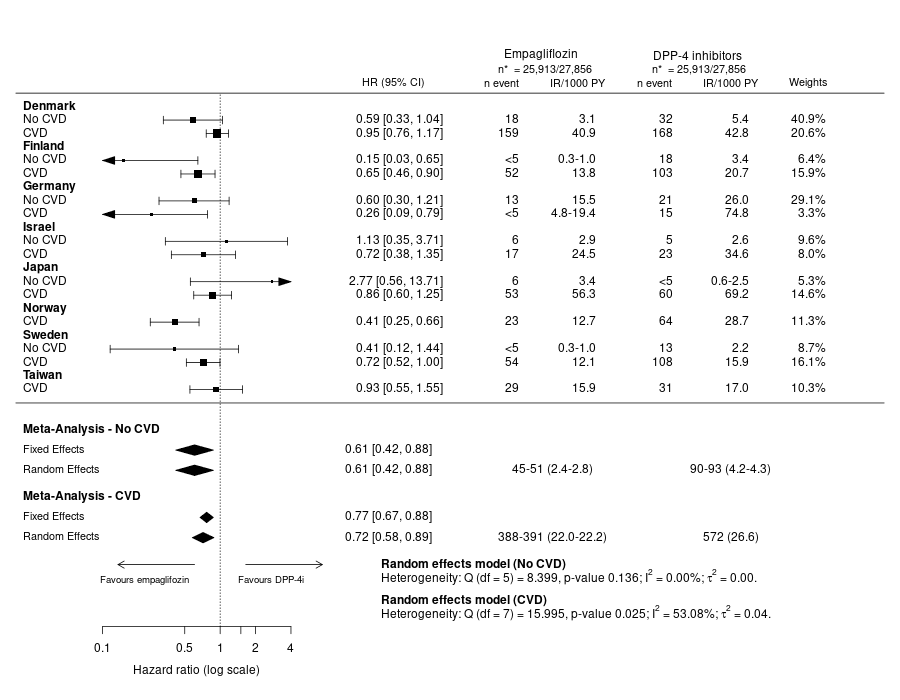


## Figure S2. Meta-analysis for hospitalization for heart failure (specific definition)^1^ in empagliflozin and DPP-4i initiators stratified by pre-existing cardiovascular disease (CVD)

Abbreviations: CI, confidence interval; CPRD, Clinical Practice Research Datalink; DPP-4i, dipeptidyl peptidase-4 inhibitors; HR, hazard ratio; PY, person-years

Analysis details: An as-treated approach was used and 100% grace period with no risk window was applied. If countries had only one analysis (e.g. No CVD) then the other analysis (with CVD) is not displayed. Numbers <5 are not shown due to data protection, but they are included in meta-analysis. If values <5 exist, total number of events and incidence rates are presented as intervals. The number of countries included in each analysis therefore may vary.

^1^ Defined as a diagnosis of heart failure during hospitalization. Includes Denmark, Finland, Germany, Israel, Japan, Norway (in with CVD), Sweden and Taiwan (CVD).

* Number of patients without/with CVD.


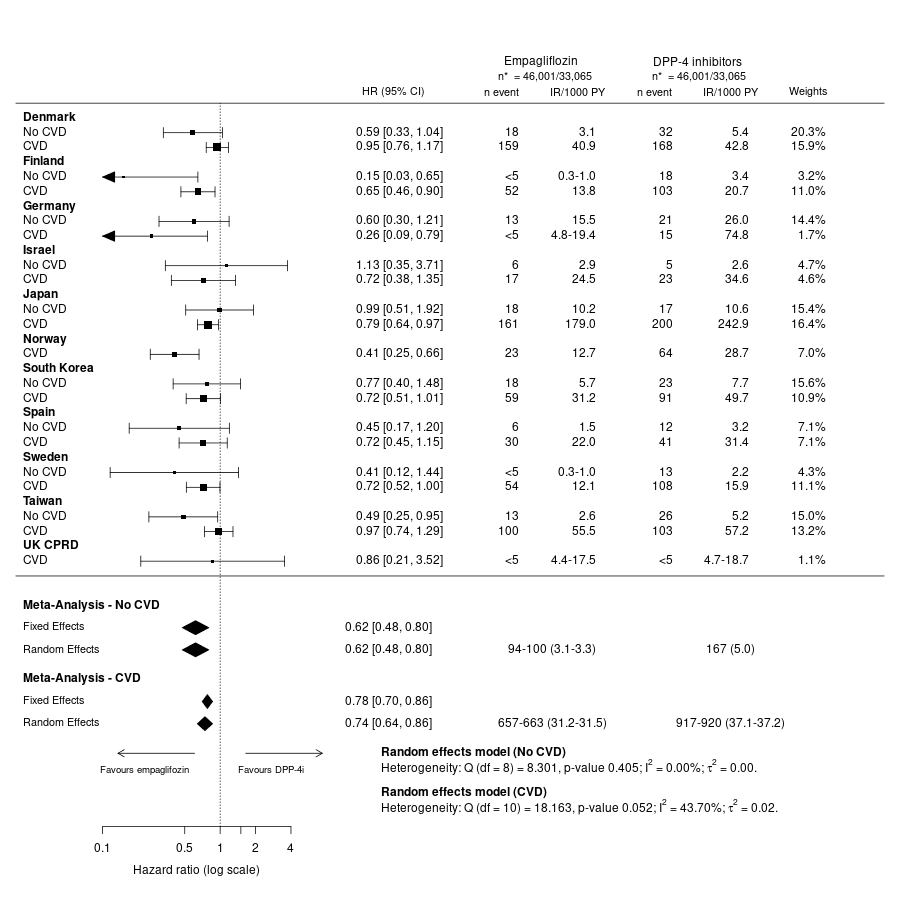


## Figure S3. Meta-analysis for hospitalization for heart failure (broad + specific)^1^ in empagliflozin and DPP-4i initiators stratified by pre-existing cardiovascular disease (CVD)

Abbreviations: CI, confidence interval; CPRD, Clinical Practice Research Datalink; DPP-4i, dipeptidyl peptidase-4 inhibitors; HR, hazard ratio; PY, person-years; UK, United Kingdom

Analysis details: An as-treated approach was used and 100% grace period with no risk window was applied. If countries had only one analysis (e.g. No CVD) then the other analysis (with CVD) is not displayed. Numbers <5 are not shown due to data protection, but they are included in meta-analysis. If values <5 exist, total number of events and incidence rates are presented as intervals. The number of countries included in each analysis therefore may vary.

^1^ Includes broad definition in Japan, South Korea, Spain, Taiwan and UK CPRD (CVD), and specific definition in Denmark, Finland, Germany, Israel, Norway (CVD), Sweden.

* Number of patients without/with CVD.


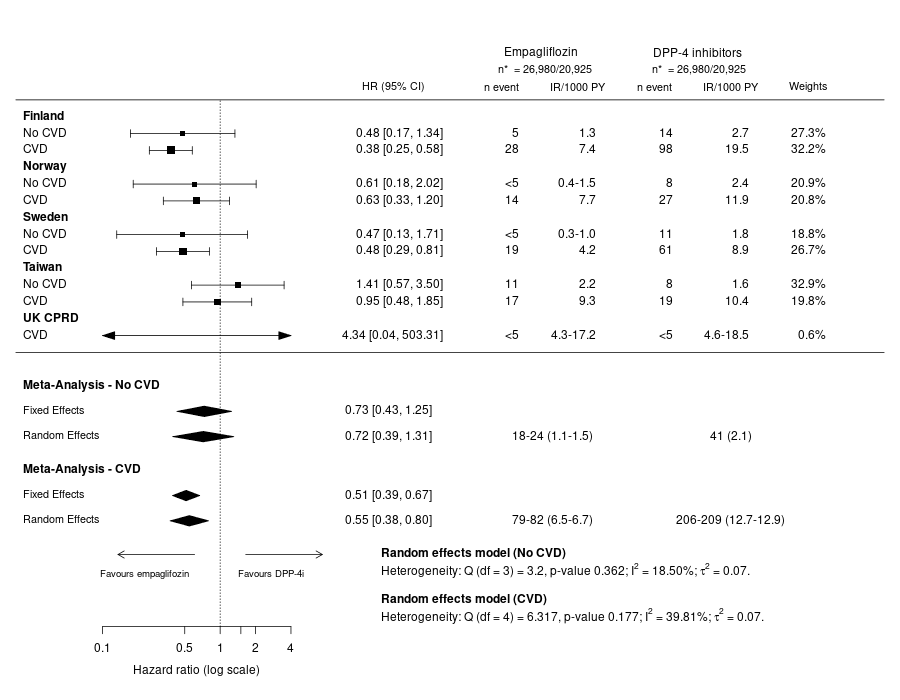


## Figure S4. Meta-analysis for cardiovascular mortality in empagliflozin and DPP-4i initiators stratified by pre-existing cardiovascular disease (CVD)

Abbreviations: CI, confidence interval; CPRD, Clinical Practice Research Datalink; DPP-4i, dipeptidyl peptidase-4 inhibitors; HR, hazard ratio; PY, person-years; UK, United Kingdom

Analysis details: An as-treated approach was used and 100% grace period with no risk window was applied. If countries had only one analysis (e.g. No CVD) then the other analysis (with CVD) is not displayed. Numbers <5 are not shown due to data protection, but they are included in meta-analysis. If values <5 exist, total number of events and incidence rates are presented as intervals. The number of countries included in each analysis therefore may vary.

* Number of patients without/with CVD.


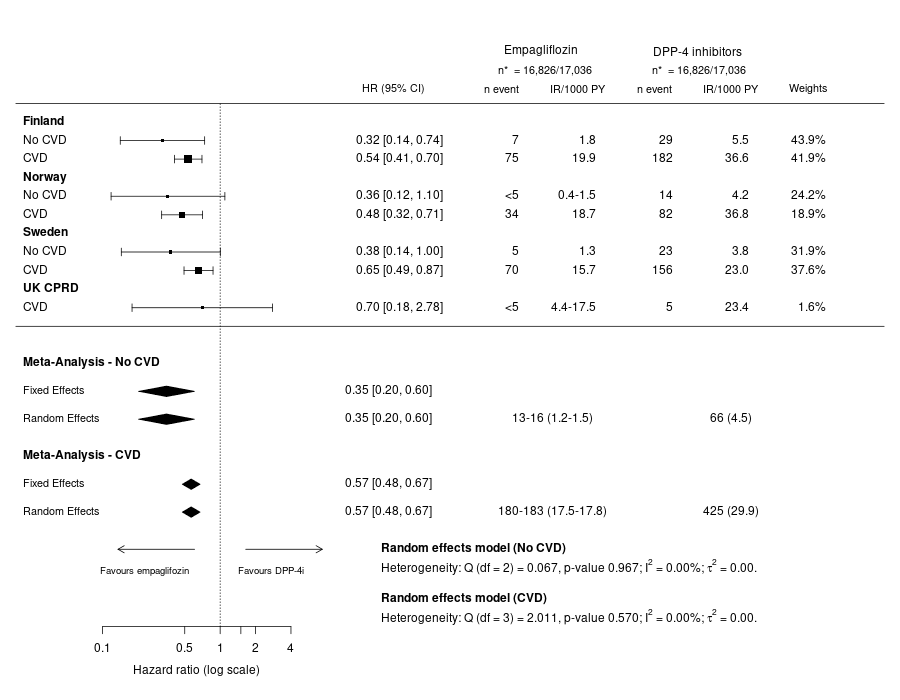


## Figure S5. Meta-analysis for composite outcome of hospitalization for heart failure or cardiovascular mortality in empagliflozin and DPP-4i initiators stratified by pre-existing cardiovascular disease (CVD)

Abbreviations: CI, confidence interval; CPRD, Clinical Practice Research Datalink; DPP-4i, dipeptidyl peptidase-4 inhibitors; HR, hazard ratio; PY, person-years; UK, United Kingdom

Analysis details: An as-treated approach was used and 100% grace period with no risk window was applied. If countries had only one analysis (e.g. No CVD) then the other analysis (with CVD) is not displayed. Numbers <5 are not shown due to data protection, but they are included in meta-analysis. If values <5 exist, total number of events and incidence rates are presented as intervals. The number of countries included in each analysis therefore may vary.

* Number of patients without/with CVD.


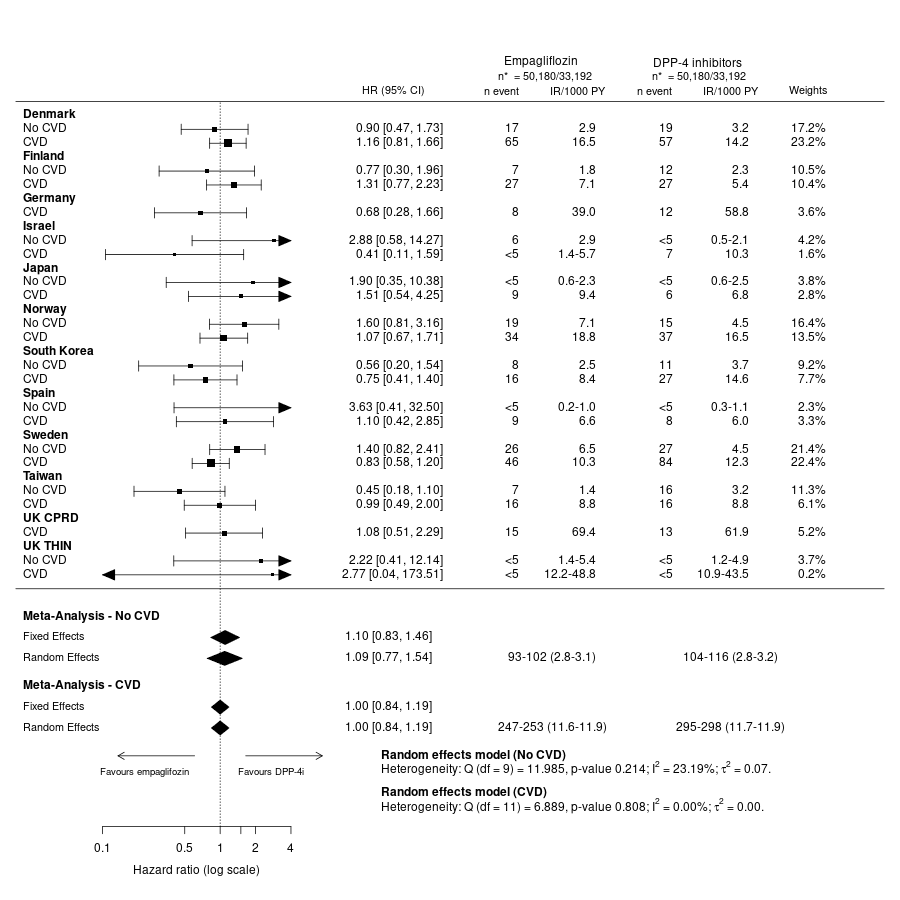


## Figure S6. Meta-analysis for myocardial infarction in empagliflozin and DPP-4i initiators stratified by pre-existing cardiovascular disease (CVD)

Abbreviations: CI, confidence interval; CPRD, Clinical Practice Research Datalink; DPP-4i, dipeptidyl peptidase-4 inhibitors; HR, hazard ratio; PY, person-years; UK, United Kingdom

Analysis details: An as-treated approach was used and 100% grace period with no risk window was applied. If countries had only one analysis (e.g. No CVD) then the other analysis (with CVD) is not displayed. Numbers <5 are not shown due to data protection, but they are included in meta-analysis. If values <5 exist, total number of events and incidence rates are presented as intervals. The number of countries included in each analysis therefore may vary.

* Number of patients without/with CVD.


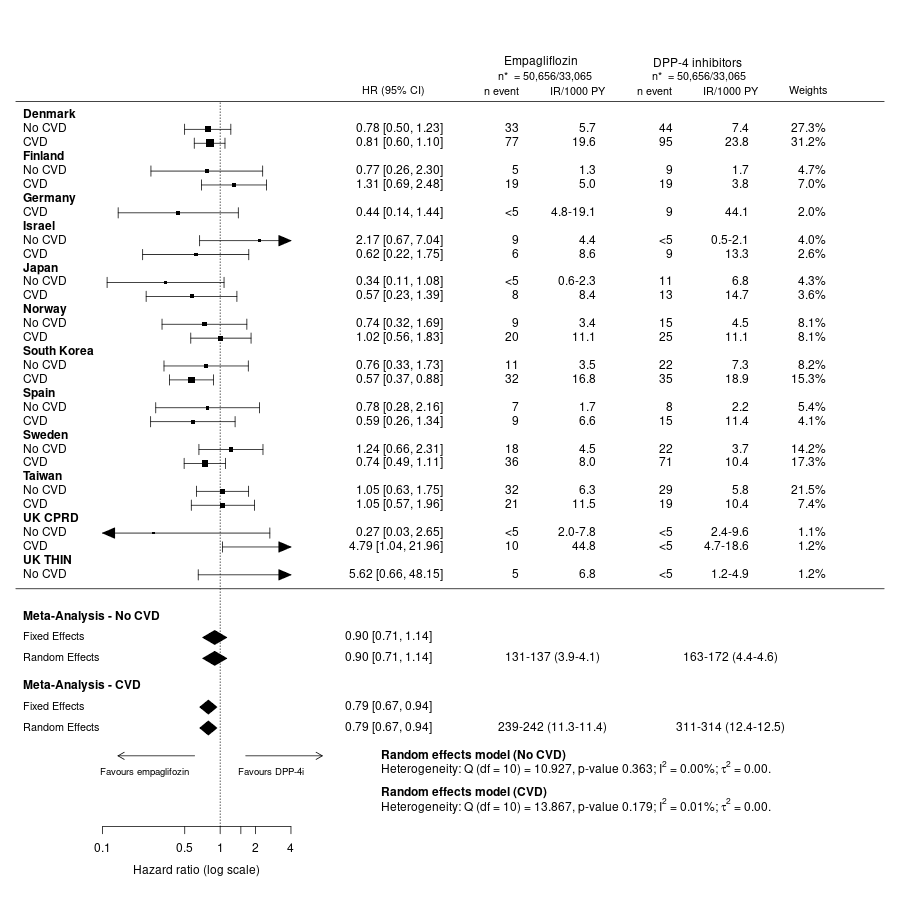


## Figure S7. Meta-analysis for stroke in empagliflozin and DPP-4i initiators stratified by pre-existing cardiovascular disease (CVD)

Abbreviations: CI, confidence interval; CPRD, Clinical Practice Research Datalink; DPP-4i, dipeptidyl peptidase-4 inhibitors; HR, hazard ratio; PY, person-years; THIN, The Health Improvement Network; UK, United Kingdom

Analysis details: An as-treated approach was used and 100% grace period with no risk window was applied. If countries had only one analysis (e.g. No CVD) then the other analysis (with CVD) is not displayed. Numbers <5 are not shown due to data protection, but they are included in meta-analysis. If values <5 exist, total number of events and incidence rates are presented as intervals. The number of countries included in each analysis therefore may vary.

* Number of patients without/with CVD.


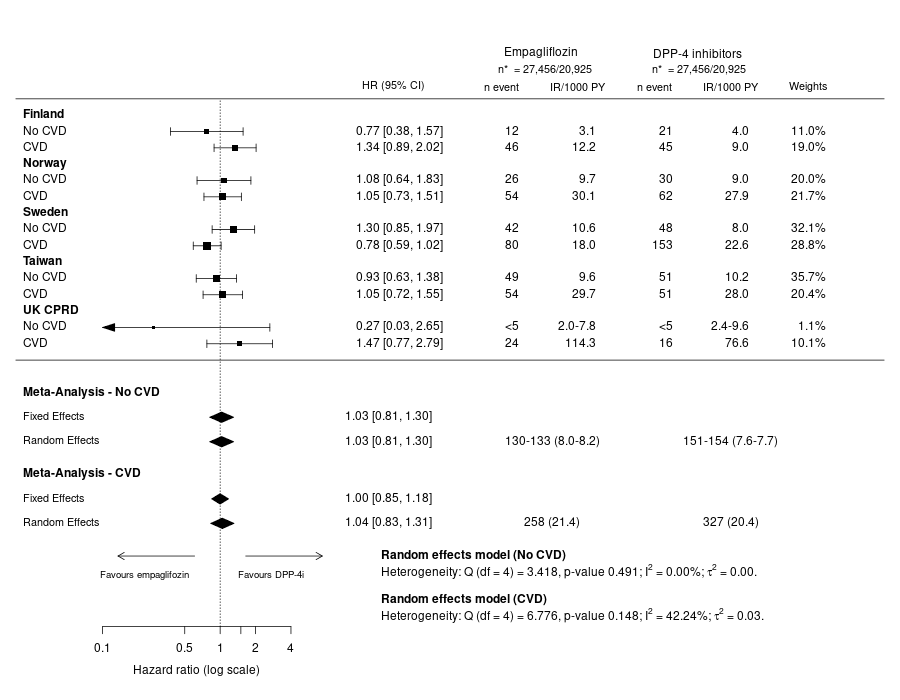


## Figure S8. Meta-analysis for 3-point MACE (myocardial infarction, stroke, and cardiovascular mortality) in empagliflozin and DPP-4i initiators stratified by pre-existing cardiovascular disease (CVD)

Abbreviations: CI, confidence interval; CPRD, Clinical Practice Research Datalink; DPP-4i, dipeptidyl peptidase-4 inhibitors; HR, hazard ratio; PY, person-years; UK, United Kingdom

Analysis details: An as-treated approach was used and 100% grace period with no risk window was applied. Numbers <5 are not shown due to data protection, but they are included in meta-analysis. If values <5 exist, total number of events and incidence rates are presented as intervals. The number of countries included in each analysis therefore may vary.

* Number of patients without/with CVD.


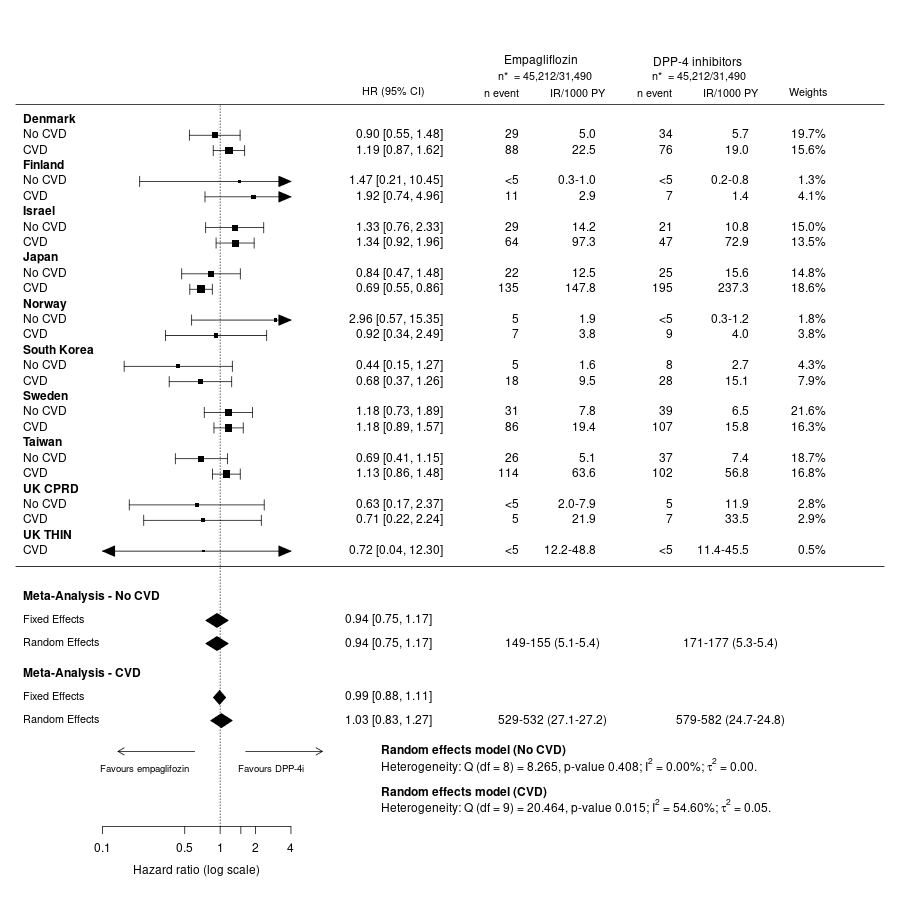


## Figure S9. Meta-analysis for coronary revascularization procedures in empagliflozin and DPP-4i initiators stratified by pre-existing cardiovascular disease (CVD)

Abbreviations: CI, confidence interval; CPRD, Clinical Practice Research Datalink; DPP-4i, dipeptidyl peptidase-4 inhibitors; HR, hazard ratio; PY, person-years; THIN, The Health Improvement Network; UK, United Kingdom

Analysis details: An as-treated approach was used and 100% grace period with no risk window was applied. If countries had only one analysis (e.g. No CVD) then the other analysis (with CVD) is not displayed. Numbers <5 are not shown due to data protection, but they are included in meta-analysis. If values <5 exist, total number of events and incidence rates are presented as intervals. The number of countries included in each analysis therefore may vary.

* Number of patients without/with CVD.


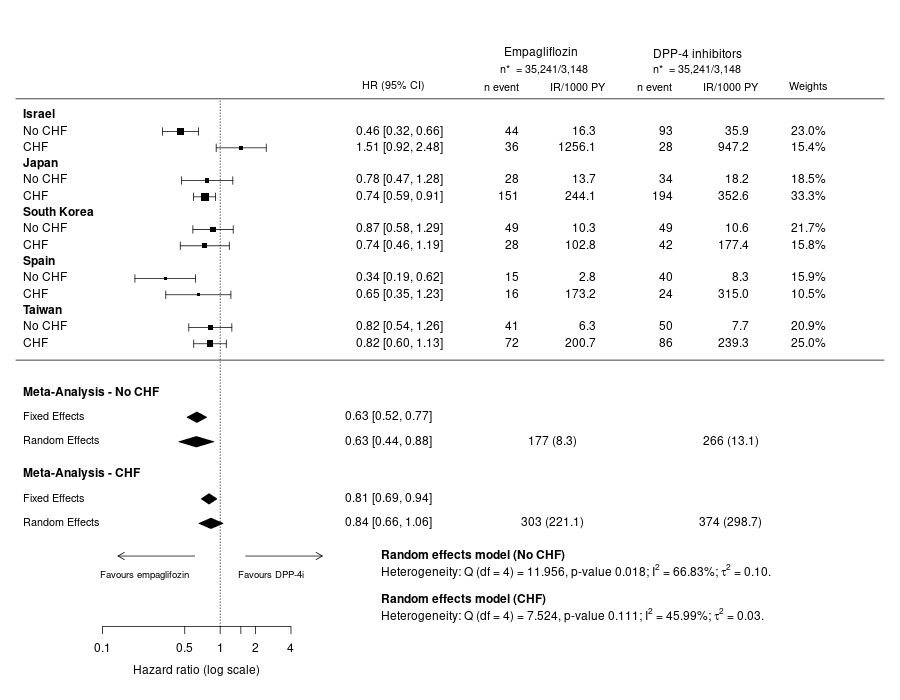


## Figure S10. Meta-analysis for hospitalization for heart failure (broad definition)^1^ in empagliflozin and DPP-4i initiators stratified by pre-existing heart failure (HF)

Abbreviations: CI, confidence interval; CPRD, Clinical Practice Research Datalink; DPP-4i, dipeptidyl peptidase-4 inhibitors; HR, hazard ratio; PY, person-years

Analysis details: An as-treated approach was used and 100% grace period with no risk window was applied. If countries had only one analysis (e.g. No HF) then the other analysis (with HF) is not displayed. Numbers <5 are not shown due to data protection, but they are included in meta-analysis. If values <5 exist, total number of events and incidence rates are presented as intervals. The number of countries included in each analysis therefore may vary.

^1^ Defined as any diagnosis of heart failure associated with hospitalizations, specialist outpatient and primary care encounters, and/or a dispensation/record of high-ceiling or loop diuretics. Includes Israel, Japan, South Korea, Spain and Taiwan.

* Number of patients without/with HF.


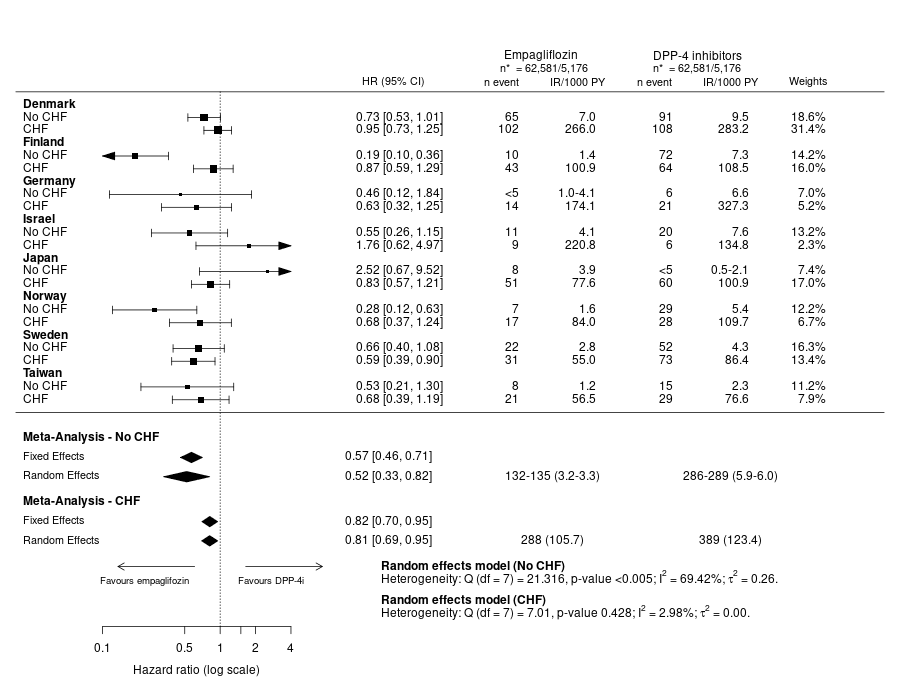


## Figure S11. Meta-analysis for hospitalization for heart failure (specific definition)^1^ in empagliflozin and DPP-4i initiators stratified by pre-existing heart failure (HF)

Abbreviations: CI, confidence interval; CPRD, Clinical Practice Research Datalink; DPP-4i, dipeptidyl peptidase-4 inhibitors; HR, hazard ratio; PY, person-years

Analysis details: An as-treated approach was used and 100% grace period with no risk window was applied. Numbers <5 are not shown due to data protection, but they are included in meta-analysis. If values <5 exist, total number of events and incidence rates are presented as intervals. The number of countries included in each analysis therefore may vary.

^1^ Defined as a diagnosis of heart failure during hospitalization or heart failure diagnosis during hospitalization that led to hospitalization, required most healthcare resources, or was coded as the main disease on the hospital claim. Includes Denmark, Finland, Germany, Israel, Japan, Norway, Sweden and Taiwan.

* Number of patients without/with HF.


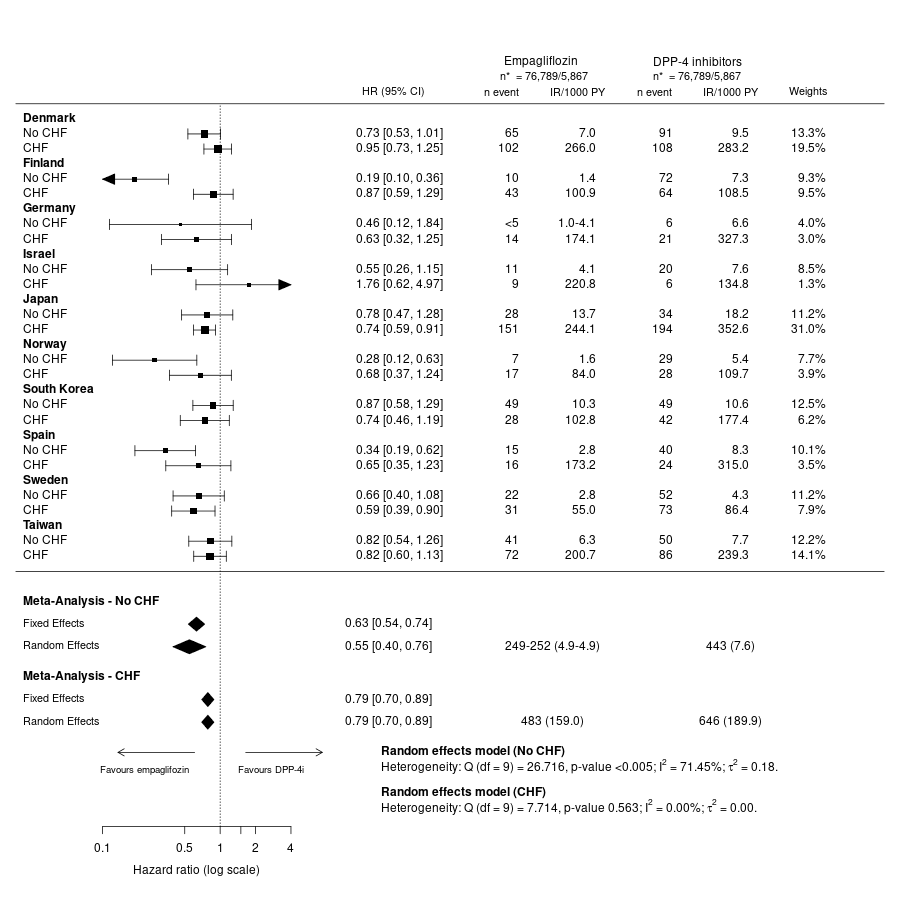


## Figure S12. Meta-analysis for hospitalization for heart failure (broad + specific)^1^ in empagliflozin and DPP-4i initiators stratified by pre-existing heart failure (HF)

Abbreviations: CI, confidence interval; CPRD, Clinical Practice Research Datalink; DPP-4i, dipeptidyl peptidase-4 inhibitors; HR, hazard ratio; PY, person-years

Analysis details: An as-treated approach was used and 100% grace period with no risk window was applied. Numbers <5 are not shown due to data protection, but they are included in meta-analysis. If values <5 exist, total number of events and incidence rates are presented as intervals. The number of countries included in each analysis therefore may vary.

^1^ Includes broad definition in Japan, South Korea, Spain and Taiwan and specific definition in Denmark, Finland, Germany, Israel, Sweden and Norway.

* Number of patients without/with HF.


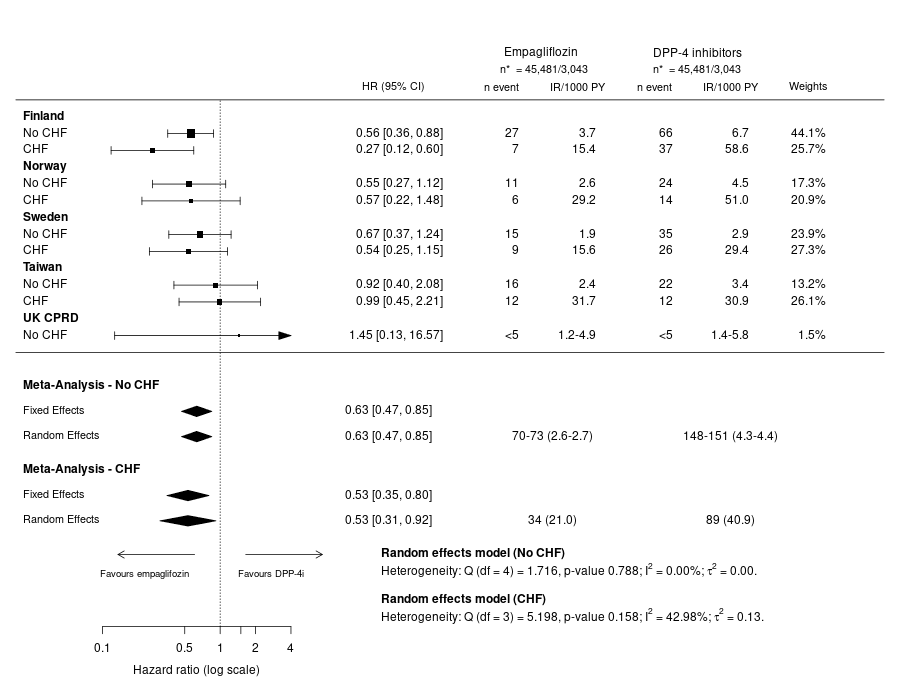


## Figure S13. Meta-analysis for cardiovascular mortality in empagliflozin and DPP-4i initiators stratified by pre-existing heart failure (HF)

Abbreviations: CI, confidence interval; CPRD, Clinical Practice Research Datalink; DPP-4i, dipeptidyl peptidase-4 inhibitors; HR, hazard ratio; PY, person-years; UK, United Kingdom

Analysis details: An as-treated approach was used and 100% grace period with no risk window was applied. If countries had only one analysis (e.g. No HF) then the other analysis (with HF) is not displayed. Numbers <5 are not shown due to data protection, but they are included in meta-analysis. If values <5 exist, total number of events and incidence rates are presented as intervals. The number of countries included in each analysis therefore may vary.

* Number of patients without/with HF.


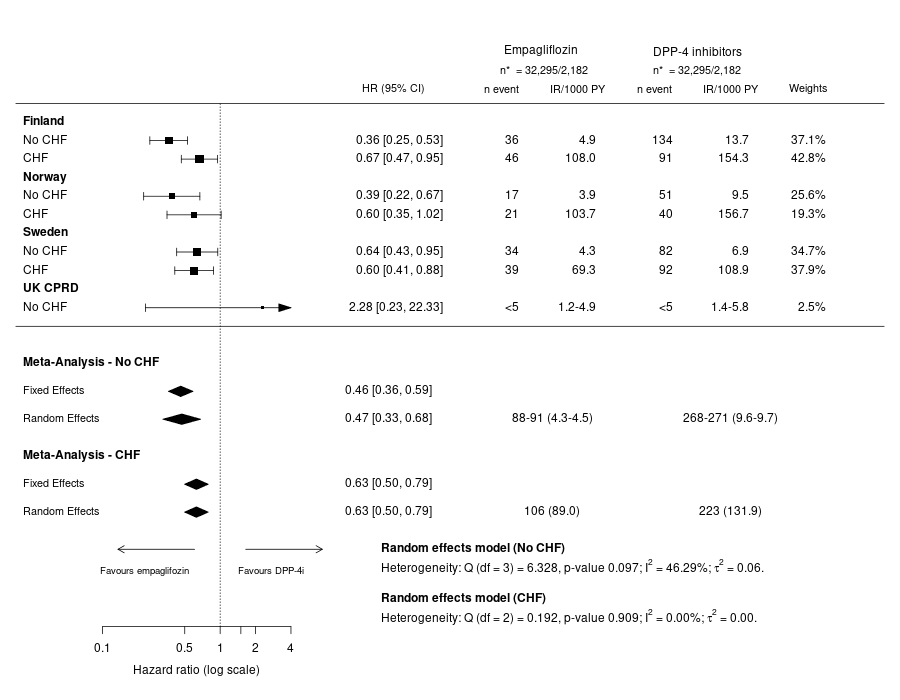


## Figure S14. Meta-analysis for hospitalization for heart failure or cardiovascular mortality in empagliflozin and DPP-4i initiators stratified by pre-existing heart failure (HF)

Abbreviations: CI, confidence interval; CPRD, Clinical Practice Research Datalink; DPP-4i, dipeptidyl peptidase-4 inhibitors; HR, hazard ratio; PY, person-years; UK, United Kingdom

Analysis details: An as-treated approach was used and 100% grace period with no risk window was applied. . If countries had only one analysis (e.g. No HF) then the other analysis (with HF) is not displayed. Numbers <5 are not shown due to data protection, but they are included in meta-analysis. If values <5 exist, total number of events and incidence rates are presented as intervals. The number of countries included in each analysis therefore may vary.

* Number of patients without/with HF.


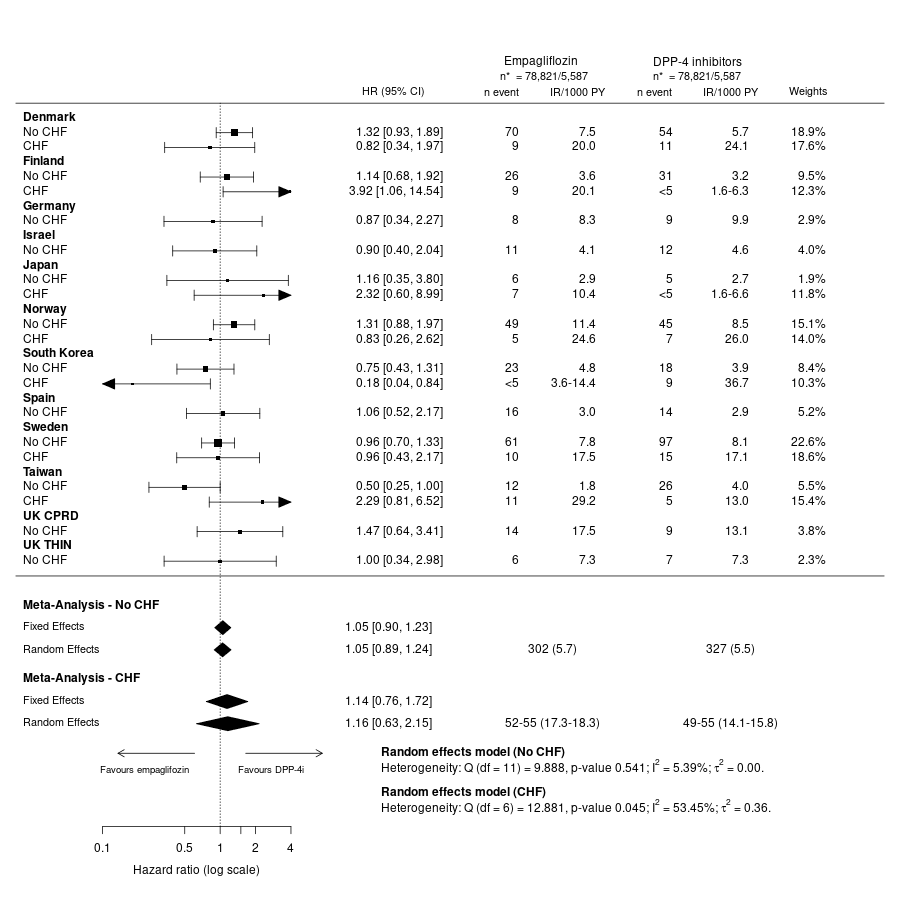


## Figure S15. Meta-analysis for myocardial infarction in empagliflozin and DPP-4i initiators stratified by pre-existing heart failure (HF)

Abbreviations: CI, confidence interval; CPRD, Clinical Practice Research Datalink; DPP-4i, dipeptidyl peptidase-4 inhibitors; HR, hazard ratio; PY, person-years; THIN, The Health Improvement Network; UK, United Kingdom

Analysis details: An as-treated approach was used and 100% grace period with no risk window was applied. . If countries had only one analysis (e.g. No HF) then the other analysis (with HF) is not displayed. Numbers <5 are not shown due to data protection, but they are included in meta-analysis. If values <5 exist, total number of events and incidence rates are presented as intervals. The number of countries included in each analysis therefore may vary.

* Number of patients without/with HF.


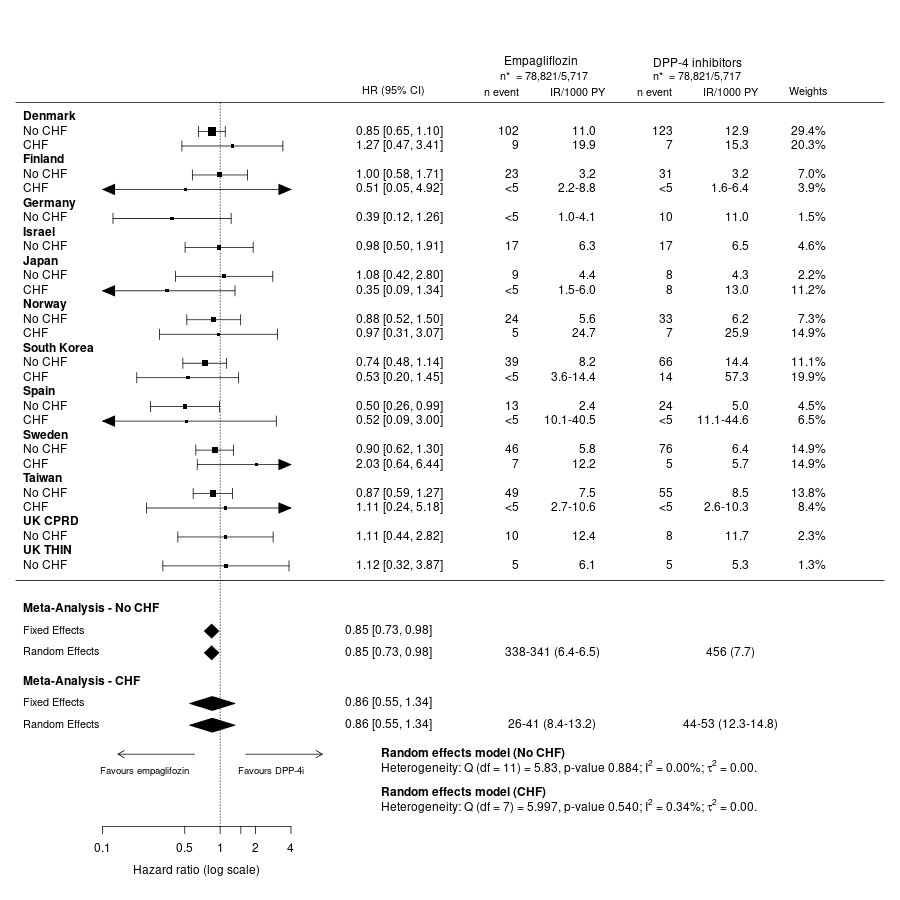


## Figure S16. Meta-analysis for stroke in empagliflozin and DPP-4i initiators stratified by pre-existing heart failure (HF)

Abbreviations: CI, confidence interval; CPRD, Clinical Practice Research Datalink; DPP-4i, dipeptidyl peptidase-4 inhibitors; HR, hazard ratio; PY, person-years; THIN, The Health Improvement Network; UK, United Kingdom

Analysis details: An as-treated approach was used and 100% grace period with no risk window was applied. . If countries had only one analysis (e.g. No HF) then the other analysis (with HF) is not displayed. Numbers <5 are not shown due to data protection, but they are included in meta-analysis. If values <5 exist, total number of events and incidence rates are presented as intervals. The number of countries included in each analysis therefore may vary.

* Number of patients without/with HF.


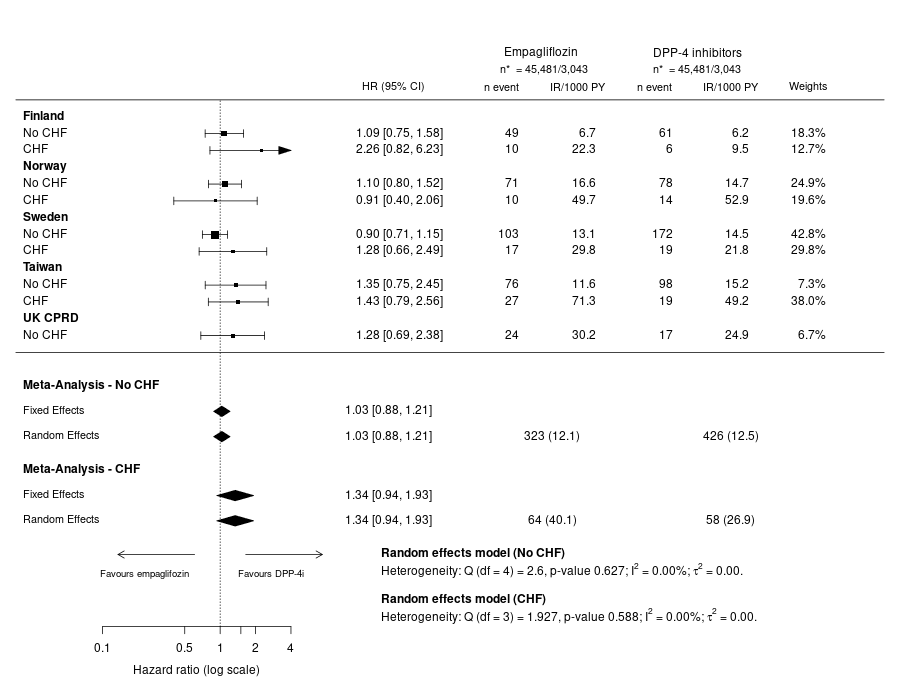


## Figure S17. Meta-analysis for 3-point MACE (myocardial infarction, stroke, and cardiovascular mortality) in empagliflozin and DPP-4i initiators stratified by pre-existing heart failure (HF)

Abbreviations: CI, confidence interval; CPRD, Clinical Practice Research Datalink; DPP-4i, dipeptidyl peptidase-4 inhibitors; HR, hazard ratio; PY, person-years; UK, United Kingdom

Analysis details: An as-treated approach was used and 100% grace period with no risk window was applied. If countries had only one analysis (e.g. No HF) then the other analysis (with HF) is not displayed. Numbers <5 are not shown due to data protection, but they are included in meta-analysis. If values <5 exist, total number of events and incidence rates are presented as intervals. The number of countries included in each analysis therefore may vary.

* Number of patients without/with HF.


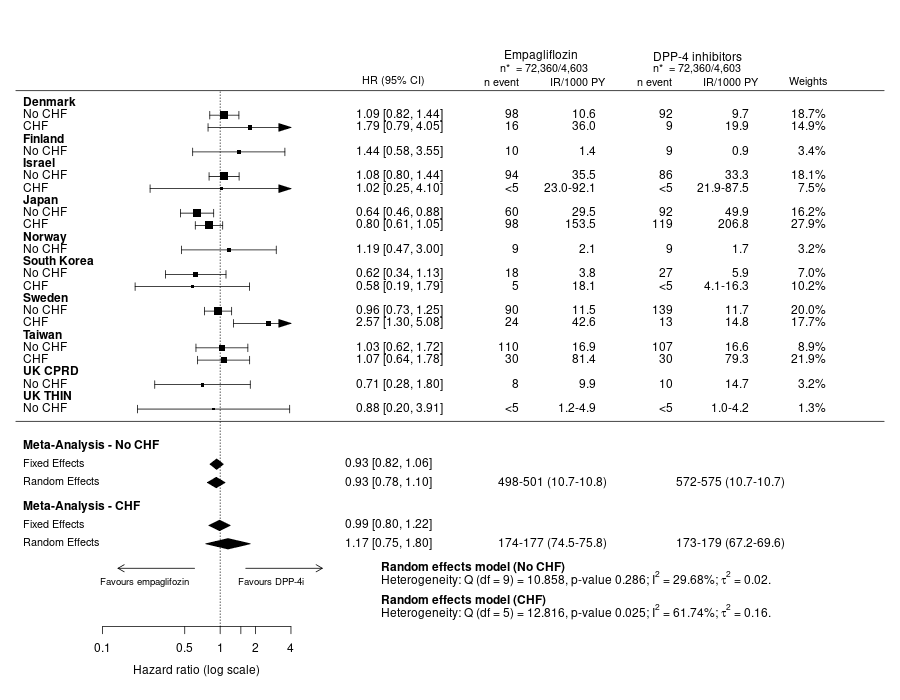


## Figure S18. Meta-analysis for coronary revascularization procedures in empagliflozin and DPP-4i initiators stratified by pre-existing heart failure (HF)

Abbreviations: CI, confidence interval; CPRD, Clinical Practice Research Datalink; DPP-4i, dipeptidyl peptidase-4 inhibitors; HR, hazard ratio; PY, person-years; THIN, The Health Improvement Network; UK, United Kingdom

Analysis details: An as-treated approach was used and 100% grace period with no risk window was applied. If countries had only one analysis (e.g. No HF) then the other analysis (with HF) is not displayed. Numbers <5 are not shown due to data protection, but they are included in meta-analysis. If values <5 exist, total number of events and incidence rates are presented as intervals. The number of countries included in each analysis therefore may vary.

* Number of patients without/with HF.

## REFERENCES

1. Lynge E, Sandegaard JL, Rebolj M. The Danish National Patient Register. Scand J Public Health 2011 Jul;39(7 Suppl):30–3.

2. Kildemoes HW, Sørensen HT, Hallas J. The Danish National Prescription Registry. Scand J Public Health 2011 Jul;39(7 Suppl):38–41.

3. Helweg-Larsen K. The Danish Register of Causes of Death. Scand J Public Health 2011 Jul;39(7 Suppl):26–9.

4. Regionernes Kliniske Kvalitetsudviklingsprogram (RKKP). Dansk Diabetes Database: Dansk Voksen Diabetes Database (DVDD) - almen praksis. National årsrapport 2019/2020. 1. juli 2019 – 30. juni 2020. [Internet]. 2020 Dec p. 28. Available from: https://www.sundhed.dk/content/cms/87/4687_aarsrapport_diabetes_2019_20_endelig_praksis.pdf

5. National Institute of Health and Welfare (HILMO) in Finnish: [Tunnusluvut ikäryhmittäin - Somaattinen erikoissairaanhoito alueittain (SHP) - THL kuutio- ja tiivistekäyttöliittymä] [Internet]. [cited 2019 May 9]. Available from: https://sampo.thl.fi/pivot/prod/fi/thil/perus03j/summary_summaryperus031

6. Social Insurance Institution. Finnish Prescription Register: Quality description 2019. (In Finnish: Laatuseloste 2019: Tilasto korvatuista resepteistä) [in Finnish] [Internet]. 2019 [cited 2020 Feb 18]. Available from: http://www.kela.fi/laatuseloste-2015-tilasto-korvatuista-resepteista#4

7. Pajunen A. Statistics Finland - Quality Description:Causes of death 2017 [Internet]. [cited 2020 Jan 6]. Available from: https://www.stat.fi/til/ksyyt/2017/ksyyt_2017_2018-12-17_laa_001_en.html

8. Ludvigsson JF, Andersson E, Ekbom A, Feychting M, Kim JL, Reuterwall C, et al. External review and validation of the Swedish national inpatient register. BMC Public Health 2011 Jun 9;11:450.

9. Wettermark B, Hammar N, Fored CM, MichaelFored C, Leimanis A, Otterblad Olausson P, et al. The new Swedish Prescribed Drug Register--opportunities for pharmacoepidemiological research and experience from the first six months. Pharmacoepidemiol Drug Saf 2007 Jul;16(7):726–35.

10. Brooke HL, Talbäck M, Hörnblad J, Johansson LA, Ludvigsson JF, Druid H, et al. The Swedish cause of death register. Eur J Epidemiol 2017 Sep;32(9):765–73.

11. Elfgren IMH, Grodzinsky E, Törnvall E. The Swedish National Diabetes Register in clinical practice and evaluation in primary health care. Primary Health Care Research & Development. 2016 Nov;17(6):549–58.

12. Herrett E, Gallagher AM, Bhaskaran K, Forbes H, Mathur R, van Staa T, et al. Data Resource Profile: Clinical Practice Research Datalink (CPRD). Int J Epidemiol 2015 Jun;44(3):827–36.
